# Tissue extracellular matrix hydrogels as alternatives to Matrigel for culturing gastrointestinal organoids

**DOI:** 10.1038/s41467-022-29279-4

**Published:** 2022-03-30

**Authors:** Suran Kim, Sungjin Min, Yi Sun Choi, Sung-Hyun Jo, Jae Hun Jung, Kyusun Han, Jin Kim, Soohwan An, Yong Woo Ji, Yun-Gon Kim, Seung-Woo Cho

**Affiliations:** 1 Department of Biotechnology, Yonsei University, Seoul, 03722 Republic of Korea; 2 Department of Chemical Engineering, Soongsil University, Seoul, 06978 Republic of Korea; 3 Institute of Vision Research, Department of Ophthalmology, Yonsei University College of Medicine, Seoul, 06229 Republic of Korea; 4 Department of Ophthalmology, National Health Insurance Service Ilsan Hospital, Goyang, 10444 Republic of Korea; 5 Center for Nanomedicine, Institute for Basic Science (IBS), Seoul, 03722 Republic of Korea; 6 Graduate Program of Nano Biomedical Engineering (NanoBME), Advanced Science Institute, Yonsei University, Seoul, 03722 Republic of Korea

**Keywords:** Stem-cell biotechnology, Tissue engineering, Adult stem cells, RNA sequencing, Proteomic analysis

## Abstract

Matrigel, a mouse tumor extracellular matrix protein mixture, is an indispensable component of most organoid tissue culture. However, it has limited the utility of organoids for drug development and regenerative medicine due to its tumor-derived origin, batch-to-batch variation, high cost, and safety issues. Here, we demonstrate that gastrointestinal tissue-derived extracellular matrix hydrogels are suitable substitutes for Matrigel in gastrointestinal organoid culture. We found that the development and function of gastric or intestinal organoids grown in tissue extracellular matrix hydrogels are comparable or often superior to those in Matrigel. In addition, gastrointestinal extracellular matrix hydrogels enabled long-term subculture and transplantation of organoids by providing gastrointestinal tissue-mimetic microenvironments. Tissue-specific and age-related extracellular matrix profiles that affect organoid development were also elucidated through proteomic analysis. Together, our results suggest that extracellular matrix hydrogels derived from decellularized gastrointestinal tissues are effective alternatives to the current gold standard, Matrigel, and produce organoids suitable for gastrointestinal disease modeling, drug development, and tissue regeneration.

## Abstract (web summary)

The culture of gastrointestinal organoids relies on Matrigel that has several drawbacks for clinical application. Here, the authors report the feasibility of gastrointestinal tissue-mimetic matrices as effective alternatives to Matrigel for organoid culture and transplantation.

## Introduction

Three-dimensional (3D) miniature organs of the gastrointestinal (GI) tract, called GI organoids, have been established from stem cells and are grown in extracellular matrix (ECM)-embedded in vitro culture systems^1–5^. Currently, the culture of most organoids, including GI organoids, relies on Matrigel, a commercialized matrix composed of various elements including laminin, collagen type IV, and growth factors. However, Matrigel has several undeniable drawbacks. As Matrigel is a raw material extracted from the Engelbreth–Holm–Swarm mouse sarcoma, it causes large batch-to-batch variation in cultured organoids. Given its origin, there is also a potential risk of transmission of animal pathogens^6,7^ that infect macrophages and affect the immune systems (e.g., lactate dehydrogenase-elevating virus^8,9^). In addition, Matrigel is primarily composed of tumor ECM components such as laminin which is highly expressed in several types of tumors^10,11^. As the matrisome, a collection of ECM and ECM-associated proteins, in tumors is substantially different from that of normal tissues^12^, tumor ECM-based Matrigel may not provide the tissue-specific microenvironments for GI organoids. For these reasons, many attempts have been made to develop alternatives to Matrigel^13–16^. For example, synthetic poly(ethylene glycol) (PEG) hydrogels modified with ECM peptides (e.g., RGD) and protease-degradable peptides (e.g., GPQGIWGQ) or natural hydrogels, such as alginate hydrogel or fibrin gel mixed with ECM proteins (e.g., laminin-111), have been tested as artificial 3D environments for GI organoids. Despite the obvious benefits of these engineered hydrogels in culturing GI organoids, they need improvements to fully reconstitute the biochemical ECM composition of the native tissue and promote organoid development and maturation.

Here, we develop a GI organoid culture platform that mimics the native GI ECM microenvironment using decellularization techniques. We investigate whether our GI ECM hydrogels could replace Matrigel in GI organoid culture by providing cell–matrix signals optimal for GI organoid development.

## Results

### Characterization of decellularized GI tissue-derived hydrogels

**Fig. 1:**
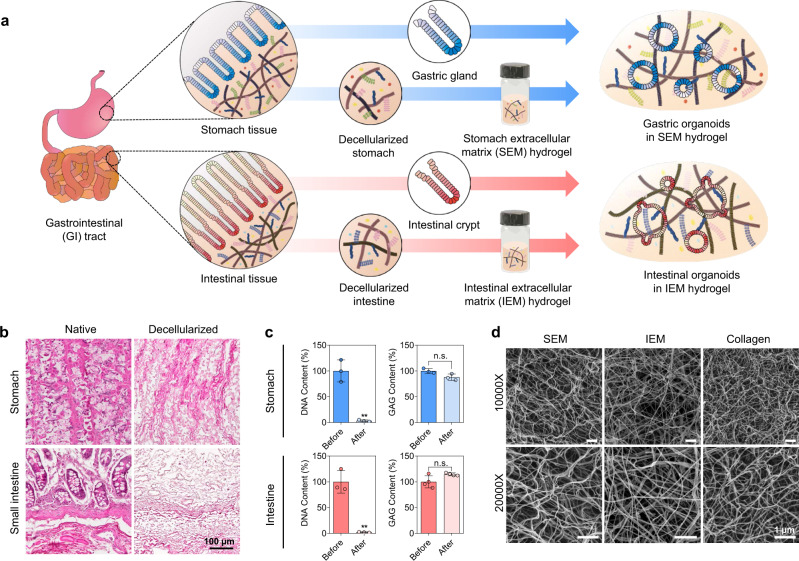
Preparation and characterization of GI tissue-derived ECM hydrogels for GI organoid culture. **a** Schematic illustration of the generation of GI organoids using ECM hydrogels (SEM and IEM) derived from the decellularized GI tract. **b** Hematoxylin and eosin (H&E) staining of the porcine stomach and intestinal tissues before and after decellularization (scale bar = 100 µm, independent experiments = 2). **c** DNA and glycosaminoglycans (GAG) content of porcine stomach and intestinal tissues before and after decellularization (Before versus After, **p = 0.0014 for DNA content of stomach; Before versus After, **p = 0.0015 for DNA content of intestine; *N* = 3 for SEM and *N* = 4 for IEM, independent experiments = 2). Non-significant statistical difference was indicated as n.s. (*p* > 0.05). **d** Scanning electron microscopic observation that shows the internal ultrastructure of GI tissue-derived ECM hydrogels (scale bars = 1 µm, independent experiment = 1). The data in (**c**) are presented as mean ± S.D. Statistical significance was analyzed using an unpaired, two-sided student’s *t*-test.

To develop a 3D matrix that better recapitulates the biochemical ECM composition of native GI tissues and ultimately replaces the use of Matrigel in organoid culture, we prepared ECM-based hydrogels derived from decellularized pig stomach and small intestinal tissues (Fig. 1a). First, we optimized decellularization protocols by adjusting the type (non-ionic versus ionic) and treatment time of detergents for decellularization (Supplementary Fig. 1). Using our optimized decellularization protocol (Protocol 1), which is based on a non-ionic detergent (Triton X-100)^17^, cellular components were completely removed from stomach and intestine tissues, whereas major ECM components (e.g., glycosaminoglycans; GAG) were preserved^17,18^ (Fig. 1b, c). Another decellularization protocol (Protocol 2) that uses ionic detergents (e.g., sodium deoxycholate; SDC) and has been widely used for cell removal^19,20^ effectively removed cellular components but impaired ECM preservation as indicated by a decrease in the GAG content (Supplementary Fig. 1a–c). Decellularized stomach-derived ECM (termed “SEM”) and decellularized intestine-derived ECM (termed “IEM”) were lyophilized, solubilized, and then induced to form 3D hydrogels at physiological pH and temperature via the kinetics of collagen fibril assembly (Supplementary Fig. 2). Both SEM and IEM hydrogels possessed a nanofibrous ultrastructure composed of interconnected ECM fibrils similar to collagen type I hydrogels (Fig. 1d)^20,21^. The hydrogels prepared with our decellularization protocol exhibited a higher elastic modulus (storage modulus measured at 1 Hz frequency) (1.6–3.3-fold) than those prepared using an ionic detergent (Supplementary Fig. 1d), indicating superior ECM preservation with our protocol. The storage modulus of hydrogel implies the ability of the hydrogel to store deformation energy, and the storage modulus is proportional to elastic energy^22^. In other words, higher storage modulus indicates higher elasticity and stronger mechanical property of hydrogel. Thus, ECM hydrogels with higher storage modulus may be more appropriate for the formation, growth, and long-term maintenance of organoids. As our optimized decellularization protocol allows a larger amount of ECM preservation and retains the stability of GI tissue-derived ECM hydrogels, the resultant hydrogels are more suitable for GI organoid culture.

We verified the safety of decellularized GI tissue-derived ECM hydrogels. First, we found that endotoxin levels of SEM and IEM were at 0.344 ± 0.007 EU/ml and 0.225 ± 0.016 EU/ml, respectively (Supplementary Fig. 3a). This may indicate that our protocol to decellularize GI tissues and prepare ECM hydrogels can exclude contamination of SEM and IEM hydrogels from pathogens in the GI tissues. Given that the endotoxin level acceptable for implantable medical devices is 0.5 EU/ml in the guideline of the Food and Drug Administration (FDA)^23^, our decellularized GI tissue-derived ECM hydrogels would be able to serve biomaterials with clinical feasibility. Next, we checked the immunogenicity of decellularized GI tissue-derived ECM hydrogels. Under the in vitro condition, the secretion of inflammatory cytokine tumor necrosis factor-α (TNF-α) from RAW 264.7 macrophages 3 and 6 h after exposure to SEM and IEM hydrogels was negligible, which was similar to that from cells with no treatment as a negative control (Supplementary Fig. 3b). Hematoxylin and eosin (H&E) staining of the tissue samples retrieved 1, 4, and 7 days after injection SEM and IEM hydrogels into subcutaneous space of mice revealed that neither tissue necrosis nor tissue damage was observed in the tissues injected with SEM and IEM hydrogels (Supplementary Fig. 3c). Toluidine staining also confirmed that excessive infiltration of inflammatory cells was not observed at the injection sites (Supplementary Fig. 3c). These results verified that decellularized GI tissue-derived ECM hydrogels are highly biocompatible and safe materials that can be considered for clinical applications. Decellularization of tissues allows manufacturing natural ECM-based materials with low immunogenicity by removing antigenic cellular components. Decellularized tissue-derived materials free from xenogeneic pathogens can be prepared through decellularization with a series of detergent treatments and sterilization processes (e.g., ethanol, ethylene oxide, ultraviolet (UV) irradiation, and supercritical carbon dioxide). Actually, Matrigel or Matrigel-derived products have never been approved by the FDA, while several commercialized products are based on decellularized tissue ECM (e.g., AlloDerm^®^, Meso BioMatrix^®^, and SynerGraft^®^, etc.) have been approved for clinical use by the FDA.

**Fig. 2:**
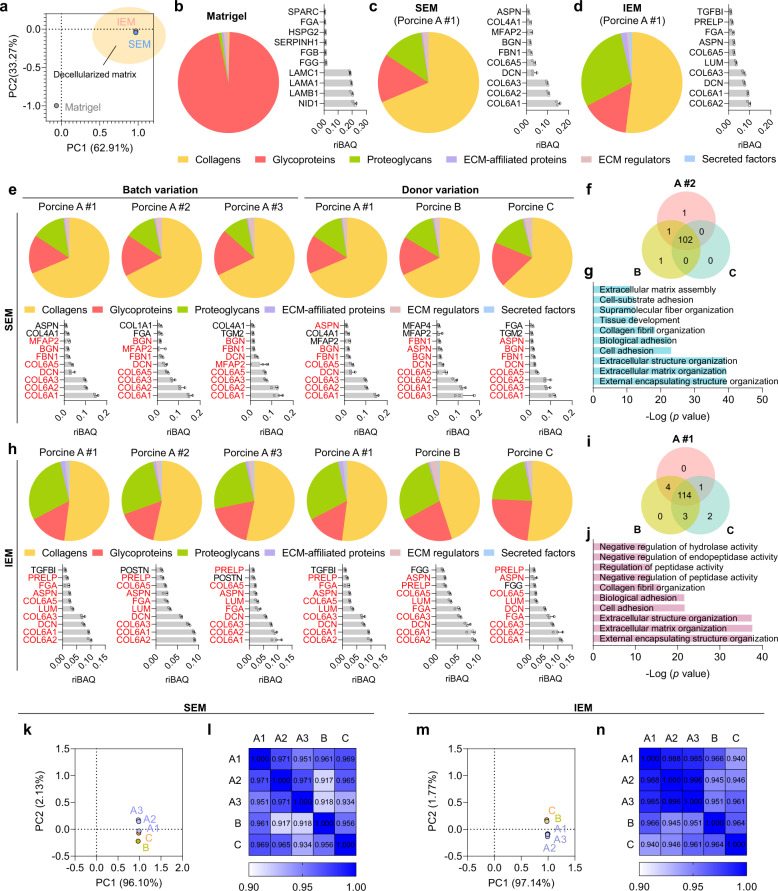
Proteomic analysis of GI tissue-derived ECM hydrogels. **a** Principal component analysis (PCA) of matrisome proteins present in Matrigel, SEM, and IEM. The composition of total matrisome proteins and the most abundant top 10 matrisome proteins in **b** Matrigel, **c** SEM, and **d** IEM (*N* = 4 for Matrigel and *N* = 3 for SEM and IEM). Comparison of the composition of total matrisome proteins and the top ten matrisome proteins with the highest expression in **e** SEM samples and **h** IEM samples derived from different tissue batches of the same donor (porcine A1–A3) or different donor batches (porcine A–C) (*N* = 3). Venn diagram showing the overlap of matrisome proteins in **f** SEM samples and **i** IEM samples derived from three different donors (porcine A–C). GOBP analysis of overlapped matrisome proteins in **g** SEM samples and **j** IEM samples from different donor batches. PCA analysis and Pearson’s correlation analysis to assess the similarity of total proteins detected in **k**, **l** SEM samples, and **m**, **n** IEM samples derived from different tissue batches of the same donor (porcine A1–A3) and different donor batches (porcine A–C). Pearson’s correlation coefficient values are indicated in each box. The averaged values from three biological replicates were used for analysis in (**f**), (**g**), (**i**), (**j**), and (**k**–**n**). The data in (**b**–**d**), (**e**), and (**h**) are presented as mean ± S.D.

Intensive proteomic analysis using mass spectrometry (MS) revealed the presence of stomach- and intestine-specific matrisome and non-matrisome components preserved in the SEM and IEM, which were distinct from Matrigel proteome components (Fig. 2a and Supplementary Fig. 4a, b). More core matrisome and matrisome-associated proteins were detected in SEM and IEM compared to Matrigel (Supplementary Fig. 4c). In total matrisome proteins, collagen subtypes and proteoglycans constituted about 67% and 13% in SEM and about 51% and 26% in IEM, respectively, whereas the portion of glycoproteins was about 17% in SEM and about 19% in IEM (Fig. 2c–e, h). In contrast, the majority of matrisome proteins in Matrigel were glycoproteins, which accounted for more than 96% of the total matrisome (Fig. 2b). Collagens are the most abundant structural elements of GI tissue ECM^24^, and proteoglycans play important roles in the regulation of signaling pathways involved in GI epithelial cell proliferation and tissue homeostasis^25–27^. Thus, SEM and IEM that contain a large quantity and variety of collagen subtypes and proteoglycans more closely reflect the in vivo GI microenvironment compared to Matrigel with only 0.4% collagen and 1% proteoglycans (Fig. 2b–d). Collagen Type VI (COL6A1, COL6A2, COL6A3, and COL6A5) and proteoglycans, such as decorin (DCN), were identified as the top 10 most abundant matrisome proteins in SEM and IEM, whereas glycoproteins, including laminin-111 (LAMA1, LAMB1, and LAMC1) and fibrinogen (FGA, FGB, and FGG), were the top 10 most abundant matrisome proteins in Matrigel (Fig. 2b–d).

Interestingly, the proteins significantly enriched in native stomach or intestine tissues were detected in much greater quantities in each tissue-specific ECM hydrogel compared to Matrigel (Supplementary Fig. 4d). Whereas Matrigel and IEM did not contain proteins known to be enriched in the native stomach, five non-matrisome proteins specific to the native stomach were identified only in SEM. Similarly, 5 matrisome proteins and 22 non-matrisome proteins enriched in the native intestine were detected only in IEM, verifying that IEM contains substantially more native intestinal proteins than other matrices. Although the exact roles of non-matrisome proteins have been relatively unexplored, several studies have demonstrated that non-matrisome proteins in some tissues are associated with cell growth and development^28,29^. Thus, we postulate that non-matrisome components in SEM and IEM hydrogels help the formation and development of GI organoids. In gene ontology biological process (GOBP) analysis of non-matrisome proteins in Matrigel, SEM, and IEM, the top ten representative GOBP terms indicated that the non-matrisome proteins of SEM and IEM were involved in cellular component biogenesis, cytoskeleton organization, and structural development, whereas those of Matrigel were more related to translation and RNA processing and metabolic process (Supplementary Fig. 5). Collectively, we speculate that SEM and IEM derived from decellularized GI tissues may serve as alternative matrices to Matrigel for GI organoid culture by better reconstituting the native GI-like microenvironment.

To evaluate the variation of decellularized GI tissue-derived matrices in the ECM compositions, we compared the samples under two conditions: (1) comparison of multiple sample batches derived from different locations in the same donor tissue (batch variation—porcine A1–A3), (2) comparison of multiple samples derived from different donor tissues (donor variation—porcine A–C). The compositions of matrisome proteins are quite similar in three sample batches of SEM or IEM derived from different locations in the same donor tissue (Fig. 2e, h). Interestingly, there was little variation in the profiles of matrisome proteins in three sample batches of SEM or IEM derived from different donor tissues (Fig. 2e, h). Moreover, the lists of the top 10 matrisome proteins with the highest expression almost overlapped between different batches from the same donor tissue or the tissue samples from different donors (eight proteins in SEM samples and nine proteins in IEM samples) (Fig. 2e, h). More than 100 matrisome proteins were overlapped in three sample batches of SEM or IEM derived from different donor tissues (SEM: 102 and IEM: 114) (Fig. 2f, i). GOBP analysis revealed that these overlapped matrisome proteins are mainly involved in the organization of ECM and extracellular structure (Fig. 2g, j). Principal component analysis (PCA) plot and heatmap of Pearson’s correlation coefficients confirmed that SEM and IEM samples derived from different tissue batches or different donor batches have high similarity in terms of protein compositions and profiles (Fig. 2k–n). Together, these proteomic analytical data demonstrate that decellularized GI tissue-derived matrices have a relatively low batch-to-batch variation in comparison to Matrigel.

To further investigate the variation in matrisome proteins and non-matrisome proteins in SEM and IEM samples, we compared the matrisome proteins and non-matrisome proteins detected in the SEM and IEM that are also expressed in native stomach and intestine tissues, respectively (Supplementary Fig. 6). Interestingly, among proteins highly enriched in the native stomach, one matrisome protein (ANXA10) and 4 non-matrisome proteins (CHIA, CLIC6, GHRL, PGC) were commonly detected in all batches of SEM samples, indicating that there is no significant variation in matrisome and non-matrisome proteins in the SEM from different tissue locations (porcine A1–A3) and donors (porcine A–C) (Supplementary Fig. 6a). In the case of proteins highly enriched in the native intestine, five matrisome proteins (ITLN2, LGALS2, LGALS4, MUC2, TINAG) were detected in all batches of IEM samples and one matrisome protein (REG4) was detected in two IEM sample batches (porcine A2, C). Total 19 non-matrisome proteins (ACTG2, ALDOB, AOC1, APOA4, ARHGAP45, CASP7, EPS8L2, FABP6, GSTA1, KLC4, KRT20, KRT8, LASP1, MYH14, OLFM4, RPS6KA1, SELENBP1, SMTN, and SRI) were commonly detected in all batches of IEM samples (Supplementary Fig. 6b). Other non-matrisome proteins GSDMB and JCHAIN were detected in four (porcine A2, A3, B, C) and three batches (porcine A2, B, C) of IEM samples, respectively. Two non-matrisome proteins (BCL2L15, HSD17B11) were detected in two IEM batches (porcine B, C). Therefore, most of matrisome proteins (5/6) and non-matrisome proteins (19/23) enriched in the native intestine were overlapped in all batches of IEM samples, demonstrating again that the variation in matrisome and non-matrisome proteins in the IEM is insignificant between tissue locations and donors. Moreover, these proteins commonly detected in different batches of SEM and IEM have involved in GI tissue-specific functions such as digestion, intestinal absorption, and gastric acid secretion (Supplementary Fig. 6c, d), indicating that GI tissue-derived ECM hydrogels can provide GI organoids with native GI-like microenvironments in a highly reproducible manner.

Additionally, we analyzed ECM compositions in decellularized tissues from different parts of the GI tract. We decellularized the esophagus, an upper part of the GI tract, and compared ECM profiles of decellularized esophagus-derived ECM (EEM) with those of SEM and IEM. The types and compositions of matrisome proteins in EEM were slightly different from those in SEM and IEM (Supplementary Fig. 7a). Three matrisome proteins (COL6A1, COL6A2, COL6A3) among the most abundant top 10 matrisome proteins in EEM were overlapped with those in SEM and IEM (Supplementary Fig. 7b). Despite such a slight discrepancy, PCA plot confirmed that overall ECM profiles in different parts of the GI tract (IEM, SEM, and EEM) were close each other compared to Matrigel with quite different ECM components (Supplementary Fig. 7c). Together, we conclude that ECM profiles and compositions in decellularized tissues are varying depending on the location in the GI tract, but ECM hydrogels derived from the GI tract have high similarity in comparison to materials from non-GI tract such as Matrigel.

### Utility of GI tissue-derived ECM hydrogels as a GI organoid culture matrix

To demonstrate the potential of decellularized GI tissue-derived ECM hydrogels (SEM and IEM) to substitute for Matrigel in organoid culture, we compared GI organoids generated in SEM and IEM hydrogels with those generated in Matrigel in the following four aspects: (i) organoid morphology, (ii) organoid formation efficiency, (iii) organ-specific gene expression level, and iv) organoid functionality. Before using the hydrogels in organoid culture, the rheological properties of SEM and IEM hydrogels were characterized at various concentrations to confirm their mechanical stability for organoid culture. Consistently higher storage modulus (G′) than the loss modulus (G″) in all tested concentrations indicated the stable formation of cross-linked ECM networks in the hydrogels (Supplementary Fig. 8a, b).

**Fig. 3:**
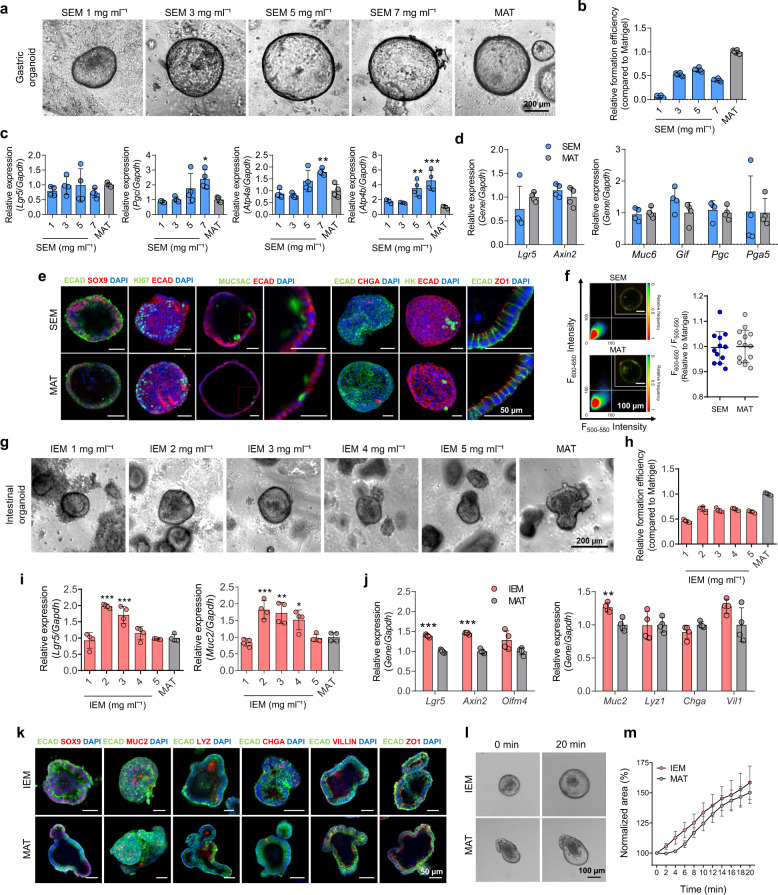
Optimization of GI tissue-derived ECM hydrogels for organoid culture as an alternative to Matrigel. **a** Brightfield images of gastric organoids grown in SEM hydrogels and Matrigel (MAT) at day 5 (scale bar = 200 µm, independent experiments = 3). **b** Quantification of gastric organoid formation efficiency in SEM hydrogels compared to in MAT (*N* = 6, independent experiments = 3). **c** qPCR analysis to compare mRNA expression in gastric organoids grown in each hydrogel (SEM 7 mg ml^−1^ versus MAT, *p = 0.0174 for *Pgc*, **p = 0.0051 for *Atp4a*, ***p < 0.0001 for *Atp4b*; SEM 5 mg ml^−1^ versus MAT, **p = 0.0029; *N* = 4, independent experiments = 3). **d** Comparison of mRNA expression in gastric organoids grown in 5 mg ml^−1^ SEM hydrogel and MAT (*N* = 4, independent experiments = 3). **e** Immunofluorescent staining for stemness markers (SOX9 and KI67), differentiation markers (MUC5AC, CHGA, and HK), a tight junction marker (ZO1), and a cell–cell adhesion/interaction marker (ECAD) in gastric organoids grown in 5 mg ml^−1^ SEM hydrogel and MAT (scale bars = 50 µm, independent experiments = 3). **f** Fluorescent staining with acridine orange for gastric organoids grown in 5 mg ml^−1^ SEM hydrogel and MAT (scale bars = 100 µm), and quantification of fluorescence (600–650 nm)/fluorescence (500–550 nm) from organoids in each hydrogel (*N* = 12 for SEM and *N* = 14 for MAT, independent experiments = 2). The color scale indicates the relative number of pixels displayed in the area. **g** Brightfield images of intestinal organoids grown in IEM hydrogels and MAT at day 6 (scale bar = 200 µm, independent experiments = 3). **h** Quantification of intestinal organoid formation efficiency in IEM hydrogels compared to in MAT (*N* = 4, independent experiments = 3). **i** qPCR analysis to compare mRNA expression of intestinal organoids within each hydrogel (IEM 2 mg ml^−1^ versus MAT, ***p < 0.0001 for *Lgr5*, ***p = 0.0009 for *Muc2*; IEM 3 mg ml^−1^ versus MAT, ***p = 0.0003 for *Lgr5*, **p = 0.0032 for *Muc2*; IEM 4 mg ml^−1^ versus MAT, *p = 0.0455 for *Muc2*; *N* = 4, independent experiments = 3). **j** Comparison of mRNA expression of intestinal organoids grown in 2 mg ml^−1^ IEM hydrogel and MAT (IEM versus MAT, ***p < 0.0001 for *Lgr5*, ***p < 0.0001 for *Axin2*, **p = 0.0035 for *Muc2*; *N* = 4, independent experiments = 3). **k** Immunofluorescent staining for a stemness marker, differentiation markers (MUC2, LYZ, CHGA, and VILLIN), a tight junction marker, and a cell–cell adhesion/interaction marker in intestinal organoids grown in 2 mg ml^−1^ IEM hydrogel and MAT (scale bars = 50 µm, independent experiments = 3). **l** Brightfield images of intestinal organoids grown in each hydrogel after forskolin treatment (scale bar = 100 µm), and **m** the area of forskolin-treated organoids normalized to the organoid area prior to forskolin treatment in each group (*N* = 4, independent experiments = 3). The data in **b**–**d**, **f**, **h**–**j**, and **m** are presented as mean ± S.D. Statistical significance was analyzed using one-way ANOVA with Tukey’s multiple comparisons test (**c**, **i**) and unpaired, two-sided student’s *t*-test (**d**, **j**).

First, we identified each optimal concentration of SEM and IEM hydrogels that effectively supported the development of GI organoids. When mouse gastric glands were seeded in SEM hydrogels at different ECM concentrations (1, 3, 5, 7 mg ml^−1^), gastric organoids formed in all tested concentrations (Fig. 3a), and the highest formation efficiency was observed at 5 mg ml^−1^ (Fig. 3b). Quantitative real-time polymerase chain reaction (qPCR) demonstrated that the gene expression of a stem cell marker (*Lgr5*) was similar in gastric organoids grown in SEM hydrogels at concentrations of 3 and 5 mg ml^−1^ and organoids grown in Matrigel. When we checked the expression levels of gastric epithelial cell markers (chief cell marker *Pgc* and parietal cell markers *Atp4a*, *Atp4b*) in the organoids grown in 5 mg ml^−1^ SEM hydrogels, the expression of *Pgc* and *Atp4a* was comparable to that of Matrigel organoids and the expression of *Atp4b* was higher in the organoids grown in 5 mg ml^−1^ SEM hydrogels than in the organoids in Matrigel (Fig. 3c). In general, stemness (*Lgr5* and *Axin2* for gastric stem cells) and gastric differentiation markers (*Muc6* for mucous neck cells, *Gif* for parietal cells, and *Pgc* and *Pga5* for chief cells) of gastric organoids grown in 5 mg ml^−1^ SEM hydrogel were expressed at similar levels to those grown in Matrigel (Fig. 3d). Considering gastric organoid formation efficiency and gene expression levels, we selected 5 mg ml^−1^ SEM hydrogel for further studies.

Similar experiments were conducted to optimize the concentration of IEM hydrogel used in intestinal organoid culture. All mouse intestinal crypts embedded in IEM hydrogels with 1–5 mg ml^−1^ concentrations developed into intestinal organoids (Fig. 3g), although the formation efficiency was slightly lower than that of Matrigel (Fig. 3h). The expression levels of stem cell marker (*Lgr5*) and goblet cell marker (*Muc2*) in intestinal organoids encapsulated in IEM hydrogels were similar to or higher than those embedded in Matrigel (Fig. 3i), and the highest expression was observed at 2 mg ml^−1^. All other intestinal differentiation markers (*Lyz1* for Paneth cells, *Chga* for enteroendocrine cells, and *Vil1* for enterocytes) of intestinal organoids grown in 2 mg ml^−1^ IEM hydrogel were expressed at comparable levels to those grown in Matrigel (Fig. 3j). Given these results, we selected 2 mg ml^−1^ IEM hydrogel for further studies in intestinal organoid culture. Although increased expression of stemness markers (*Lgr5*, *Axin2*) was observed in the intestinal organoids cultured in 2 mg ml^−1^ IEM hydrogel, as proliferation and differentiation may occur simultaneously at the early phase of intestinal organoid development, we speculate that differentiated cells may not be significantly less in intestinal organoids in IEM hydrogel compared to those in Matrigel. Actually, a previous study using decellularized matrices for intestinal organoid culture also showed a simultaneous increase in both stem cell markers and differentiated cell markers in the organoids grown in ECM hydrogel^20^, which is consistent with our current finding. Overall, GI tissue-specific stemness and differentiation signatures of the organoids in GI tissue-derived ECM hydrogels seem to be comparable to those of the organoids in Matrigel.

We further compared phenotypes and functions of organoids produced in optimized ECM hydrogels with those of organoids grown in Matrigel. Gastric organoids in SEM hydrogel (5 mg ml^−1^) grew comparably to organoids in Matrigel in size and morphology (Supplementary Fig. 9a). Although the projected area of gastric organoids grown in SEM hydrogel was slightly smaller than that of organoids grown in Matrigel, the variation of size distribution and morphology was lower in SEM organoids compared to Matrigel organoids (Supplementary Fig. 9c, e). The coefficient of variation (CV) in size of gastric organoids was 66.7% in SEM hydrogel and 74.5% in Matrigel. Moreover, gastric organoids in both matrices underwent similar organotypic development and contained heterogeneous populations of KI67-positive proliferative cells and MUC5AC-, HK-, and CHGA-positive gastric cells (Fig. 3e). The areas stained positively for proliferation marker (SOX9) and major differentiation marker (MUC5AC) in the gastric organoids grown in SEM hydrogel were not statistically different from those in the organoids cultured in Matrigel (Supplementary Fig. 10a). These data indicate that the development of cellular compositions in the gastric organoids cultured in SEM hydrogel is generally similar to that in the Matrigel organoids.

The generation of functional parietal cells, one of the most important cells in the function of the stomach, is still challenging in the current culture system of gastric organoids. This limitation of the current gastric organoid culture system may be solved through recapitulation of gastric microenvironments. For example, a previous study demonstrated the co-culture of fundic organoids with stomach mesenchymal cells to maintain the function of parietal cells in gastric organoids^30^. In our case, fundic organoids containing parietal cells could be developed in our gastric organoid populations because we used whole stomach tissues including fundus region for the generation of gastric organoids. The presence of parietal cells in gastric organoids was confirmed by the expression of parietal cell marker genes (*Atp4a*, *Atp4b*) and protein (HK) (Fig. 3c, e). We compared the gastric function of organoids grown in SEM hydrogel and Matrigel by evaluating acid secretion using acridine orange dye. The degree of acid accumulation in organoids was similar in both groups (Fig. 3f). We also compared the gene expression of three major parietal cell markers (*Gif*, *Atp4a*, and *Atp4b*) in gastric organoids cultured in SEM hydrogel with that of stomach and intestine tissues (Supplementary Fig. 11a). RNA sequencing analysis revealed that the expression of those parietal cell markers in SEM organoids was significantly higher than that in intestine tissue, while significantly lower than in stomach tissue. This result suggests that gastric organoids cultured in SEM hydrogel contain parietal cells, but lacks parietal cell differentiation in comparison to native stomach tissue. We examined the cellular ultrastructure of gastric organoids cultured in SEM hydrogel using serial-section scanning electron microscopy to clarify the presence of parietal cells in gastric organoids (Supplementary Fig. 11b). Gastric parietal cells are characterized by a large number of mitochondria and intracellular canaliculi associated with acid secretion^31^. We found that some cells in the gastric organoids grown in SEM hydrogel have such characteristic ultrastructure of parietal cells, which directly confirmed the existence of gastric parietal cells in SEM organoids. The function of parietal cells in gastric organoids cultured in SEM hydrogel was also validated by observing acid secretion from gastric organoids treated with histamine (Supplementary Fig. 11c). However, the portion of parietal cells is much less in SEM organoids than in gastric gland tissue, and thus additional improvements would be required to further enrich parietal cells critical for the functionality of gastric organoids.

Likewise, intestinal crypts seeded in IEM hydrogel at the optimized concentration (2 mg ml^−1^) developed into intestinal organoids with similar shape and size as observed in Matrigel (Supplementary Fig. 9b). The variation of size distribution was lower in IEM organoids than in Matrigel organoids (CV: 35.1% in IEM hydrogel and 47.7% in Matrigel) (Supplementary Fig. 9d). The variation in morphology of intestinal organoids looks less in IEM hydrogel compared to Matrigel (Supplementary Fig. 9f). Additionally, intestinal organoids grown in IEM hydrogel retained intestinal epithelial cells, including absorptive cells and three types of secretory cells, in a similar manner as intestinal organoids grown in Matrigel (Fig. 3k). The areas stained positively for major differentiation marker proteins (MUC2, LYZ) in the intestinal organoids grown in IEM hydrogel were not significantly different from those in the organoids cultured in Matrigel (Supplementary Fig. 10b). We also compared the cystic fibrosis conductance regulator (CFTR) function of intestinal organoids cultured in IEM hydrogel and Matrigel using a forskolin-induced swelling assay (Fig. 3l, m). Intestinal organoids from both groups rapidly enlarged at similar rates upon forskolin treatment, suggesting that intestinal organoids produced in IEM hydrogel also possess the ability to regulate luminal fluid secretion. Taken together, our results confirm the utility of SEM and IEM hydrogels as GI organoid culture platforms that enable the production and differentiation of GI organoids with structural and functional features comparable to organoids grown in Matrigel.

As mucus secretion is one of the key functions in GI tissues, generation of mucus layer is required for studying GI physiology in disease models and interactions between host and gut microbiota^32^. MUC5AC and MUC2 immunostaining confirmed mucus secretion into the lumen of GI organoids cultured in SEM and IEM hydrogels (Fig. 3e, k), but the mucus layer covering the organoids was not observed due to an insufficient number of goblet cells. Previous studies reported several methods to generate a mucus layer in GI organoids by promoting differentiation of goblet cells through depriving culture medium of Wnt3A and R-spondin1 proteins or using culture devices such as microfluidic chip^33,34^. Thus, a combination of GI tissue-derived ECM hydrogels with additional strategies of adjusting signaling factors or culture conditions would be required to produce functional GI organoids covered by a mucus layer.

To check the differential effect of subepithelial ECM and mucus ECM on organoid development, we prepared ECM hydrogels derived from decellularized gastric mucosa and intestinal mucosa, and compared mechanical properties and organoid formation capacity of decellularized GI mucosa-derived ECM hydrogels with those of ECM hydrogels prepared from decellularized whole stomach and intestine containing subepithelial ECM. When prepared at the same concentrations (5 mg ml^−1^ SEM and 2 mg ml^−1^ IEM), the elastic modulus of SEM and IEM derived from decellularized mucosal layers was significantly lower than that of SEM and IEM from decellularized whole tissues (Supplementary Fig. 12a–d). Due to poor physical and mechanical properties, mucosa-derived SEM and IEM did not support GI organoid culture. As they could not sustain the developing GI organoids, the organoids often grew attached to the bottom of the culture plate and underwent morphological deformation (Supplementary Fig. 12e, g). In particular, intestinal organoids did not retain their spherical shapes in the mucosa-derived IEM hydrogel. Consequently, the GI organoid formation efficiency was significantly lower in the mucosa-derived SEM and IEM hydrogels than in the whole tissue-derived SEM and IEM hydrogels (Supplementary Fig. 12f, h). These data demonstrate that ECM hydrogels prepared with whole GI tissues can serve as more effective culture matrices likely due to more abundant subepithelial ECM components than ECM hydrogels derived from other parts of GI tissues without subepithelial ECM.

### Transcriptome profiles of GI organoids generated in GI tissue-derived ECM hydrogels

**Fig. 4:**
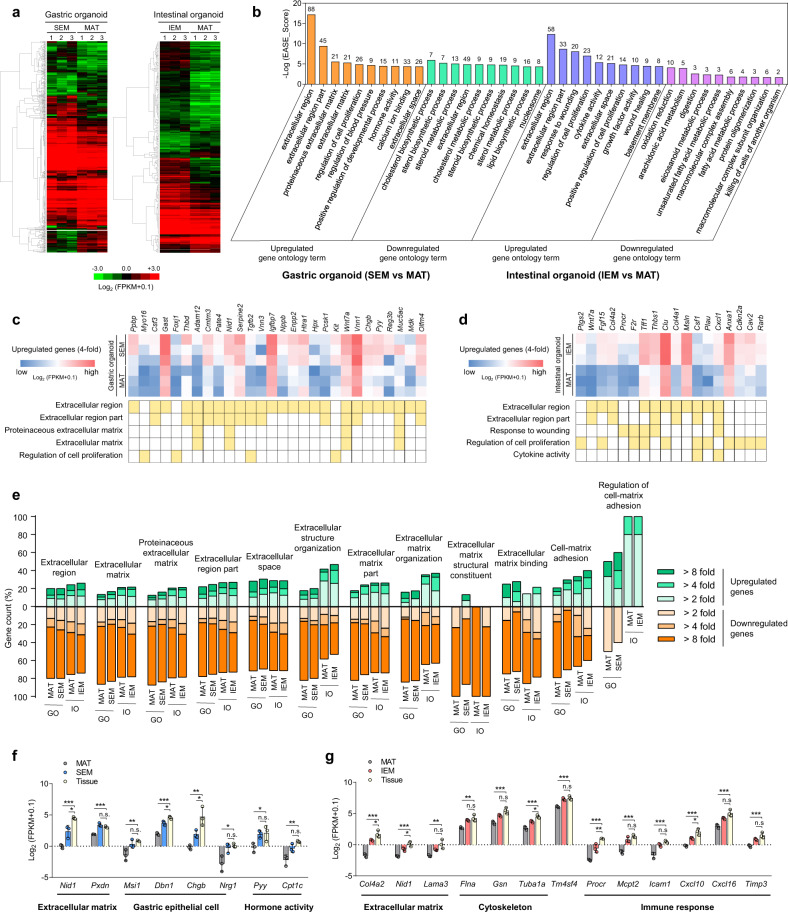
Transcriptomic profiles of GI organoids cultured in GI tissue-derived ECM hydrogels. **a** Hierarchical clustering heatmap of differentially expressed genes (DEGs) in GI organoids cultured in SEM and IEM hydrogels compared to those cultured in Matrigel (MAT). DEGs were filtered by fold-change > 2, *p* < 0.05, and FDR < 0.1 (*N* = 3). **b** Top 10 gene ontology (GO) terms that were upregulated and downregulated in GI tissue-derived ECM hydrogel groups compared to MAT group (fold-change > 2, *p* < 0.05, FDR < 0.1). Gene counts are indicated above each column. Heatmaps comparing the expression of individual genes that were upregulated more than 4-fold in **c** SEM and **d** IEM hydrogel groups compared to the MAT group. The GO terms for each gene are represented in yellow (*p* < 0.05, FDR < 0.1). **e** The percentage of DEGs belonging to ECM-related GO terms in GI ECM organoids or Matrigel organoids compared to native GI tissue. DEGs with 2-, 4-, and 8-fold changes are indicated by the color gradient intensity (*p* < 0.05, FDR < 0.1). **f**, **g** Comparison of expression values (Log_2_ [FPKM + 0.1]; FPKM, fragments per kilobase of transcript per million mapped reads) of selected genes involved in **f** gastric or **g** intestinal development and homeostasis in native GI tissues and in GI organoids cultured in GI tissue-derived ECM hydrogels or Matrigel [**f** MAT versus Tissue, ***p = 0.0002 for *Nid1*, ***p = 0.0005 for *Pxdn*, **p = 0.0097 for *Msi1*, ***p < 0.0001 for *Dbn1*, **p = 0.0019 for *Chgb*, *p = 0.0147 for *Nrg1*, *p = 0.0404 for *Pyy*, **p = 0.0028 for *Cpt1c*; SEM versus Tissue, *p = 0.0116 for *Nid1*, *p = 0.0132 for *Dbn1*, *p = 0.0274 for *Chgb*; *N* = 3] and [**g** MAT versus Tissue, ***p < 0.0001 for *Col4a2*, ***p = 0.0003 for *Nid1*, **p = 0.0041 for *Lama3*, **p = 0.0021 for *Flna*, ***p = 0.0009 for *Gsn*, ***p = 0.0002 for *Tuba1a*, ***p = 0.0006 for *Tm4sf4*, ***p < 0.0001 for *Procr*, ***p = 0.0002 for *Mcpt2*, ***p = 0.0004 for *Icam1*, ***p = 0.0003 for *Cxcl10*, ***p = 0.0009 for *Cxcl16*, ***p = 0.0009 for *Timp3*; IEM versus Tissue, *p = 0.0356 for *Col4a2*, *p = 0.0177 for *Nid1*, *p = 0.0152 for *Tuba1a*, **p = 0.0067 for *Procr*, *p = 0.0176 for *Cxcl10*; *N* = 3]. Non-significant statistical difference was indicated as n.s. (*p* > 0.05). The data in (**f**, **g**) are presented as mean ± S.D. Statistical significance was analyzed using one-way ANOVA with Tukey’s multiple comparisons test (**f**, **g**).

To characterize the GI organoids produced in ECM hydrogels at a transcriptomic level, we performed an RNA-sequencing analysis of GI organoids grown in GI tissue-derived ECM hydrogels in comparison to GI organoids grown in conventional Matrigel and to native GI tissues. Many differentially expressed genes (DEGs) were identified between the organoids grown in SEM and IEM hydrogels and those grown in Matrigel (Fig. 4a). Using a false-discovery rate (FDR) cutoff of <0.1 and a fold-change > 2, we identified 590 DEGs (312 upregulated genes and 278 downregulated genes) and 270 DEGs (213 upregulated genes and 57 downregulated genes) in SEM hydrogel-cultured gastric organoids and IEM hydrogel-cultured intestinal organoids, respectively. Gene ontology (GO) terms related to ECM and cell proliferation were significantly upregulated in both gastric and intestinal organoids grown in each tissue-specific hydrogel (SEM or IEM) (Fig. 4b). In comparison to Matrigel organoids, genes related to positive regulation of the developmental process, hormone activity, and calcium ion binding were also upregulated in SEM organoids, and genes related to response to wounding and cytokine activity were upregulated in IEM organoids. When the selection criterion was strengthened to genes that were upregulated at least fourfold in GI tissue-derived hydrogel groups compared to Matrigel group, most of the selected genes were involved in the extracellular region, the ECM, or the regulation of cell proliferation (Fig. 4c, d and Supplementary Fig. 13). For the GO categories related to the extracellular environment (e.g., extracellular structure organization and ECM binding) and cell–matrix interaction (e.g., cell–matrix adhesion), we compared the gene expression of GI organoids cultured in SEM or IEM with that of native GI tissues (Fig. 4e). Although many genes were downregulated in organoids compared to native tissues, the number and level of upregulated genes were much higher in organoids cultured in SEM or IEM hydrogels than in those grown in Matrigel. As the expression of ECM-related genes plays an important role in ECM remodeling and organization, which are critical for cell differentiation and organ regeneration^35,36^, our results suggest that GI tissue-specific hydrogels provide added benefit to GI organoid development compared to Matrigel. Based on the RNA sequencing data, we compared the expression of enteroendocrine cell subtype markers in intestinal organoids cultured in IEM hydrogel and Matrigel (Supplementary Fig. 14). Enteroendocrine cells regulate intestinal activities by producing hormones, and their subtypes are classified according to their hormone products: L cells (*Gcg, Pyy*), I cells (*Cck*), K cells (*Gip*), N cells (*Nts*), S cells (*Sct*), EC cells (*Tph1*), X cells (*Ghrl*), G cells (*Gast*), and D cells (*Sst*)^37,38^. The expression levels of enteroendocrine cell subtype markers were overall similar between IEM organoids and Matrigel organoids, except for *Gip* and *Gast* with higher expression in IEM organoids and *Ghrl* with higher expression in Matrigel organoids. These results may verify that IEM hydrogel could generate intestinal organoids containing enteroendocrine cells responsible for hormone production, which would be suitable for preclinical drug screening and translational applications.

Given that GI tissue-derived ECM hydrogels are collagen-rich matrices and collagen culture condition is known to activate Yes-associated protein (YAP) signaling^39^, we examined YAP signaling in the organoids cultured in IEM hydrogel and Matrigel. Immunocytochemical staining to show YAP expression and distribution in the intestinal organoids and quantification of the nuclear/cytoplasmic ratio of YAP signals revealed that YAP nuclear/cytoplasmic ratio was higher in the intestinal organoids cultured in IEM hydrogel than in the organoids cultured in Matrigel (Supplementary Fig. 15a, b), indicating YAP dephosphorylation and activation under IEM condition. RNA sequencing analysis showed that the significantly higher expression of several YAP target genes, such as *Ctgf, Ankrd1, Ereg, Cyr61, Anxa1*, and *Ly6a*, was observed in the IEM organoids than in the Matrigel organoids (Supplementary Fig. 15c, d). Organoid culture in 3D collagen type I hydrogel was previously reported to induce YAP/TAZ activation in intestinal organoids and conversion of cell fate to fetal-like states with regenerative capacity^39,40^. Yui et al. demonstrated that Wnt/collagen-based culture condition allows intestinal organoids to have genetic profiles similar to those observed during tissue regeneration. Therefore, our data from YAP analysis may suggest that IEM hydrogel comprising abundant collagens is more effective for YAP signaling activation than Matrigel and confers regenerative epithelium-like properties to intestinal organoids.

Importantly, GI organoids cultured in SEM and IEM hydrogels exhibited expression of several important genes related to GI epithelial homeostasis that more closely resembled the expression profiles of the native tissues than did organoids are grown in Matrigel (Fig. 4f, g). The expression of specific genes encoding core matrisome proteins (*Pxdn* and *Nid1*) was similar or slightly lower in gastric organoids grown in SEM hydrogel compared to the native stomach (Fig. 4f). In SEM organoids, the stomach epithelial stem cell marker *Msi1*, the acid-secreting parietal cell marker *Dbn1*, and the enteroendocrine cell marker *Chgb* were expressed at levels similar to the native stomach tissues. Neuregulin-1 (*Nrg1*) is known to promote stem cell proliferation and epithelial regeneration^41^ and was expressed in SEM organoids at levels as high as those in native stomach tissues. Both SEM organoids and native stomach tissues expressed *Nrg1* at significantly higher levels than Matrigel organoids. The expression of genes involved in hormone activity, such as the gastric hormone gene *Pyy* and the ghrelin signaling pathway-related gene *Cpt1c*, was also similar in SEM organoids and native stomach tissues. In intestinal organoids grown in IEM hydrogel, we found that the expression levels of core matrisome protein-encoding genes (*Col4a2, Nid1*, and *Lama3*), cytoskeleton-related genes (*Flna, Gsn*, and *Tuba1a*), and an intestinal epithelial gene involved in thiamin uptake (*Tm4sf4*) were comparable to those in native intestinal tissues (Fig. 4g). Compared with Matrigel organoids, IEM organoids showed significantly increased expression of genes related to wound healing, inflammation, and immune response *(Procr*, *Mcpt2*, *Icam1*, *Cxcl10*, *Cxcl16*, and *Timp3*), which were expressed at levels similar to those of native intestinal tissues. The expression of these genes at levels similar to the tissue may help to maintain intestinal barrier homeostasis under physiological conditions^42,43^. Therefore, organoid culture using IEM hydrogel may preserve the intrinsic ability of intestinal organoids to cope with injury and to maintain tissue homeostasis. Overall, our results demonstrate that GI tissue-derived ECM hydrogels contribute to producing more mature and functional GI organoids in comparison to conventional Matrigel and enable the generation of GI organoids which recapitulate the cellular compositions and functional properties of adult GI tissues.

### Tissue-specific and age-related effects of GI tissue-derived ECM hydrogel on GI organoid development

**Fig. 5:**
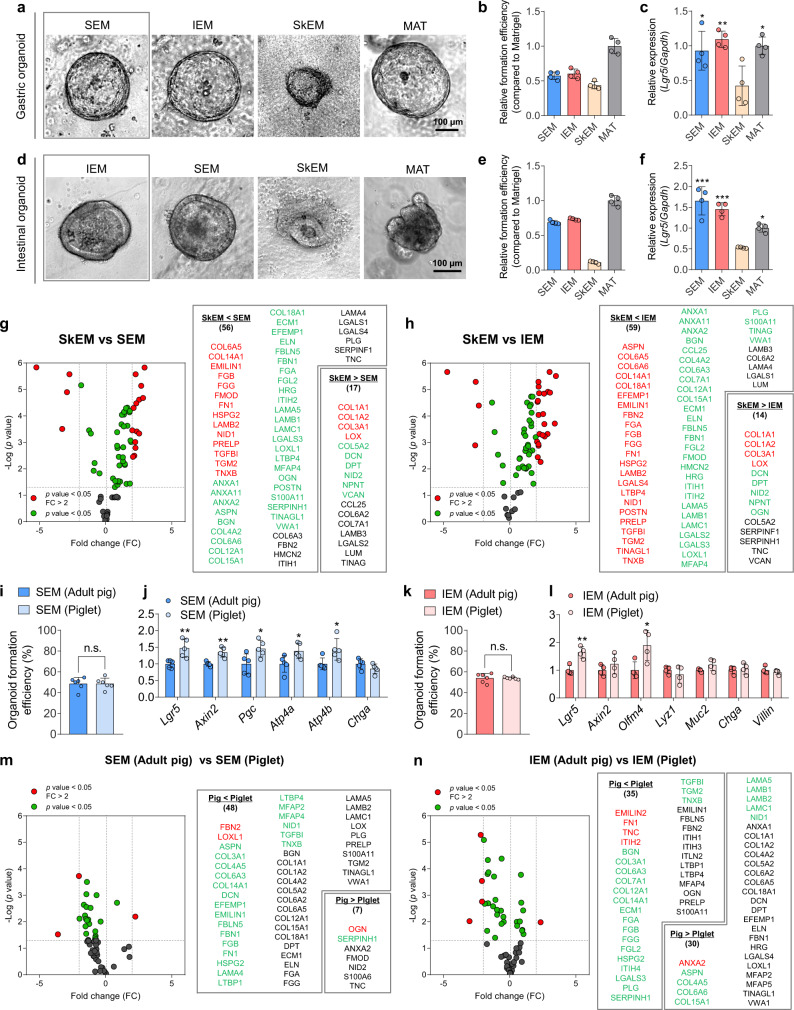
Tissue-specific and age-related effects of GI tissue-derived ECM hydrogels in GI organoid culture. **a** Brightfield images and **b** formation efficiency of gastric organoids cultured in decellularized tissue-derived ECM hydrogels from stomach, intestine, and skin tissues relative to gastric organoids cultured in Matrigel (MAT) at day 5 (*N* = 4, scale bar = 100 µm). **c** Comparison of mRNA expression (*Lgr5*) in gastric organoids in each hydrogel (SEM versus SkEM, *p = 0.0289; IEM versus SkEM, **p = 0.0045; MAT versus SkEM, *p = 0.0129; *N* = 4, independent experiments = 2). **d** Brightfield images and **e** formation efficiency of intestinal organoids cultured in decellularized tissue-derived ECM hydrogels from stomach, intestine, and skin tissues relative to intestinal organoids cultured in MAT at day 6 (*N* = 4, scale bar = 100 µm). **f** Comparison of mRNA expression (*Lgr5*) in intestinal organoids in each hydrogel (SEM versus SkEM, ***p < 0.0001; IEM versus SkEM, ***p = 0.0001; MAT versus SkEM, *p = 0.0219; *N* = 4, independent experiments = 3). Proteomic analysis of the relative quantity of matrisome proteins contained in **g** SEM and **h** IEM compared to SkEM. Comparison of **i** the formation efficiency (*N* = 6) and **j** gastric marker gene expression of organoids cultured in SEM hydrogels derived from two-month-old piglets (Piglet) and 6-month-old adult pigs (Adult pig) [SEM (Adult pig) versus SEM (Piglet), **p = 0.0076 for *Lgr5*, **p = 0.0013 for *Axin2*, *p = 0.0304 for *Pgc*, *p = 0.0345 for *Atp4a*, *p = 0.0299 for *Atp4b*; *N* = 5]. Comparison of **k** the formation efficiency (*N* = 6) and **l** intestinal marker gene expression of intestinal organoids cultured in IEM hydrogels derived from 2-month-old piglets (Piglet) and 6-month-old adult pigs (Adult pig) [IEM (Adult pig) versus IEM (Piglet), **p = 0.0029 for *Lgr5*, *p = 0.0282 for *Olfm4*; *N* = 4]. Proteomic analysis of the relative quantity of matrisome proteins contained in **m** SEM and **n** IEM derived from 2-month-old piglets and 6-month-old adult pigs. Non-significant statistical difference was indicated as n.s. (*p* > 0.05). The data in **b**, **c**, **e**, **f**, **i**–**l** are presented as mean ± S.D. Statistical significance was analyzed using one-way ANOVA with Tukey’s multiple comparisons test (**c**, **f**) and unpaired, two-sided student’s *t*-test (**g**–**n**).

To test the hypothesis that stomach- or intestine-specific ECM cues present in SEM or IEM hydrogels provide favorable in vivo-like microenvironments for GI organoid development, we explored the tissue-specific effects of ECM hydrogels in organoid culture. Decellularized tissue-derived ECM hydrogels were prepared from six tissue types (stomach, intestine, skin, lymph, heart, and muscle) and were tested as culture matrices for GI organoids (Fig. 5a–f and Supplementary Fig. 16). Live/Dead staining indicated that the viability of GI organoids was relatively lower in non-GI tissue ECM hydrogels (*i.e*., skin, lymph, heart, and muscle) than in GI ECM hydrogels (SEM and IEM) (Supplementary Fig. 16). Most cells were highly viable in GI organoids cultured in SEM and IEM hydrogels. In contrast, a larger number of dead cells was observed in GI organoids cultured in ECM hydrogels derived from decellularized skin, lymph, heart, and muscle tissues. This result clearly indicates that GI tissue-derived ECM hydrogels can provide the most favorable microenvironments for the viability and growth of GI organoids. Interestingly, gastric and intestinal organoids developed well in IEM and SEM hydrogels, respectively. The size and morphology of organoids and organoid formation efficiency were not substantially different even when they were cultured in their counterpart ECM hydrogels (Fig. 5a, b, d, e, and Supplementary Fig. 16). Combined with our observation that many important matrisome proteins were commonly present in both SEM and IEM hydrogels (Fig. 2c–e, h and Supplementary Fig. 4c), these results suggest that SEM and IEM provide quite equivalent ECM environments for GI organoids. In contrast, other types of tissue-derived ECM hydrogels (skin, lymph, heart, and muscle) did not support the development of GI organoids, and the structure of the formed organoids was immature (Supplementary Fig. 16). For example, GI organoids cultured in decellularized skin-derived ECM (SkEM) hydrogel exhibited irregular and disorganized morphology, and the generated organoids were much smaller in size and less proliferative than those grown in GI ECM hydrogels (Fig. 5a, d). Additionally, the organoid formation efficiency in SkEM hydrogels was lower than in SEM or IEM hydrogels (Fig. 5b, e), and the expression of a stemness marker was significantly lower in SkEM hydrogel organoids (Fig. 5c, f). The relative proteomic quantitation between SkEM and GI-tissue matrisomes (Fig. 5g, h) clearly demonstrated the tissue-specific effects of GI ECM hydrogels on organoid development. The quantity of the 56–59 core matrisome proteins that are critical for GI development and function was much greater in SEM and IEM hydrogels than in SkEM hydrogels. For example, the level of collagen type VI (COL6A3, COL6A5, and COL6A6) was higher in GI tissue-derived ECM than in SkEM. Collagen type VI is a major component of the mechanical microenvironment of GI tissue that regulates epithelial cell-fibronectin assembly^44,45^. Although some glycoproteins, such as fibronectin (FN1) and laminin (LAMA5, LAMB1, LAMB2, and LAMC1), were detected in SkEM, the amounts were lower than those in SEM and IEM. These glycoproteins are known to be essential for GI epithelial cell proliferation and differentiation^36,46–48^. Collectively, these results validate the importance of the tissue origin of ECM hydrogels in organoid culture.

Next, we investigated the age-related effects of tissue ECM on GI organoid culture. ECM remodeling constantly occurs throughout the lifetime of an organism, and changes in ECM composition and properties during aging affect cell proliferation and differentiation^49^. Thus, we compared the performance of SEM and IEM hydrogels derived from pigs of different ages (2-month-old piglet weighing approximately 20 kg versus 6-month-old adult pig weighing approximately 100–120 kg) for GI organoid culture. When we checked organoid formation in adult pig-derived ECM hydrogels and piglet-derived ECM hydrogels, there was no significant difference in the organoid formation efficiency between each ECM hydrogel for both gastric and intestinal organoids (Fig. 5i, k). Interestingly, the expression of stem cell markers (*Lgr5*, *Axin2*, and *Olfm4*) was higher in GI organoids grown in piglet-derived ECM hydrogels than in organoids grown in adult pig-derived ECM hydrogels (Fig. 5j, l). To determine which components in piglet and adult pig ECM affected the differential gene expression profiles of GI organoids, we performed a proteomic analysis of the matrisome in each condition (Fig. 5m, n). When compared with adult pig SEM and IEM, the quantities of 48 components [e.g., fibrillin2 (FBN2)] and 35 components [e.g., fibronectin 1 (FN1) and tenascin C (TNC)] were greater in piglet SEM and IEM, respectively (Fig. 5m, n). Fibrillin2 and tenascin C increase epithelial proliferation^50^, and fibronectin is important for stem cell survival^36^ and tissue regeneration^51^. Therefore, we inferred that these proteins, which are abundant in the piglet matrisome, support the growth potential of GI organoids. These findings provide important clues regarding the factors that should be considered to produce high-quality organoids and to design ECM-based hydrogels for organoid culture.

Additionally, we verified whether highly upregulated ECM proteins in piglet IEM hydrogel (FN and TNC) are really responsible for the better ability of piglet IEM to support intestinal organoid culture. We investigated whether the addition of FN and TNC into the adult-pig IEM hydrogel improves the quality of intestinal organoids. Interestingly, the addition of FN alone into adult-pig IEM hydrogel enhanced the expression of stem cell markers (*Lgr5* and *Axin2*) in intestinal organoids, whereas the addition of TNC alone or both FN and TNC did not increase stem cell marker expression (Supplementary Fig. 17). These results demonstrate that FN may be a more crucial factor involved in the improved ability of piglet ECM for supporting organoid culture. Here, we tested only two proteins of piglet ECM and limited concentrations (25 and 50 μg ml^−1^), but future studies to systemically identify more potent ECM molecules and optimal concentrations would be able to facilitate the development of chemically defined hydrogels for GI organoid culture.

### The practicality of GI tissue ECM hydrogels for organoid culture and transplantation

**Fig. 6:**
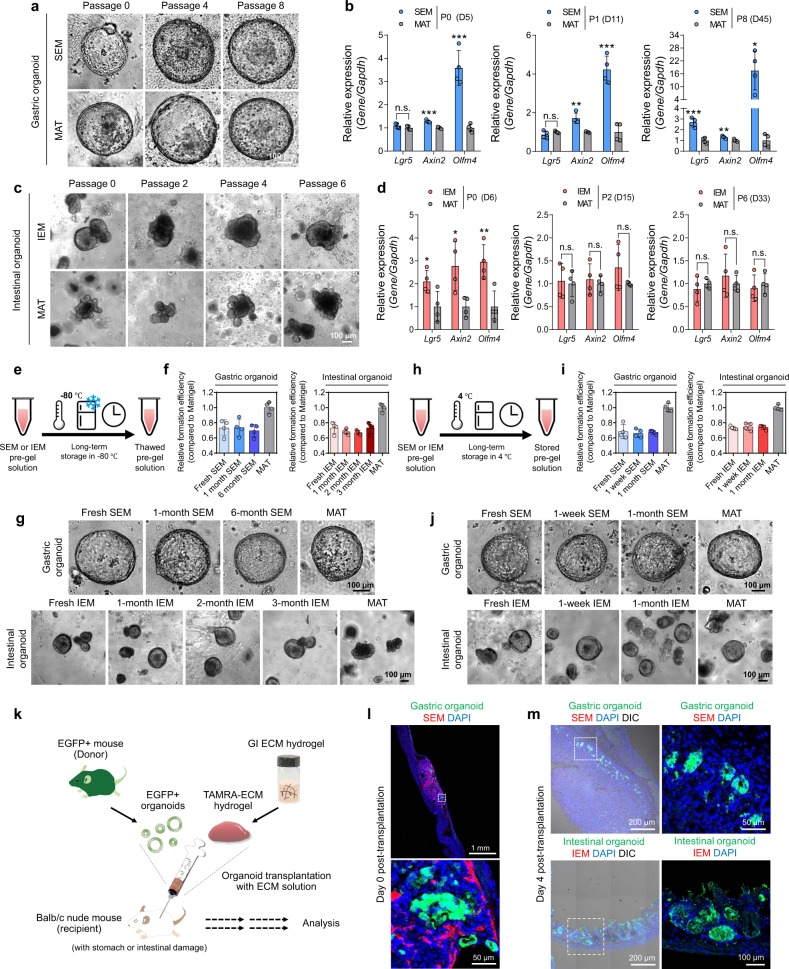
Versatile utility and practical applicability of GI tissue-derived ECM hydrogels. **a** Brightfield images of gastric organoids cultured in SEM hydrogel and Matrigel (MAT) at passage 0, 4, and 8 (scale bar = 100 µm). **b** Comparison of mRNA expression (*Lgr5*, *Axin2*, and *Olfm4*) in gastric organoids grown in SEM hydrogel and MAT at passage 0 (day 5), 1 (day 11), and 8 (day 45) [(P0) SEM versus MAT, ***p = 0.0002 for *Axin2*, ***p = 0.0005 for *Olfm4*; (P1) SEM versus MAT, **p = 0.0019 for *Axin2*, ***p = 0.0003 for *Olfm4*; (P8) SEM versus MAT, ***p = 0.0004 for *Lgr5*, **p = 0.0095 for *Axin2*, *p = 0.0107 for *Olfm4*; *N* = 4, independent experiments = 2]. **c** Brightfield images of intestinal organoids cultured in IEM hydrogel and MAT at passage 0, 2, 4, and 6 (scale bar = 100 µm). **d** Comparison of mRNA expression (*Lgr5*, *Axin2*, and *Olfm4*) in intestinal organoids grown in IEM hydrogel and MAT at passage 0 (day 6), 2 (day 15), and 6 (day 33) [(P0) IEM versus MAT, *p = 0.0380 for *Lgr5*, *p = 0.0214 for *Axin2*, **p = 0.0087 for *Olfm4*; *N* = 4, independent experiments = 2]. **e**, **h** Schematic illustration of GI tissue-derived ECM hydrogels used for organoid culture after long-term storage at −80 °C (for 1–6 months) or 4 °C (for 1–4 weeks). **f**, **i** The formation efficiency (*N* = 4) and **g**, **j** microscopic observation of GI organoids in GI tissue-derived ECM hydrogels thawed after long-term storage at −80 °C or 4 °C (scale bars = 100 µm). **k** Schematic illustration of EGFP^+^ GI organoid transplantation with tetramethylrhodamine (TAMRA)-labeled ECM hydrogel into mouse models with acute epithelial injury of the stomach and intestine. Fluorescence images to check the engraftment of EGFP^+^ GI organoids in the injured tissues **l** on the day of transplantation and **m** 4 days after transplantation (scale bars = 200 µm). Representative images obtained with the samples from two independent experiments (two mice per group) are shown in (**l**, **m**). Non-significant statistical difference was indicated as n.s. (*p* > 0.05). The data in **b**, **d**, **f**, and **i** are presented as mean ± S.D. Statistical significance was analyzed using unpaired, two-sided student’s *t*-test (**b**, **d**).

So far, we have shown that GI tissue-derived ECM hydrogels have highly desirable features. Next, we sought to determine the practical applicability of these hydrogels. First, we evaluated the long-term expansion and passage of GI organoids in SEM and IEM hydrogels compared to those in Matrigel. The subculture of GI organoids in SEM and IEM hydrogels enabled stable growth and expansion of organoids persistent up to more than passages 6–8 (Fig. 6a, c). Overall, we did not observe any difference in the turnover rate and splitting ratio of passage between the organoids grown in ECM hydrogels and Matrigel. qPCR analysis of stemness markers (*Lgr5*, *Axin2*, and *Olfm4*) indicated that GI organoids maintained self-renewal ability after long-term expansion (days 33–45) in GI tissue-derived ECM hydrogels at comparable levels to organoids subcultured in Matrigel (Fig. 6b, d). The colon organoids-derived mouse large intestine also grew well in IEM hydrogel as similar to Matrigel. We assume that IEM could be widely used for culturing other types of GI tissue organoids with similarity to small intestinal organoids (e.g., colon organoids) (Supplementary Fig. 18a). SEM and IEM hydrogels not only supported the growth of primary tissue-derived GI organoids but also supported the growth of human pluripotent stem cell (hPSC)-derived GI organoids or GI tumoroids (Supplementary Fig. 18b–g). In Live/Dead staining to check cell viability of hPSC-derived intestinal organoids cultured in IEM hydrogel and Matrigel, the organoids grown in both matrices showed no significant difference in Live^+^ and Dead^+^ areas with more than 80% viability (Supplementary Fig. 18c), indicating that the IEM hydrogel did not provoke significant impairment in the viability of human intestinal organoids. Immunocytochemical staining of human-induced pluripotent stem cell (hiPSC)-derived intestinal organoids at days 29 and 51 indicated development of human organoids expressing intestinal stem cell marker SOX9 and differentiation markers MUC2 and CHGA in IEM hydrogel (Supplementary Fig. 18d, e). These results prove their versatile utility for culturing diverse types of organoids. In the future, the applicability of ECM hydrogels needs to be fully validated for the long-term culture and development of human GI organoids.

The long-term storage potential of reconstituted ECM is important for the convenience and commercialization of hydrogel matrices. We, therefore, checked the long-term stability and bioactivity of ECM hydrogels after refrigeration or freezing. We tested SEM and IEM pre-gel solutions stored in a deep-freezer (−80 °C for 6 months) or a refrigerator (4 °C for 1 month) for GI organoid culture (Fig. 6e, h). The organoid formation efficiency in SEM and IEM hydrogels that had been frozen for 1–6 months or refrigerated for 1–4 weeks was not different from that of freshly prepared hydrogels (Fig. 6f, i). The morphology and size of gastric and intestinal organoids grown in stored SEM and IEM hydrogels were similar to those grown in fresh hydrogels (Fig. 6g, j). Finally, gene expression levels of self-renewal and differentiation markers in GI organoids were not significantly different in long-term stored ECM hydrogels and fresh ECM hydrogels, which were similar to or higher than those in Matrigel organoids (Supplementary Fig. 19).

More interestingly, ECM hydrogels could be cryopreserved together with organoids, enabling ready-to-use applications. When organoids frozen in IEM hydrogel or Matrigel were thawed, the organoids frozen in IEM hydrogel were highly viable and grew normally, but those frozen in Matrigel exhibited significantly lower viability (Supplementary Fig. 20a, b). We then quantified the live- or dead-staining areas of organoids and confirmed that GI tissue-derived ECM hydrogel protected organoids from cellular damage caused by cryopreservation better than Matrigel (Supplementary Fig. 20c). After thawing, IEM organoids preserved their original morphology before cryopreservation, whereas Matrigel organoids exhibited an unhealthy morphology (Supplementary Fig. 20d). Likewise, the expression level of the apoptotic marker *Caspase-3* in intestinal organoids after thawing significantly increased in only Matrigel organoids (Supplementary Fig. 20e). Immunocytochemical staining 2 days after thawing intestinal organoid-laden IEM hydrogel or Matrigel indicated that the percentage of active (cleaved) caspase-3^+^ area in the whole organoid was much higher (4.8-fold) in Matrigel organoids (13.67%) than in IEM organoids (2.85%) (Supplementary Fig. 20f, g). These data suggest again that IEM hydrogel is more effective than Matrigel to reduce apoptotic signaling resulting from caspase-3 activation during freeze–thaw cycles. Previous studies demonstrated that supplementation of ECM proteins such as collagen peptides^52^ and hyaluronan^53,54^ to the freezing medium or entrapment of cells with collagen scaffolds^55,56^ could reduce damage and stress during the cryopreservation process. Thus, the protective effects of GI tissue-derived ECM hydrogels during cryopreservation of the organoids observed in our study may be owing to the presence of various matrisome proteins abundant in GI tissue ECM hydrogels that Matrigel does not contain (e.g., collagen subtypes, and hyaluronan). In addition to excellent storage potential, ECM hydrogels may be compatible with a wide variety of culture systems. For example, we successfully integrated GI tissue-derived ECM hydrogels with microfluidic devices stacked on a single rocker for high-throughput dynamic culture^57^ (Supplementary Fig. 21). We anticipate that ECM-integrated microfluidic platforms will allow for mass production of GI organoids for drug screening, toxicological evaluation, and disease modeling.

Finally, we tested the feasibility of GI tissue-derived ECM hydrogels for in vivo applications. Matrigel and cell therapeutics produced using Matrigel cannot be used in human clinical applications due to safety issues arising from the tumor-derived origin of Matrigel^11,58^. In contrast, ECM hydrogels derived from decellularized tissues with removal of cells containing antigenic genetic motifs can be used as safe scaffolds for clinical cell therapy with a low risk of immunological rejection and inflammatory responses^59,60^. To test whether ECM hydrogels aid efficient engraftment of transplanted GI organoids, we injected gastric or intestinal organoids encapsulated in their respective tissue-derived ECM hydrogels into the stomach or small intestine with acute epithelial injury using acetic acid. We first verified that the treatment of acetic acid causes epithelial damage and inflammatory reactions in the stomach and intestine tissues. H&E staining clearly showed the damaged regions in the gastric tissue treated with acetic acid and the collapse of the organization and polarization of gastric glands, which are indicative of gastric ulcer (Supplementary Fig. 22a). Likewise, the structures of villi and crypt were severely impaired in the intestinal tissue treated with acetic acid (Supplementary Fig. 22b). Tissue morphologies of mouse stomach and intestine injury models induced by acetic acid treatment were completely distinct from those of intact stomach and intestine tissues. In immunofluorescence staining for immune cell marker, F4/80^+^ macrophages were rarely observed in normal gastric and intestinal tissues, while F4/80^+^ macrophage infiltration was clearly observed in the stomach and intestine tissues of the injured models (Supplementary Fig. 22c, d). In particular, massive infiltration of F4/80^+^ macrophages was detected in the injured intestine tissue (Supplementary Fig. 22d). Next, we transplanted only SEM and IEM hydrogels to confirm negligible inflammatory responses following administration of SEM and IEM hydrogels. When SEM hydrogel labeled with a fluorescent dye (TAMRA-SE) was injected into the submucosa region of mouse stomach tissue, the hydrogel decomposed rapidly, and the fluorescence signals of the SEM hydrogel decreased after administration (Supplementary Fig. 23a). Infiltrated macrophages were not observed in the injection sites 1 and 4 days after SEM hydrogel injection. In the case of IEM hydrogel, the fluorescence signals of the IEM hydrogel distributed widely over the villi structures 4 h after injection into the intestinal lumen (Supplementary Fig. 23b). Similar to SEM hydrogel, macrophage infiltration was barely observed in the injected sites 1 and 4 days (Supplementary Fig. 23b) Taken together, these data demonstrate that SEM and IEM hydrogels can serve as biocompatible organoid carriers for transplantation.

For organoid instillation, GI tissue-derived hydrogels were diluted in culture medium (1:20) as described previously^61,62^. We utilized fluorescently labeled GI ECM hydrogels and organoids to show specific replacement of damaged tissues by transplanted GI organoids. To trace organoid growth in vivo, we used enhanced green fluorescent protein (EGFP)^+^ organoids derived from EGFP transgenic mice (Fig. 6k and Supplementary Fig. 24a, b). Injection of gastric organoids using TAMRA-labeled SEM hydrogel allowed efficient administration of organoids into the submucosa region of the stomach with ulcers (day 0, Fig. 6l). We found that 4-, 7-, and 28-days post-transplantation EGFP^+^ gastric organoids were successfully engrafted into host tissues and EGFP signals of organoids were specifically detected throughout the injured sites (Fig. 6m and Supplementary Fig. 24c). Likewise, injected EGFP^+^ intestinal organoids with TAMRA-labeled IEM hydrogel were also engrafted into the injured intestinal tissues 4 and 7 days after transplantation (Fig. 6m and Supplementary Fig. 24d). Since diluted ECM hydrogels were applied for organoid transplantation, TAMRA fluorescent signals of SEM and IEM disappeared probably due to the rapid degradation in vivo. These data suggest that SEM and IEM hydrogels could mediate efficient engraftment of GI organoids into the injured regions for epithelium reconstruction and support the use of tissue ECM hydrogels as an effective organoid carrier for GI tissue regeneration. A long-term transplantation study would be required to check-in vivo behaviors and regenerative efficacy of transplanted GI organoids in injured GI tissues.

## Discussion

In this study, we demonstrate GI tissue-mimetic matrices that can replace the use of conventional Matrigel, which is currently indispensable for GI organoid culture. Despite the effectiveness of Matrigel in culturing a wide variety of organoids, it has several critical limitations, including a lack of tissue-specific biochemical ECM factors, safety issues, and batch variation, which have prompted the development of alternative matrices for organoid culture. For example, several studies have reported synthetic or natural hydrogels (e.g., PEG, alginate, and fibrin gel) modified with ECM peptide motifs (e.g., RGD) and protease-degradable peptides (e.g., GPQGIWGQ) for culturing intestinal organoids^13–16^. These artificial matrices are reconstituted with only a few ECM components and are not capable of mimicking the intrinsic and complex matrisome of native GI tissues. ECM hydrogels derived from small intestine tissues have been used to cultivate endodermal organoids, such as gastric, hepatic, pancreatic, and small intestinal organoids^20^. The decellularization protocols used to produce these ECM hydrogels have historically used SDC to induce efficient removal of cellular components; however, ionic detergents may cause damage to the tissue ECM and impair the bioactivity and properties of ECM hydrogels, as observed in our analysis (Supplementary Fig. 1). Previously, we tested IEM hydrogels supplemented with three ECM proteins (laminin-111, laminin-511, and nephronectin) in various concentrations and combinations to investigate the effects of those ECM proteins on the formation and development of intestinal organoids^63^. Because IEM hydrogel alone did not support the formation and growth of intestinal organoids due to unoptimized decellularization protocol and culture condition^63^, our previous study did not prove the ability of IEM hydrogel comparable to Matrigel for culturing intestinal organoids. To overcome the limited roles of existing synthetic and natural hydrogels in organoid culture, we provide evidence to support the use of ECM hydrogels derived from the stomach or intestinal tissue that has been decellularized via an optimized protocol as a highly effective alternative to Matrigel. Indeed, gastric and intestinal organoids grown in SEM and IEM hydrogels underwent similar morphological development to organoids cultured in Matrigel and exhibited differentiation profiles and tissue functions comparable to Matrigel organoids (Fig. 3).

Using a proteomic approach, we defined the beneficial effects of ECM hydrogels over Matrigel, which is primarily composed of glycoproteins and lacks GI tissue-specific ECM components. More than 96% of the total matrisome proteins in Matrigel were made up of glycoproteins, and laminin-111 ranked in the top ten most abundant matrisome proteins (Fig. 2b and Supplementary Fig. 4c). In most mature tissues, however, the expression of laminin-111 is not as prominent as it is in Matrigel^64,65^, and different laminin isoforms, such as laminin-332 and laminin-511, are distributed across native GI tissues^47,48,66^ and in our GI tissue-derived ECM hydrogels (Supplementary Fig. 4c). Although collagens are the most abundant ECM proteins in native tissues, the total collagen content in the Matrigel matrisome is only 0.4% with only a few subtypes (~10 subtypes). In contrast, SEM and IEM retained a significantly greater amount of collagen (about 67% and 51% of total matrisome proteins in SEM and IEM, respectively) and a larger number of subtypes (19–20 subtypes) (Fig. 2c–e, 2h and Supplementary Fig. 4c), which more closely recapitulates the ECM environment of native GI tissues. When we examined proteins that are specifically enriched in the native stomach or intestine based on the Human Protein Atlas^67^, we detected the largest number in SEM and IEM, respectively (Supplementary Figs. 4d and 6). Interestingly, we found that the majority of native GI tissue-enriched non-matrisome proteins detected in SEM and IEM were listed in the top 10 GOBP categories for non-matrisome proteins in SEM and IEM (Supplementary Fig. 5). For example, OLFM4, APOA4, and EPS8L2 are involved in the GOBP term “cellular component biogenesis,” and SMTN, PLS1, and VIL1 belong to the GOBP term “organelle and cytoskeleton organization.” Based on the presence of many non-matrisome proteins that are abundant in native GI tissues, SEM and IEM hydrogels may better support cellular component biogenesis and structure development during organoid development in comparison to Matrigel. Collectively, the orchestration of GI tissue-specific core matrisome and non-matrisome proteins enables the reconstruction of native GI tissue-mimetic microenvironments to support the growth and development of GI organoids.

In this study, we demonstrate that tissue origin and donor age are critical factors to consider in the design of decellularized tissue-derived matrices for organoid culture. Additional proteomic analysis indicated that several core matrisome proteins were highly enriched in SEM and IEM in comparison to SkEM (Fig. 5g, h). These matrisome proteins, such as collagen VI^44^, fibronectin^45^, and laminin^68^, are known to regulate epithelial cell behaviors, including survival and differentiation. Accordingly, SEM and IEM were more effective in the development of GI organoids than SkEM (Fig. 5a–f), further emphasizing the importance of matching organoid type to the tissue origin of decellularized matrices. Moreover, proteomic analysis of ECM hydrogels derived from the same types of tissues at different ages revealed that a variety of matrisome proteins were detected differentially in decellularized tissue ECM from piglets and adult pigs despite only a 4-month difference in age (Fig. 5m, n). Specifically, we identified fibrillin 2 and tenascin C as major matrisome components in piglet SEM and IEM, respectively, and fibronectin, a component of the GI stem cell niche, was highly expressed in both piglet SEM and IEM compared to hydrogels derived from adult pigs. Recent studies reported that enrichment of fibrillin 2 and tenascin C in neonatal lung ECM promotes tissue remodeling^50^, and decrease of fibronectin in the aged muscle stem cell niche reduces muscle regeneration ability^51^. Consistent with these studies, we found that age-dependent differences in tissue matrisome components affected the self-renewal ability and growth potential of GI organoids, which was indicated by increased expression of stemness markers (*Lgr5*, *Axin2*, and *Olfm4*) in GI organoids cultured in piglet ECM hydrogels enriched with these factors (Fig. 5j, l). Our results provide important insight into ECM remodeling during aging and suggest that age should be considered in the preparation of effective decellularized matrices for organoid development and the rejuvenation of GI epithelium.

Overall, our proteomic analytical data provide biological insight into the development of chemically-defined hydrogels for organoid culture by identifying critical matrisome elements that positively influence organoid formation and development. Hydrogels inspired by tissue-specific ECM peptide motifs not only provide 3D native tissue-mimetic matrisome complexity for organoid maturation and improved function but also solve several inherent limitations of naturally derived materials, including variation and mass production challenges. Decorin, biglycan, and lumican were commonly present in the top ten most abundant matrisome proteins of all batches of SEM or IEM samples (Fig. 2c–e, h), which are known to play important roles in collagen fibril formation^69^, cell proliferation^70^, maturation^71^, and epithelium homeostasis^72,73^. Therefore, synthetic hydrogels modified with these matrisome proteins may be optimal matrices for GI organoid culture and will be explored in future work. Through in-depth proteomic analysis, we also identified several matrisome proteins that have been known to be highly upregulated in piglet ECM in comparison to adult pig ECM (e.g., FN and TNC), which can be used as the ECM components for the functionalization of chemically defined hydrogels. Actually, we found that addition of FN into adult pig GI tissue-derived ECM hydrogel could significantly improve the quality of organoids, which is similar level to those grown in piglet GI tissue-derived ECM hydrogel (Supplementary Fig. 17). This result indicates that FN may be a crucial factor involved in the ability of piglet ECM to better support organoid culture. Thus, supplementation of those crucial ECM components into synthetic polymer hydrogels such as PEG and alginate can be considered. Future studies to systemically identify more potent candidate ECM molecules and optimal ECM concentrations would be able to facilitate the development of non-xenogeneic, chemically defined hydrogels for GI organoid culture. Given a recent trend to reduce animal use in scientific research, this kind of study would be a highly valuable project.

Finally, we demonstrated the versatility and practicality of GI tissue-derived ECM hydrogels for organoid culture and transplantation. SEM and IEM hydrogels allowed for the long-term subculture of GI organoids (Fig. 6a–d) and supported long-term expansion to produce a large number of organoids, which is necessary for disease modeling and regenerative medicine. We found that intestinal organoids cultured in IEM hydrogel exhibited less budding compared to Matrigel organoids. Since small intestinal organoids grown in 3D collagen matrix were previously reported to show morphology with fewer budding^74,75^, IEM hydrogel with highly enriched collagen components was expected to induce such morphology of intestinal organoids as observed in previous studies. Although intestinal organoids grown in IEM hydrogel have an architecture with smaller buds, the gene expression levels of stem cell markers and intestinal differentiation markers are comparable to those of Matrigel organoids (Fig. 3i, j). In addition, after several passages, we observed that the number of intestinal organoids with budding structures increased in IEM hydrogel (Fig. 6c). The functionality of ECM hydrogels as organoid culture matrices was retained even after long-term storage at either −80 °C (6 months in a deep-freezer) or 4 °C (1 month in a refrigerator) (Fig. 6e–j), allowing for multiple uses of pre-gel ECM solution. More interestingly, organoids could be cryopreserved within the ECM hydrogel and thawed without loss of ECM bioactivity or organoid viability (Supplementary Fig. 20), substantially increasing the off-the-shelf availability of organoid culture platforms. Furthermore, SEM and IEM hydrogels facilitated the engraftment of transplanted organoids in injured GI tissues (Fig. 6k–m and Supplementary Fig. 24c, d). Several genes involved in wound healing were upregulated in GI ECM organoids in comparison to Matrigel organoids (Fig. 4g), suggesting that organoid transplantation using GI tissue-derived ECM hydrogels has the potential to provide highly effective therapeutics to address incurable GI diseases, such as inflammatory bowel disease, Crohn’s disease, and gastric ulcers as well as tissue defects following tumor resection. Our strategy to provide tissue-specific ECM hydrogels can be extended into other types of tissue organoids with limitations in Matrigel culture (e.g., liver organoids).

In summary, we demonstrate the potential of IEM and SEM hydrogels as alternatives to Matrigel for GI organoid culture. Our strategy using animal tissue-derived ECM hydrogels did not fully address the translational limitation of Matrigel due to their animal origin. Although both Matrigel and decellularized animal tissue-derived materials are prepared from xenogeneic sources, unlike Matrigel of mouse tumor origin, decellularized matrix derived from non-tumor tissues can be customized to the clinical setting through a well-controlled decellularization process. In this study, we provide experimental evidence on how our approach with decellularized GI tissue-derived ECM hydrogels can resolve the major issues of Matrigel including (1) batch-to-batch variation, (2) safety, and (3) high-cost issues. Our proteomic analysis revealed that decellularized GI tissue-derived ECMs have a relatively low batch-to-batch variation and donor variation if porcine donor conditions (e.g., age and gender) are controlled for sourcing GI tissues (Fig. 2e–n and Supplementary Fig. 6). We also confirmed that our ECM hydrogels show clinically acceptable levels of endotoxin and marginal immunogenicity both in vitro and in vivo due to the absence of xenogeneic pathogens and antigenic cellular components (Supplementary Fig. 3). Cost estimation from our calculation considering the price of porcine GI tissues indicates that decellularized GI tissue-derived ECM hydrogels are much more cost-effective for GI organoid culture than Matrigel (Supplementary Table 1). Given that decellularized animal tissues or their derivatives have been approved and commercialized for clinical applications to humans, decellularized tissue-derived ECM hydrogels that can address critical limitations of Matrigel will be available for clinically relevant organoid culture. Nevertheless, GI ECM hydrogels have potential limitations in translational applications, which need to be carefully investigated and addressed as well. Intestinal organoids cultured in IEM hydrogel exhibited less budding, compared to those cultured in Matrigel, which may indicate a rather premature state of IEM organoids. Considering that IEM organoids showed higher expression of stem cell markers than Matrigel organoids, we cannot exclude that a larger portion of precursor cells still exist in IEM organoids. Despite our data on the viability of human intestinal organoids, more in-depth studies to fully check the effects of GI ECM hydrogels on human GI organoids would be inevitable in the future.

## Methods

### Decellularization of porcine tissues

Porcine stomach and small intestine tissues were freshly obtained from a local market (Majang Meat Market, Seoul, Korea). The experiments using porcine tissues were conducted according to ethical principles and standard guidelines of the Institutional Animal Care and Use Committee (IACUC) (Pain Category A; Use of non-living tissues). The fat tissues and large blood vessels were removed prior to decellularization. All layers of stomach tissue were used for the study, whereas mesentery and serosa layers were removed from intestine tissue. Stomach tissue was chopped into small pieces (≈0.3 × 0.3 × 0.3 cm^3^), and intestine tissue was cut longitudinally and then into short segments (10 cm). Two protocols with different chemical treatments were tested for decellularization of GI tissues; Protocol 1 is the one we optimized, and Protocol 2 is the commonly used one. For Protocol 1, both prepared tissues were agitated in buffer solutions (500 ml) in the following order: distilled water for 3 h; 1% (v/v) Triton X-100 (Wako, Osaka, Japan) with 0.1% (v/v) ammonium hydroxide (Sigma, St. Louis, MO, USA) for 48 h; distilled water for 24 h; 1% (v/v) penicillin-streptomycin (P/S; Thermo Fisher Scientific, Waltham, MA, USA) for 2 h; and distilled water for 1 h. For both tissues, every solution was removed from the tissues before the next step, and all processes were performed at 4 °C and agitated on a rotator at 180 revolutions per minute (r.p.m.). The sterilized decellularized tissues were then lyophilized and stored at 4 °C until use. Protocol 2 was carried out as previously established^20^ by agitating prepared tissues in buffer solutions (500 ml) in the following order: 4% (w/v) SDC (Sigma) for 3 h; distilled water for 24 h with 4–6 water changes; 2000 kU DNase-I (Sigma) in 1 M sodium chloride (Sigma) for 3 h; and distilled water for 48 h. All processes were performed at room temperature and agitated on a rotator at 180 r.p.m. The decellularized tissues from Protocol 2 were also lyophilized and stored at 4 °C until use. The mucosal tissues of the porcine stomach and small intestine were decellularized. The mucosal layer of the stomach was separated from the rest of the stomach and chopped into small pieces (≈0.3 × 0.3 × 0.3 cm^3^). The pieces of the mucosal layer of the small intestine were obtained by scrubbing the inner lumen of the intestine with a slide glass. The decellularization of mucosal tissues was conducted according to Protocol 1. Decellularization of other porcine tissues (esophagus, skin, lymph, heart, and muscle) was carried out as previously described^76–79^. The harvested tissues were also cut into small pieces (≈0.3 × 0.3 × 0.3 cm^3^) and all processes for decellularization of each tissue were performed at 4 °C with agitation at 180 r.p.m. as follows if not stated otherwise: esophagus—0.5% (v/v) trypsin/ethylenediaminetetraacetic acid (EDTA, Thermo Fisher Scientific) for 2 h, 1 M sucrose (Sigma) for 30 min, 3% (v/v) Triton X-100 (Sigma) for 48 h, and 10% (v/v) SDC for 4 h; skin—0.25% (v/v) trypsin/EDTA for 1 h at 37 °C, 70% (v/v) ethanol (Duksan Pure Chemicals, Ansan, Korea) for 6 h, and 1% (v/v) Triton X-100 (Sigma) with 0.1% (v/v) ammonium hydroxide for 72 h; axillary lymph node—0.1% (v/v) sodium dodecyl sulfate (SDS; Wako) for 12 h; skeletal muscle—1% (v/v) Triton X-100 (Sigma) with 0.2% (v/v) SDC for 6 h; cardiac muscle—1% (v/v) SDS for 48 h. The rest of the decellularization steps include washing with distilled water, 1% (v/v) P/S treatment for 2 h, washing with distilled water, and finally decellularized tissues were lyophilized. The decellularized tissues were lyophilized using a freeze dryer (FDU-2100, Eyela, Tokyo, Japan) under the operating conditions of a vacuum gauge of 5.5 Pa and a trap temperature of −85 °C.

### Characterization of decellularized tissues

To confirm the decellularization of GI tissues, DNA assays and histological analyses were performed after decellularization. Removal of cellular components was confirmed by comparing DNA content in the native and decellularized GI tissues using a DNA extraction kit (Bioneer, Daejeon, Korea). The GAG content was quantified using 1,9-dimethyl methylene blue dye solution assay (Sigma) as previously reported^80^. Both native and decellularized tissues were digested using papain (125 µg ml^−1^, Sigma) and diluted with distilled water. The sample solutions were treated with 1,9-dimethyl methylene blue dye, and the absorbance of the samples was measured at 525 nm using a microplate reader (Tecan, Männedorf, Switzerland). Chondroitin sulfate A (Sigma) was used as a standard serial dilution starting from a concentration of 2 µg ml^−1^. For histological analyses, both native and decellularized GI tissues were fixed with 4% (w/v) paraformaldehyde (Sigma) for 1 day, embedded in paraffin, and then sliced into 4-µm sections for H&E staining.

### Protein sample preparation for MS analysis

The lyophilized tissues were reconstituted with lysis buffer [4% SDS, 0.1 M Tris-HCl (pH 7.6), and 1× protease inhibitor (Sigma) in 500 μl] and incubated at room temperature for 10 min. Subsequently, the tissues were boiled for 15 min at 95 °C on a heat block. Tissue lysis was performed on ice by sonication using a probe sonicator (Qsonica Q125, Newtown, CT, USA) and centrifuged at 14,000×*g* at 4 °C for 20 min. The supernatant was collected and transferred to a new tube. Protein concentration was measured using the BCA protein assay (BCA Protein Assay Kit, Thermo Fisher Scientific). Matrigel, SEM, and IEM (100 μg) protein samples were digested using Filter Aided Sample Preparation^81^. Proteins were reduced in SDT buffer [4% SDS in 0.1 M Tris-HCl, pH 7.6 and 0.1 M dithiothreitol (DTT)] at 37 °C for 45 min and boiled for 10 min at 95 °C on a heat block. The protein samples were then sonicated for 10 min in a bath sonicator (Diagenode, Denville, NJ, USA) followed by centrifugation at 14,000 × *g* at 20 °C for 5 min. The supernatant was combined with 200 μl of 8 M urea (in 0.1 M Tris-HCl, pH 8.5) and transferred to a membrane filter (YM-30, Millipore Corporation, Burlington, MA, USA). The SDS was removed by centrifugation at 14,000 × *g* at 20 °C for 60 min, and the protein samples remained on the membrane filter. SDS removal was repeated three times. Subsequently, cysteine alkylation was performed with 100 μl of 0.05 M iodoacetamide in 8 M urea for 25 min at room temperature in the dark, followed by centrifugation at 14,000 × *g* at 20 °C for 30 min. The protein samples were washed three times with 200 μl of 8 M urea. Finally, 100 μl of 50 mM ammonium bicarbonate (pH 8.0) was added to the filter, followed by centrifugation at 14,000 × *g* at 20 °C for 30 min three times. Trypsin (Promega, Madison, WI, USA) was added to the filter at an enzyme to protein ratio of 1:50 (w/w), and the proteins were digested at 37 °C overnight. The resulting peptides were then eluted by centrifugation at 14,000 × *g* at 20 °C for 30 min. The filter was rinsed with 75 μl of 50 mM ammonium bicarbonate and centrifuged at 14,000 × *g* at 20 °C for 20 min. This step was repeated three times. The eluents were combined and dried.

### Liquid chromatography with tandem MS (LC-MS/MS) analysis

Each peptide sample was desalted with a C18 spin column (Thermo Fisher Scientific). All peptide samples were resuspended in 0.1% formic acid (FA) in water and analyzed using the Q Exactive Plus Mass Spectrometer coupled with the EASY-nLC 1000 system (Thermo Fisher Scientific). Solvent A and B were 0.1% FA in water and 0.1% FA in acetonitrile, respectively. A 200-min gradient (from 5% to 35% solvent B over 150 min, from 35% to 50% solvent B over 10 min, from 50% to 80% solvent B for 5 min, holding at 80% solvent B for 10 min, and equilibrating the column at 5% solvent B for 25 min) was used for global profiling. The peptides were eluted through a trap column and ionized with an EASY-spray column (50 cm × 75 μm ID, Thermo Fisher Scientific) that was packed with 2 μm C18 particles at an electric potential of 1.8 kV. Full MS data were acquired in a scanning range of 300–1600 Th at a resolution of 70,000 at m/z 200 with an automated gain control target value of 1.0 × 10^6^ and a maximum ion injection of 60 ms. The maximal ion injection time for MS/MS was set to 100 ms at a resolution of 17,500. The dynamic exclusion time was set to 30 s. For the MS/MS scans, the 10 most abundant ions were fragmented by nitrogen gas with an isolation window of 0.8 Th and normalized collision energy of 32 for higher-energy collisional dissociation.

### Peptide and protein identification

Mass spectra were processed using MaxQuant (1.6.10.43)^82^. MS/MS spectra were queried against the target-decoy Mouse Uniprot database (released in March 2020) for Matrigel and against the *Sus scrofa* (pig) Uniprot database (released in March 2020) for porcine intestine and stomach tissues using the Andromeda search engine^83,84^. Primary searches were performed using a 4.5 ppm precursor ion tolerance for total protein level analysis. The MS/MS ion tolerance was set to 20 ppm. Cysteine carbamidomethylation was set as a fixed modification. N-acetylation of protein and oxidation of methionine were set as variable modifications. Enzyme specificity was set to full tryptic digestion. Peptides with a minimum length of six amino acids and up to two missed cleavages were considered. The required FDR was set to 1% at the peptide, protein, and modification level. The Intensity Based Absolute Quantification (iBAQ) algorithm^85^ was used for quantification as a part of the MaxQuant platform. Briefly, iBAQ values represent the raw intensities divided by the number of theoretical peptides. Thus, iBAQ values are proportional to the molar quantities of the proteins. The iBAQ values were converted to relative iBAQ values for comparison across different samples. Label-free quantification (LFQ) values that represent normalized peptide intensities were used for mapping volcano plots when comparing adult and piglet proteins. The Perseus software^86^ was used to transform LFQ values into log_2_ data, which were then calculated for fold changes, and *p*-values were adjusted based on FDR less than 5%. The identified proteins were compared and categorized in reference to the Human Protein Atlas^67^ and the Matrisome Project^87^. A gene ontology (GO) search was performed using g:Profiler software (ELIXIR, Hinxton, Cambridgeshire, UK)^88^. GOBP analyses were performed on the commonly expressed matrisome proteins in the samples from porcine A–C, the proteins that show a significantly elevated level of expression in stomach or intestine tissue compared to any other tissue^67,89,90^, and the non-matrisome proteins identified in Matrigel, SEM, and IEM. PANTHER Overrepresentation Test^91–93^ (Released 20210224) was selected as the statistical tool and GO biological process complete was chosen as the dataset for the GOBP analysis. Fisher’s exact test with FDR correction as calculated by the Benjamini-Hochberg procedure was performed. Biological processes with FDR < 0.05 were considered as significant.

### 3D ECM hydrogel characterization

Solubilized decellularized GI tissues were constructed into 3D hydrogels by temperature-induced self-assembly. Decellularized tissue-derived ECM solutions (10 mg ml^−1^) were prepared by digestion using pepsin (4 mg ml^−1^, Sigma) in 0.02 M hydrochloric acid (HCl, Sigma) for 48 h. The pre-gel solution was prepared by mixing the decellularized tissue-derived ECM solution with 10% (v/v) 10× phosphate-buffered saline (PBS, Sigma) and adjusting the pH to 7.4 using 0.5 M sodium hydroxide (NaOH, Sigma) solution. ECM fibril crosslinking was induced by incubating the pre-gel solution at 37 °C for 30 min. The microstructure of the assembled hydrogels was visualized with a field emission scanning electron microscope (FE-SEM, JEOL-7800F, JEOL Ltd., Tokyo, Japan) after serial dehydration of the hydrogels. The viscoelastic moduli of the hydrogels were evaluated using a rotating rheometer (MCR 102, Anton Paar, Ashland, VA, USA). Briefly, the hydrogels were placed onto the measuring plate, and the viscoelastic modulus was measured with an 8 mm parallel plate at a constant strain (1%) using a frequency sweep mode (0.1–10 Hz). The elastic modulus of the hydrogel was determined by calculating the average storage modulus of the hydrogel at 1 Hz. To check the effect of piglet ECM, the pre-gel solution was supplemented with 25–50 µg ml^−1^ fibronectin (Thermo Fisher Scientific) and/or 25–50 µg ml^−1^ Tenascin C (R&D Systems, Minneapolis, MN, USA) prior to cross-linking.

### In vitro and in vivo biocompatibility tests of ECM hydrogels

Endotoxin tests of GI ECM hydrogels were performed using Pierce^™^ Chromogenic Endotoxin Quant Kit (Thermo Fisher Scientific) according to the manufacturer’s protocol. Pre-gel solutions of SEM hydrogel (5 mg ml^−1^) and IEM hydrogel (2 mg ml^−1^) were used for the tests and the absorbance of final samples was measured at 405 nm using a microplate reader (Tecan). For evaluating the immunogenicity of GI ECM hydrogels, inflammatory cytokine (TNF-α) secretion from RAW 264.7 macrophages co-cultured with GI ECM hydrogels (5 mg ml^−1^ SEM hydrogel and 2 mg ml^−1^ IEM hydrogel) was examined. The levels of secreted TNF-α were determined using a mouse TNF-α enzyme-linked immunosorbent assay (ELISA) kit (R&D Systems). Co-culture of macrophages with ECM hydrogels was performed with a 24-well transwell insert (Corning, Corning, NY, USA). First, macrophages were seeded on the bottom of the 24-well plate, and ECM hydrogels (50 µl) were then loaded on the transwell insert. Macrophages cultured without ECM hydrogel (no treatment) and macrophages treated with 1 µg ml^−1^ lipopolysaccharide (LPS, Sigma) served as negative and positive control groups, respectively. The cells were cultured for 3 or 6 h, and the cultured medium was harvested and tested with a TNF-α ELISA kit using the manufacturer’s protocol. To check immunogenicity in vivo, GI ECM hydrogels (5 mg ml^−1^ SEM hydrogel and 2 mg ml^−1^ IEM hydrogel) were subcutaneously injected into the back of the mouse. Skin tissues with hydrogel injection were harvested at 1, 4, and 7 days after the injection. The retrieved tissues were fixed with 4% (w/v) paraformaldehyde (Sigma) for 1 day, embedded in paraffin, and then sliced into 4-µm sections. H&E staining and toluidine blue staining of tissue sections was performed to check whether there are tissue damage and recruitment of inflammatory cells in the regions injected with ECM hydrogels.

### Isolation of tissue-derived gastric organoids and intestinal organoids

For gastric and intestinal organoid culture, 6–8-week-old C57BL/6 male mice (Nara Biotech, Seoul, Korea) were sacrificed, and stomach and small intestine tissues were harvested. All mice were maintained in the housing condition with a temperature of 21 ± 2 °C, a humidity of 50 ± 10%, ventilation of 10–15/h, the light of 150–300 Lux, and noise of less than 60 dB. These procedures were approved by the IACUC of the Yonsei Laboratory Animal Research Center (YLARC) (permit number: IACUC-A-201612-540-04 and IACUC-201807-767-03). The harvested tissues were fragmented using scissors and then washed with ice-cold Dulbecco’s PBS (DPBS, Sigma). The stomach and small intestinal tissue pieces were incubated and rocked in 10 mM or 2 mM EDTA solution, respectively, for 30 min at 4 °C. Additionally, slide glasses were used to mechanically dissociate gastric glands. In the case of colon tissue, the pieces were treated with 20 mM EDTA solution and incubated with rocking at 4 °C for 40 min. After several washes in DPBS, the number of glands from the stomach and crypts from the intestine were counted and encapsulated in growth factor reduced Matrigel (Corning) or decellularized GI tissue-derived ECM hydrogels. Culture medium was added to the organoid-laden hydrogel constructs in each well of a 48-well plate. The efficiency of organoid formation in each hydrogel was calculated as a percentage ratio of the number of formed organoids to the number of seeded glands or crypts.

### GI organoid culture

The culture media for both gastric and intestinal organoids were made from the same basal medium, which was composed of advanced DMEM/F12 (Dulbecco’s Modified Eagle Medium/Ham′s F-12) supplemented with 10 mM HEPES (N-2-hydroxyethylpiperazine-N-2-ethane sulfonic acid), 2 mM GlutaMax, and 100 U/ml P/S (all from Thermo Fisher Scientific). Mouse gastric organoid culture medium was composed of the basal medium supplemented with 50% (v/v) Wnt3a-conditioned medium, 10% (v/v) R-spondin1-conditioned medium, 1× B27 (Thermo Fisher Scientific), 100 ng ml^−1^ mouse Noggin (Peprotech, Rocky Hill, NJ, USA), 100 ng ml^−1^ human fibroblast growth factor-10 (FGF10, Peprotech), 50 ng ml^−1^ mouse epidermal growth factor (EGF, Thermo Fisher Scientific), 10 nM [Leu15]-Gastrin I (Sigma), 1 mM N-acetyl cysteine (Sigma), and 10 µM Y-27632 (initial 3 days only; BioGems International, Inc., Westlake Village, CA, USA). Mouse small intestinal organoid culture medium was composed of the basal medium supplemented with 10% (v/v) R-spondin1-conditioned medium, 1× N2 (Thermo Fisher Scientific), 1× B27, 100 ng ml^−1^ mouse Noggin, 50 ng ml^−1^ mouse EGF, 1 mM N‐acetyl cysteine, and 10 µM Y-27632 (initial 2 days only; BioGems International, Inc.). For mouse colonic organoid culture medium, the small intestinal organoid culture medium was supplemented with 50% (v/v) Wnt3a-conditioned medium and 100 µg ml^−1^ primocin (InvivoGen, San Diego, CA, USA). The wnt3a-conditioned medium was produced using the L-Wnt-3A cell line (CRL-2647, American Type Culture Collection (ATCC), Manassas, VA, USA) according to the protocol of the manufacturer. The R-spondin1-conditioned medium was produced using HEK293T cells stably expressing Rspo1-Fc generated in Calvin Kuo’s Laboratory at Stanford University. These cells were cultured in DMEM containing 10% (v/v) fetal bovine serum (FBS, Thermo Fisher Scientific) and 100 U/ml P/S. Cell line selection was performed using 0.4 mg ml^−1^ Geneticin^TM^ Selective Antibiotics for the selection of L-Wnt-3A and 0.3 mg ml^−1^ Zeocin^TM^ Selection Reagent for the selection of Rspo1-Fc (all from Thermo Fisher Scientific). For long-term culture of gastric organoids in SEM hydrogel, 10 mM nicotinamide (Sigma) and 2 µM A83-01 (Tocris, Bristol, United Kingdom) were added to the gastric organoid culture medium. To passage gastric and intestinal organoids, Cell Recovery Solution (Corning) was applied at 4 °C for 1 h to degrade Matrigel, and 2 mg ml^−1^ collagenase IV (Sigma) was applied at 37 °C for 1 h to decompose GI ECM-based hydrogels. After incubation, the collected organoids were washed and fragmented using DPBS and encapsulated into fresh hydrogels. The passage of GI organoids in SEM and IEM hydrogels was performed every 4–7 days, which was the same as the passage cycle of GI organoids in Matrigel. The organoids cultured in SEM and IEM hydrogels were passaged at a split ratio of 1:5 to 1:7, which was also the same as Matrigel organoids.

### Immunocytochemical staining of organoids

Immunofluorescent staining was performed with 5-day cultured gastric organoids and 6-day cultured intestinal organoids. For whole-mount staining of cultured organoids, encapsulating hydrogels were degraded using Cell Recovery Solution or 2 mg ml^−1^ collagenase IV. Collected organoids were fixed in 4% paraformaldehyde for 1 h, treated with 0.5% Triton X-100 (Sigma) for 1 h, and then blocked in 5% bovine serum albumin (MP Biomedicals, Solon, OH, USA) with 2% horse serum (Thermo Fisher Scientific) for 3 h. These procedures were performed at room temperature. The gastric and intestinal organoids were then incubated for 24 h at 4 °C with following primary antibodies: rabbit anti-KI67 (#ab15580, 1:1000, Abcam, Cambridge, United Kingdom), rabbit anti-SOX9 (#AB5535, 1:500, Millipore), mouse anti-MUC5AC (#ab3649, 1:200, Abcam), rabbit anti-Chromogranin A (CHGA, #ab15160, 1:200, Abcam), mouse anti-H^+^/K^+^-ATPase (HK, #D032-3, 1:200, MBL International Corporation, Woburn, MA, USA), mouse anti-MUC2 (#sc-15334, 1:200, Santa Cruz Biotechnology, Dallas, TX, USA), rabbit anti-Lysozyme (LYZ, #ab108508, 1:250, Abcam), mouse anti-VILLIN (#sc-58897, 1:200, Santa Cruz Biotechnology), mouse anti-ECAD (#14472 S, 1:200, Cell Signaling Technology, Danvers, MA, USA), rabbit anti-ECAD (#3195S, 1:200, Cell Signaling Technology), rabbit anti-ZO1 (#61-7300, 1:50, Thermo Fisher Scientific), mouse anti-YAP1 (#sc-101199, 1:200, Santa Cruz Biotechnology), rabbit anti-Cleaved Caspase-3 (#9661S, 1:400, Cell Signaling Technology), rat anti-F4/80 (#ab6640, 1:100, Abcam), and rabbit anti-GFP (#598, 1:500, MBL International Corporation). After three washes in PBS, organoids were incubated for 24 h at 4 °C with secondary antibodies: anti-mouse Alexa Fluor 488 (#A11001, 1:200, Thermo Fisher Scientific), anti-mouse Alexa Fluor 594 (#A11005, 1:200, Thermo Fisher Scientific), anti-rabbit Alexa Fluor 488 (#A11008, 1:200, Thermo Fisher Scientific), anti-rabbit Alexa Fluor 594 (#A11012, 1:200, Thermo Fisher Scientific), and anti-rat Alexa Fluor 488 (#A11006, 1:200, Thermo Fisher Scientific). F-actin staining was performed for 24 h at 4 °C with TRITC-conjugated phalloidin from the Focal Adhesion Staining Kit (1:200, Millipore). Organoids were washed three times in PBS, and nuclei were stained with 2-(4-amidinophenyl)indole-6-carboxamidine dihydrochloride (DAPI, TCI, Tokyo, Japan). Images of stained organoids were acquired by confocal microscopy (LSM 880, Carl Zeiss, Jena, Germany).

### qPCR analysis

The mRNA expression of each organoid was quantified by qPCR analysis. mRNA samples were extracted from 5-day cultured gastric organoids or 6-day cultured intestinal organoids using the TaKaRa MiniBEST Universal RNA Extraction Kit (TaKaRa, Shiga, Japan), and cDNA samples were synthesized from the extracted mRNA using a cDNA synthesis kit (TaKaRa). Then, qPCR analysis was performed using cDNA samples and TaqMan Fast Universal PCR Master Mix (Applied Biosystems, Foster City, CA, USA) on a StepOnePlus Real-Time PCR System (Applied Biosystems). The following TaqMan gene expression assay kits were used for qPCR: *Lgr5* (Mm00438890_m1), *Pgc* (Mm00482488_m1), *Atp4a* (Mm00444417_m1), *Atp4b* (Mm00437657_m1), *Axin2* (Mm00443610_m1), *Muc6* (Mm00725165_m1), *Gif* (Mm00433596_m1), *Pga5* (Mm01208256_m1), *Muc2* (Mm00458299_m1), *Olfm4* (Mm01320260_m1), *Lyz1* (Mm00657323_m1), *Chga* (Mm00514341_m1), *Vil1* (Mm00494146_m1), and *Casp3* (Mm01195085_m1). The kit for mouse glyceraldehyde 3-phosphate dehydrogenase (*Gapdh*; Mm99999915_g1) was used for the normalization of the gene expression of each target marker.

### Organoid structural and functional analyses

To confirm the presence of parietal cells in the gastric organoids, the ultrastructure of gastric organoids was observed using field emission-scanning electron microscopy (FE-SEM, Teneo VS, FEI, Hillsboro, OR, USA). Before imaging, the blocks containing gastric organoids were sectioned using an ultramicrotome (MTXL, RMC, Tucson, AZ, USA). For acridine orange analysis of gastric organoids to assess acid secretion, acridine orange base (Sigma) was dissolved in dimethyl sulfoxide (DMSO, Sigma) to make a 10 mM stock solution, and the stock solution was diluted 1:1000 with gastric organoid culture medium for further use. Gastric organoids were treated with acridine orange solution (10 µM) 1 h prior to imaging analysis using a confocal microscope. The excitation range of green fluorescence and red fluorescence were 500–550 nm and 600–650 nm, respectively. Quantitative analysis was performed by calculating the average fluorescence ratio (*F*_600-650_/*F*_500-550_) of each organoid. To further validate the acid secretion of parietal cells in gastric organoids in response to histamine treatment, gastric organoids in SEM hydrogel or Matrigel were incubated with 100 µM histamine (Sigma) for 1 h. The fluorescence images of gastric organoids pretreated with acridine orange were obtained and the fluorescence intensities before and after the treatment of histamine were compared. For forskolin-induced swelling assays in intestinal organoids to assess the regulation of luminal fluid secretion, IEM hydrogel or Matrigel organoids were incubated in a culture medium containing 10 µM calcein-AM (Thermo Fisher Scientific) for 60 min. After calcein staining, 5 µM forskolin was added, and the size of the organoids was measured every 2 min using an inverted microscope [EVOS^®^ FL Auto Imaging System (Thermo Fisher Scientific)]. Organoid area analysis was performed using ImageJ software (National Institutes of Health, Bethesda, MD, USA).

### RNA sequencing

RNA sequencing analysis was performed to compare overall mRNA expression levels in organoids grown in Matrigel, organoids grown in GI tissue-derived hydrogels, and GI tissues. Comparisons were made using freshly isolated GI tissues from 6–8-week-old male C57BL/6 mice. RNA samples were extracted from organoids and tissues using the TaKaRa MiniBEST Universal RNA Extraction Kit (TaKaRa). The majority of the analysis was conducted by DNA Link, Inc. (Seoul, Korea) as follows. The Novaseq 6000 platform was used for sequencing, and the Illumina Truseq stranded mRNA kit (Illumina, San Diego, CA, USA) was used to prepare the sequencing libraries. For data processing, base calling software bcl2fastq2 (Version 2.20) with ASCII Q-score (offset 33) was used. Tophat was used to map reads, and cuffdiff was used to quantify gene expression^94^. DEGs were selected with the following parameters: fold change > 2, *p* value < 0.05, and FDR < 0.1. Functional annotation for gene ontology enrichment analysis was conducted based on the DAVID program (https://david.ncifcrf.gov). DEGs were visualized with a heat map using Cluster 3.0 and Java TreeView 1.2.0.

### Microfluidic device fabrication

The microfluidic devices were fabricated using polydimethylsiloxane (PDMS) by soft lithography as previously described^57^. Briefly, PDMS pre-polymer (Sylgard 184; Dow Corning, Midland, MI, USA) was combined with a curing agent (1:10, volume ratio, Dow Corning) and poured into photolithographed silicon wafers or Petri dishes to cast patterned PDMS layers and the bottom PDMS film. After degassing in a vacuum chamber, the PDMS samples were cured in a drying oven at 70 °C for 4 h. The fully cross-linked PDMS layer was peeled from the wafer and punched to form inlets, outlets, and chambers as demonstrated in Supplementary Fig. 21. The PDMS layers were sterilized in an autoclave and thoroughly dried under ultraviolet light before assembly. After carefully removing the dust from the PDMS layers, oxygen plasma (CUTE; Femto Science, Seoul, Korea) was applied to the PDMS layer surfaces, and the plasma-treated PDMS layers were bound to the PDMS film or other PDMS layers. The assembled devices were then placed in a drying oven at 70 °C overnight. To culture GI organoids, extracted glands and crypts were seeded in SEM or IEM hydrogel and incubated in the prepared microfluidic devices. The culture medium was carefully added to chambers through the microchannels between chambers. The microfluidic devices containing GI organoid-laden ECM hydrogels were placed on a rocker (Lab Companion, Jeio Tech, Daejeon, Korea) at 37 °C with 5% CO_2_ for dynamic culture.

### In vivo GI injury and organoid transplantation

EGFP^+^ gastric and intestinal organoids were generated from 6-week-old male C57BL/6-tg(CAG-EGFP) mice (Orient Bio Inc., Seongnam, Korea) for in vivo tracking. To monitor ECM hydrogels in vivo, SEM or IEM hydrogel was labeled with 5-(and-6)-carboxytetramethylrhodamine succinimidyl ester [5(6)-TAMRA-SE, Thermo Fisher Scientific], following modified procedures of the previously described protocol^95^. Briefly, the ECM solution prepared by 4 mg ml^−1^ pepsin–HCl treatment was mixed with 0.1 M sodium bicarbonate buffer (pH 9) in the ice-cold bath. Then, a 5(6)-TAMRA-SE solution in DMSO was added to the ECM solution at a mass ratio of 200:1 (ECM:TAMRA-SE) and the mixture was agitated at 4 °C for 24 h in the dark. After removing unreacted dye molecules via dialysis (Cellu Sep T2, MW cut-off 6–8 kDa, Membrane Filtration Products Inc., Seguin, TX, USA) against an acidified PBS and triple distilled water for 24 h, the fluorescently labeled ECM was lyophilized before use.

For organoid transplantation, 6-week-old male BALB/c-nude (CAnN.Cg-Foxn1nu/CrljOri) mice (Orient Bio Inc.) were used and these procedures were approved by the IACUC of the YLARC (permit number: IACUC-A-201904-889-02). After the mice were anesthetized with a mixture of ketamine (100 mg/kg, Yuhan, Seoul, Korea) and xylazine (16 mg/kg, Bayer Korea, Seoul, Korea), the stomach was exposed by a midline incision, and stomach ulcers were induced using 100% acetic acid (Sigma) as the previous reported^96^. The stomach was exposed to acetic acid for 30 seconds using a capillary tube, and 600–800 gastric organoids in 50 µl of diluted SEM hydrogel were immediately injected into the damaged region by submucosal injection. Intestinal injury models were also induced by acetic acid exposure with minor modifications to the previously reported protocol^97–99^. To create an intestinal ulcer, a filter paper disk (3.14 mm^2^) soaked in 50% acetic acid was placed on the intestine for 8 s. After washing the injured area three times with saline, 600–800 intestinal organoids in 40 µl of diluted IEM hydrogel were injected into the damaged region using an insulin syringe. The GI organoid-containing ECM solutions injected into the injured sites formed ECM hydrogels via crosslinking at a body temperature (37 °C). The mice were sacrificed on the day of transplantation and 4, 7, and 28 days after transplantation for histological analysis. The harvested tissues were fixed with 4% paraformaldehyde for 1 day, embedded in OCT compound (CellPath, Newtown, United Kingdom), and then sliced into 10 µm sections for fluorescence imaging. Nuclei were stained with DAPI, and images of the stained organoids were acquired by confocal microscopy.

### Generation of hPSC-derived organoids

Human embryonic stem cells (hESCs, line WA09) were obtained from the WiCell Research Institute (Madison, WI, USA). hiPSCs (line WT3 and line KYOU-DXR0109B (ACS-1023)) were obtained from the Yonsei University College of Medicine and ATCC, respectively. Studies involving these cell lines were approved by the Institutional Review Board of Yonsei University (Permit Number: 7001988-202006-ES-904-01E and 7001988-202106-BR-1230-01E). All hPSC lines were cultured on dishes coated with hESC-qualified Matrigel (Corning) in Essential 8 Medium (Thermo Fisher Scientific). Differentiation into intestinal organoids was induced as previously reported^100–102^. Briefly, single hPSCs were seeded at a density of 100,000 cells per well in a Matrigel-coated 24-well plate. The base differentiation medium was RPMI 1640 (Thermo Fisher Scientific). When hPSCs reached 90% confluence, they were treated with Activin A (100 ng ml^−1^, R&D Systems) for 3 days, and FBS was gradually increased in the medium to 0%, 0.2%, and 2%. Next, the differentiating cells were treated with FGF-4 (500 ng ml^−1^, R&D Systems) and CHIR99021 (3 µM, Sigma) for 4 days with 2% FBS. When mid-hindgut spheroids developed, they were transferred into Matrigel or IEM hydrogel and cultured with human intestinal organoid medium: advanced DMEM/F12 supplemented with 2 mM l-glutamine (Thermo Fisher Scientific), HEPES, P/S, 1× B27, 500 ng ml^−1^ human R-Spondin1 (R&D Systems), 100 ng ml^−1^ human Noggin (R&D Systems), and 50 ng ml^−1^ human EGF (R&D Systems). The viability of the hPSC-derived intestinal organoids encapsulated in IEM hydrogel or Matrigel was evaluated using a Live/Dead viability/cytotoxicity kit (Thermo Fisher Scientific). Live/Dead staining was performed for 30 min, and organoids were imaged using a fluorescence microscope (IX71, Olympus, Tokyo, Japan). The expression of intestinal marker proteins in the hiPSC-derived intestinal organoids was also visualized with immunocytochemical staining of the organoids.

### Tumoroid formation

Human colon cancer cell lines (DLD-1, HT29) and human gastric cancer cell lines (MKN-74, NCI-N87) were cultured in DMEM containing 10% (v/v) FBS and 100 U/ml P/S (all from Thermo Fisher Scientific). Cells were suspended in pre-gel solutions of Matrigel (Corning), SEM hydrogel, or IEM hydrogel at a concentration of 5 × 10^5^ cells/ml. After gelation, cells were cultured until the formation of tumoroids. Immunostaining and imaging of tumoroids were conducted in the same way as GI organoid staining.

### Statistics and reproducibility

Statistical analyses and graphical representations of the data from this study were mostly generated using GraphPad Prism 8 (GraphPad Software, La Jolla, CA, USA). Results are presented as mean ± S.D. The unpaired, two-sided Student’s *t*-tests with 95% and 99% confidence intervals was used to determine the significance of the data between the two groups. One-way analysis of variance was conducted to determine the significance of data with more than two groups and was followed by Tukey’s multiple comparisons test. Proteomic data were analyzed using PCA and evaluated by computing two-tailed Pearson’s correlation coefficient (*r*) with 95% confidence intervals. Both tests were performed using GraphPad Prism 9.1.1 (GraphPad software). No statistical method was used to predetermine the sample size. Throughout the study, the sample size was determined based on our preliminary studies and on the criteria in the field. At least three biological samples were included for one experiment and one to three independent experiments were performed to ensure sufficient reproducibility of the results. Biological replicates (*N*) and the numbers of the independent experiments are indicated in each figure legend.

### Reporting summary

Further information on research design is available in the Nature Research Reporting Summary linked to this article.

## Supplementary information

Supplementary Information

Description of Additional Supplementary Files

Dataset 1

Reporting Summary

## Data Availability

Proteomics data are available at the ProteomeXchange Consortium via the PRIDE partner repository with the dataset identifier “PXD023694 [proteomecentral.proteomexchange.org/cgi/GetDataset?ID=PXD023694]” and “PXD023705 [proteomecentral.proteomexchange.org/cgi/GetDataset?ID=PXD023705]”. RNA-sequencing data have been deposited to the Gene Expression Omnibus (GEO) public repository under accession codes “GSE165309”. The protein samples were identified by MS/MS data of peptides against the Mouse UniProt database (2020.03 release) for Matrigel and Sus scrofa (pig) UniProt database (2020.03 release) for “porcine intestine and stomach tissues [ftp.uniprot.org/pub/databases/uniprot/previous_major_releases/release-2020_03/uniref/]”. Proteins identified in Matrigel and tissue extracellular matrix hydrogels were compared with the datasets in the “Human Protein Atlas [www.proteinatlas.org]” and “Matrisome Project [matrisomeproject.mit.edu/proteins/]”. Raw data for all figures are provided as source data and the lists of total proteins detected by proteomic analysis are provided as supplementary data. All microscopic images and other data generated for this study are available from the corresponding author on reasonable request. Source data are provided with this paper.

## Source data

Source Data

## References

[1] Sato T (2009). Single Lgr5 stem cells build crypt-villus structures in vitro without a mesenchymal niche. Nature.

[2] Barker N (2010). Lgr5(+ve) stem cells drive self-renewal in the stomach and build long-lived gastric units in vitro. Cell Stem Cell.

[3] Sato T (2011). Long-term expansion of epithelial organoids from human colon, adenoma, adenocarcinoma, and Barrett’s epithelium. Gastroenterology.

[4] Jung P (2011). Isolation and in vitro expansion of human colonic stem cells. Nat. Med..

[5] Huch M (2013). In vitro expansion of single Lgr5+ liver stem cells induced by Wnt-driven regeneration. Nature.

[6] Peterson NC (2008). From bench to cageside: Risk assessment for rodent pathogen contamination of cells and biologics. ILAR J..

[7] Liu H (2011). Removal of lactate dehydrogenase-elevating virus from human-in-mouse breast tumor xenografts by cell-sorting. J. Virol. Methods.

[8] Riley V (1978). The LDH virus: an interfering biological contaminant. Science.

[9] Ammann CG, Messer RJ, Peterson KE, Hasenkrug KJ (2009). Lactate dehydrogenase-elevating virus induces systemic lymphocyte activation via TLR7-dependent IFNalpha responses by plasmacytoid dendritic cells. PLoS ONE.

[10] Hughes CS, Postovit LM, Lajoie GA (2010). Matrigel: a complex protein mixture required for optimal growth of cell culture. Proteomics.

[11] Benton G, Arnaoutova I, George J, Kleinman HK, Koblinski J (2014). Matrigel: from discovery and ECM mimicry to assays and models for cancer research. Adv. Drug Deliv. Rev..

[12] Henke E, Nandigama R, Ergun S (2019). Extracellular Matrix in the tumor microenvironment and its impact on cancer therapy. Front. Mol. Biosci..

[13] Gjorevski N (2016). Designer matrices for intestinal stem cell and organoid culture. Nature.

[14] Cruz-Acuna R (2017). Synthetic hydrogels for human intestinal organoid generation and colonic wound repair. Nat. Cell Biol..

[15] Broguiere N (2018). Growth of epithelial organoids in a defined hydrogel. Adv. Mater..

[16] Capeling MM (2019). Nonadhesive alginate hydrogels support growth of pluripotent stem cell-derived intestinal organoids. Stem Cell Rep..

[17] Lee JS (2019). Tissue beads: tissue-specific extracellular matrix microbeads to potentiate reprogrammed cell-based therapy. Adv. Funct. Mater..

[18] Pati F (2014). Printing three-dimensional tissue analogues with decellularized extracellular matrix bioink. Nat. Commun..

[19] Totonelli G (2012). A rat decellularized small bowel scaffold that preserves villus-crypt architecture for intestinal regeneration. Biomaterials.

[20] Giobbe GG (2019). Extracellular matrix hydrogel derived from decellularized tissues enables endodermal organoid culture. Nat. Commun..

[21] Jin Y (2018). Three-dimensional brain-like microenvironments facilitate the direct reprogramming of fibroblasts into therapeutic neurons. Nat. Biomed. Eng..

[22] Kloxin AM, Benton JA, Anseth KS (2010). In situ elasticity modulation with dynamic substrates to direct cell phenotype. Biomaterials.

[23] Islam MM (2018). Biomaterials-enabled cornea regeneration in patients at high risk for rejection of donor tissue transplantation. NPJ Regen. Med..

[24] Frantz C, Stewart KM, Weaver VM (2010). The extracellular matrix at a glance. J. Cell Sci..

[25] Oshiro M (2001). Immunohistochemical localization of heparan sulfate proteoglycan in human gastrointestinal tract. Histochem. Cell Biol..

[26] Yamamoto S (2013). Heparan sulfate on intestinal epithelial cells plays a critical role in intestinal crypt homeostasis via Wnt/beta-catenin signaling. Am. J. Physiol. Gastrointest. Liver Physiol..

[27] Katakam SK (2021). The heparan sulfate proteoglycan syndecan-1 regulates colon cancer stem cell function via a focal adhesion kinase-Wnt signaling axis. FEBS J..

[28] Han W (2019). Directed differential behaviors of multipotent adult stem cells from decellularized tissue/organ extracellular matrix bioinks. Biomaterials.

[29] De Santis, M. M., Bolukbas, D. A., Lindstedt, S. & Wagner, D. E. How to build a lung: latest advances and emerging themes in lung bioengineering. *Eur. Respir. J*. **52**, 1601355 (2018). 10.1183/13993003.01355-2016 29903859

[30] Schumacher MA (2015). The use of murine-derived fundic organoids in studies of gastric physiology. J. Physiol..

[31] Aoyama F, Sawaguchi A (2011). Functional transformation of gastric parietal cells and intracellular trafficking of ion channels/transporters in the apical canalicular membrane associated with acid secretion. Biol. Pharm. Bull..

[32] Schroeder BO (2019). Fight them or feed them: how the intestinal mucus layer manages the gut microbiota. Gastroenterol. Rep..

[33] Boccellato F (2019). Polarised epithelial monolayers of the gastric mucosa reveal insights into mucosal homeostasis and defence against infection. Gut.

[34] Sontheimer-Phelps A (2020). Human Colon-on-a-Chip Enables Continuous In Vitro Analysis of Colon Mucus Layer Accumulation and Physiology. Cell Mol. Gastroenterol. Hepatol..

[35] Sun L, Yang H, Chen M, Ma D, Lin C (2013). RNA-Seq reveals dynamic changes of gene expression in key stages of intestine regeneration in the sea cucumber *Apostichopus japonicus*. PLoS ONE.

[36] Meran L, Baulies A, Li VSW (2017). Intestinal stem cell niche: the extracellular matrix and cellular components. Stem Cells Int..

[37] Beumer J (2020). High-resolution mRNA and secretome atlas of human enteroendocrine cells. Cell.

[38] Gehart H (2019). Identification of enteroendocrine regulators by real-time single-cell differentiation mapping. Cell.

[39] Yui S (2018). YAP/TAZ-dependent reprogramming of colonic epithelium links ECM remodeling to tissue regeneration. Cell Stem Cell.

[40] Qu M (2021). Establishment of intestinal organoid cultures modeling injury-associated epithelial regeneration. Cell Res..

[41] Jarde T (2020). Mesenchymal niche-derived neuregulin-1 drives intestinal stem cell proliferation and regeneration of damaged epithelium. Cell Stem Cell.

[42] Iizuka M, Konno S (2011). Wound healing of intestinal epithelial cells. World J. Gastroenterol..

[43] Sturm A, Dignass AU (2008). Epithelial restitution and wound healing in inflammatory bowel disease. World J. Gastroenterol..

[44] Groulx JF (2011). Collagen VI is a basement membrane component that regulates epithelial cell-fibronectin interactions. Matrix Biol..

[45] Benoit YD, Groulx JF, Gagne D, Beaulieu JF (2012). RGD-dependent epithelial cell-matrix interactions in the human intestinal crypt. J. Signal Transduct..

[46] Moreira AM (2020). The extracellular matrix: an accomplice in gastric cancer development and progression. Cells.

[47] Simo P (1991). Changes in the expression of laminin during intestinal development. Development.

[48] Virtanen I (1995). Differential expression of laminin chains and their integrin receptors in human gastric-mucosa. Am. J. Pathol..

[49] Kurtz A, Oh SJ (2012). Age related changes of the extracellular matrix and stem cell maintenance. Prev. Med..

[50] Gilpin SE (2017). Fibrillin-2 and Tenascin-C bridge the age gap in lung epithelial regeneration. Biomaterials.

[51] Lukjanenko L (2016). Loss of fibronectin from the aged stem cell niche affects the regenerative capacity of skeletal muscle in mice. Nat. Med..

[52] Du L, Betti M (2016). Identification and evaluation of cryoprotective peptides from chicken collagen: Ice-Growth inhibition activity compared to that of type I antifreeze proteins in sucrose model systems. J. Agric. Food Chem..

[53] Turner RA, Mendel G, Wauthier E, Barbier C, Reid LM (2012). Hyaluronan-supplemented buffers preserve adhesion mechanisms facilitating cryopreservation of human hepatic stem/progenitor cells. Cell Transpl..

[54] Gurruchaga H (2018). Low molecular-weight hyaluronan as a cryoprotectant for the storage of microencapsulated cells. Int. J. Pharm..

[55] Motoike S (2018). Cryopreserved clumps of mesenchymal stem cell/extracellular matrix complexes retain osteogenic capacity and induce bone regeneration. Stem Cell Res. Ther..

[56] Miyamoto Y, Enosawa S, Takeuchi T, Takezawa T (2009). Cryopreservation in situ of cell monolayers on collagen vitrigel membrane culture substrata: ready-to-use preparation of primary hepatocytes and ES cells. Cell Transpl..

[57] Jin Y (2018). Vascularized liver organoids generated using induced hepatic tissue and dynamic liver-specific microenvironment as a drug testing platform. Adv. Funct. Mater..

[58] Aisenbrey EA, Murphy WL (2020). Synthetic alternatives to Matrigel. Nat. Rev. Mater..

[59] Schenke-Layland K (2003). Impact of decellularization of xenogeneic tissue on extracellular matrix integrity for tissue engineering of heart valves. J. Struct. Biol..

[60] Lehr EJ (2011). Decellularization reduces immunogenicity of sheep pulmonary artery vascular patches. J. Thorac. Cardiovasc. Surg..

[61] Yui SR (2012). Functional engraftment of colon epithelium expanded in vitro from a single adult Lgr5(+) stem cell. Nat. Med..

[62] Fordham RP (2013). Transplantation of expanded fetal intestinal progenitors contributes to colon regeneration after injury. Cell Stem Cell.

[63] Kim S (2022). Intestinal extracellular matrix hydrogels to generate intestinal organoids for translational applications. J. Ind. Eng. Chem..

[64] Uriel S (2009). Extraction and assembly of tissue-derived gels for cell culture and tissue engineering. Tissue Eng. Part C Methods.

[65] Durbeej M (2010). Laminins. Cell Tissue Res..

[66] Teller IC (2007). Laminins in the developing and adult human small intestine: relation with the functional absorptive unit. Dev. Dyn..

[67] Uhlen M (2015). Proteomics. Tissue-based map of the human proteome. Science.

[68] O’Connell FC, Martin F (2000). Laminin-rich extracellular matrix association with mammary epithelial cells suppresses Brca1 expression. Cell Death Differ..

[69] Robinson KA (2017). Decorin and biglycan are necessary for maintaining collagen fibril structure, fiber realignment, and mechanical properties of mature tendons. Matrix Biol..

[70] Iozzo RV, Moscatello DK, McQuillan DJ, Eichstetter I (1999). Decorin is a biological ligand for the epidermal growth factor receptor. J. Biol. Chem..

[71] Bi XL (2008). Genetic deficiency of decorin causes intestinal tumor formation through disruption of intestinal cell maturation. Carcinogenesis.

[72] Schonherr E, Lugering N, Stoll R, Domschke W, Kresse H (1997). Differences in decorin and biglycan expression in patients with gastric ulcer healing. Scand. J. Gastroenterol..

[73] Lohr K (2012). Extracellular matrix protein lumican regulates inflammation in a mouse model of colitis. Inflamm. Bowel Dis..

[74] Jabaji Z (2013). Use of collagen gel as an alternative extracellular matrix for the in vitro and in vivo growth of murine small intestinal epithelium. Tissue Eng. Part C Methods.

[75] Jee JH (2019). Development of collagen-based 3D matrix for gastrointestinal tract-derived organoid culture. Stem Cells Int..

[76] Keane TJ (2015). Tissue-specific effects of esophageal extracellular matrix. Tissue Eng. Part A.

[77] Reing JE (2010). The effects of processing methods upon mechanical and biologic properties of porcine dermal extracellular matrix scaffolds. Biomaterials.

[78] Choi YS (2021). Immunomodulatory scaffolds derived from lymph node extracellular matrices. ACS Appl. Mater. Interfaces.

[79] Jin, Y. et al. Reconstruction of muscle fascicle-like tissues by anisotropic 3D patterning. *Adv. Funct. Mater.* **31**, 2006227 (2021).

[80] Lee JS (2014). Liver extracellular matrix providing dual functions of two-dimensional substrate coating and three-dimensional injectable hydrogel platform for liver tissue engineering. Biomacromolecules.

[81] Mun DG (2019). Proteogenomic characterization of human early-onset gastric cancer. Cancer Cell.

[82] Cox J, Mann M (2008). MaxQuant enables high peptide identification rates, individualized p.p.b.-range mass accuracies and proteome-wide protein quantification. Nat. Biotechnol..

[83] Cox J (2011). Andromeda: a peptide search engine integrated into the MaxQuant environment. J. Proteome Res..

[84] Lee JH (2020). Proteomic analysis of human synovial fluid reveals potential diagnostic biomarkers for ankylosing spondylitis. Clin. Proteom..

[85] Schwanhausser B (2011). Global quantification of mammalian gene expression control. Nature.

[86] Tyanova S (2016). The Perseus computational platform for comprehensive analysis of (prote)omics data. Nat. Methods.

[87] Naba A (2016). The extracellular matrix: tools and insights for the “omics” era. Matrix Biol..

[88] Raudvere U (2019). g:Profiler: a web server for functional enrichment analysis and conversions of gene lists. Nucleic Acids Res..

[89] Fagerberg L (2014). Analysis of the human tissue-specific expression by genome-wide integration of transcriptomics and antibody-based proteomics. Mol. Cell Proteom..

[90] Gremel G (2015). The human gastrointestinal tract-specific transcriptome and proteome as defined by RNA sequencing and antibody-based profiling. J. Gastroenterol..

[91] Mi H, Muruganujan A, Ebert D, Huang X, Thomas PD (2019). PANTHER version 14: more genomes, a new PANTHER GO-slim and improvements in enrichment analysis tools. Nucleic Acids Res..

[92] Ashburner M (2000). Gene ontology: tool for the unification of biology. The Gene Ontology Consortium. Nat. Genet..

[93] Gene Ontology C (2021). The Gene Ontology resource: enriching a GOld mine. Nucleic Acids Res..

[94] Ghosh S, Chan CK (2016). Analysis of RNA-Seq Data Using TopHat and Cufflinks. Methods Mol. Biol..

[95] Kim SH (2017). Anisotropically organized three-dimensional culture platform for reconstruction of a hippocampal neural network. Nat. Commun..

[96] Engevik AC (2016). The development of spasmolytic polypeptide/TFF2-expressing metaplasia (SPEM) during gastric repair is absent in the aged stomach. Cell Mol. Gastroenterol. Hepatol..

[97] Owen CR, Yuan L, Basson MD (2008). Smad3 knockout mice exhibit impaired intestinal mucosal healing. Lab. Invest..

[98] Kovalenko PL, Kunovska L, Chen J, Gallo KA, Basson MD (2012). Loss of MLK3 signaling impedes ulcer healing by modulating MAPK signaling in mouse intestinal mucosa. Am. J. Physiol. Gastrointest. Liver Physiol..

[99] Wang Q, More SK, Vomhof-DeKrey EE, Golovko MY, Basson MD (2019). Small molecule FAK activator promotes human intestinal epithelial monolayer wound closure and mouse ulcer healing. Sci. Rep..

[100] Spence JR (2011). Directed differentiation of human pluripotent stem cells into intestinal tissue in vitro. Nature.

[101] Watson CL (2014). An in vivo model of human small intestine using pluripotent stem cells. Nat. Med..

[102] Workman MJ (2017). Engineered human pluripotent-stem-cell-derived intestinal tissues with a functional enteric nervous system. Nat. Med..

