# Supplementary material for: Tissue extracellular matrix hydrogels as alternatives to Matrigel for culturing gastrointestinal organoids
Source: Nat Commun. 2022 Mar 30;13:1692. doi: 10.1038/s41467-022-29279-4 (PMC8967832; doi:10.1038/s41467-022-29279-4)
Supplement: Supplementary file 1 — Supplementary Information [file 41467_2022_29279_MOESM1_ESM.pdf]

Supplementary Information

Tissue extracellular matrix hydrogels as alternatives to Matrigel for culturing gastrointestinal organoids

Suran Kim[1#], Sungjin Min[1#], Yi Sun Choi[1], Sung-Hyun Jo[2], Jae Hun Jung[3], Kyusun Han[3], Jin Kim[1], Soohwan An[1], Yong Woo Ji[3,4], Yun-Gon Kim[2] and Seung-Woo Cho[1,5,6]*

[1]Department of Biotechnology, Yonsei University, Seoul 03722, Republic of Korea.

[2]Department of Chemical Engineering, Soongsil University, Seoul 06978, Republic of Korea.

[3]Institute of Vision Research, Department of Ophthalmology, Yonsei University College of Medicine, Seoul 06229, Republic of Korea.

[4]Department of Ophthalmology, National Health Insurance Service Ilsan Hospital, Goyang 10444, Republic of Korea.

[5]Center for Nanomedicine, Institute for Basic Science (IBS), Seoul 03722, Republic of Korea.

[6]Graduate Program of Nano Biomedical Engineering (NanoBME), Advanced Science Institute, Yonsei University, Seoul 03722, Republic of Korea.

[#]These authors contributed equally to this work.

*Correspondence should be addressed to S.-W. C. (seungwoocho@yonsei.ac.kr)

Inventory of Supplementary Information

Supplementary Figure 1. Protocol optimization for decellularization of GI tissues.

Supplementary Figure 2. Preparation of SEM and IEM hydrogels from decellularized GI tissues.

Supplementary Figure 3. *In vitro* and *in vivo* biological safety evaluation of SEM and IEM hydrogels.

Supplementary Figure 4. Proteomic analysis of decellularized GI tissue-derived matrix and Matrigel.

Supplementary Figure 5. Gene ontology biological process (GOBP) analysis of non-matrisome proteins in Matrigel, SEM, and IEM.

Supplementary Figure 6. List of native tissue-enriched proteins detected in SEM and IEM generated from different tissue batches of the same donor (porcine A1, A2, A3) and different donor batches (porcine A, B, C).

Supplementary Figure 7. Proteomic analysis of decellularized esophagus-derived ECM (EEM) for comparison with SEM, IEM, and Matrigel.

Supplementary Figure 8. Characterization of the mechanical properties of GI tissue-derived ECM hydrogels.

Supplementary Figure 9. GI organoid growth in ECM hydrogels.

Supplementary Figure 10. Quantification of GI marker-positive areas in GI organoids grown in GI tissue-derived ECM hydrogels and Matrigel.

Supplementary Figure 11. Characterization of parietal cells in gastric organoids grown in SEM hydrogel.

Supplementary Figure 12. Comparison of ECM hydrogels derived from whole GI tissues and mucosal tissues.

Supplementary Figure 13. RNA sequencing analysis to confirm the genes upregulated in GI organoids cultured in GI tissue-derived ECM hydrogels.

Supplementary Figure 14. RNA sequencing analysis to compare the gene expression of several subtypes of enteroendocrine cells between intestinal organoids grown in IEM hydrogel and Matrigel.

Supplementary Figure 15. YAP signaling activation in intestinal organoids grown in IEM hydrogel.

Supplementary Figure 16. Tissue-specific effects of ECM hydrogels on GI organoid culture.

Supplementary Figure 17. The effects of IEM hydrogels supplemented with fibronectin (FN) and tenascin (TNC) in intestinal organoid culture.

Supplementary Figure 18. Generation of mouse colonic organoids, human pluripotent stem cell-derived intestinal organoids, and GI tumoroids in GI tissue-derived ECM hydrogels.

Supplementary Figure 19. qPCR analysis of GI organoids cultured in ECM hydrogels after long-term storage.

Supplementary Figure 20. Evaluation of organoid-laden ECM hydrogels after cryopreservation.

Supplementary Figure 21. Microfluidic system for dynamic culture and mass production of GI organoids in ECM hydrogels.

Supplementary Figure 22. Tissue morphology and inflammatory responses in acetic acid-induced GI injury models.

Supplementary Figure 23. Transplantation of TAMRA-SE-labeled ECM hydrogels alone in GI tissues.

Supplementary Figure 24. Transplantation of $EGFP^+$ mouse GI organoids and TAMRA-SE-labeled ECM hydrogels.

Supplementary Table 1. Estimation of the amount and cost of SEM and IEM hydrogels generated from stomach and intestine tissues of one pig.

Supplementary Data. List of total proteins identified from proteomic analysis conducted in this study.

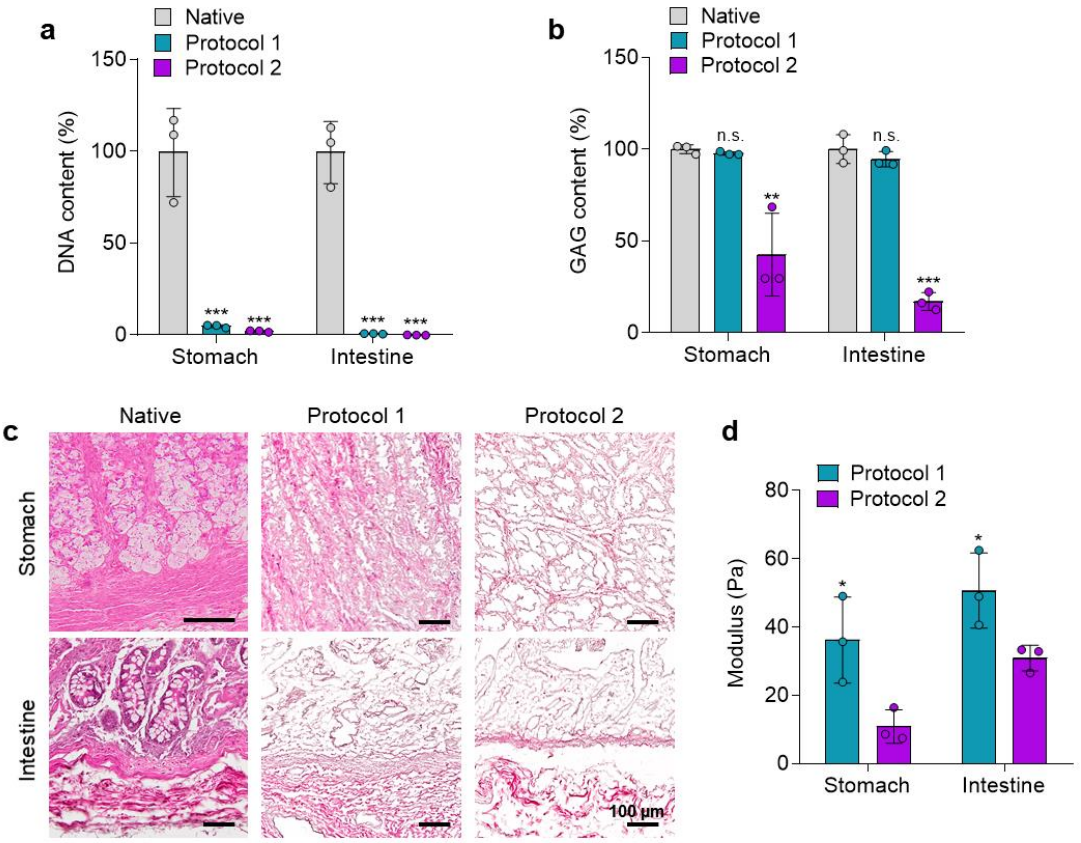

Supplementary Figure 1. Protocol optimization for decellularization of GI tissues. Our protocol using Triton X-100 (Protocol 1) and the conventional protocol using sodium deoxycholate (Protocol 2) were tested for decellularization of porcine stomach and small intestine tissues. (a) DNA content of decellularized porcine stomach and intestinal tissues by protocol 1 and 2 (two-sided student's *t*-test; Native versus Protocol 1, $^{***}p = 0.0004$ for Stomach, $^{***}p < 0.0001$ for Intestine; Native versus Protocol 2, $^{***}p = 0.0003$ for Stomach, $^{***}p < 0.0001$ for Intestine; $N = 3$). (b) glycosaminoglycans (GAG) content of decellularized porcine stomach and intestinal tissues by protocol 1 and 2 (two-sided student's *t*-test; Native versus Protocol 2, $^{**}p = 0.0041$ for Stomach, $^{***}p < 0.0001$ for Intestine; $N = 3$). Non-significant statistical difference was indicated as n.s. ($p > 0.05$). (c) H&E stained images of porcine stomach and intestinal tissues before and after decellularization (scale bar = 100 µm, independent experiment = 1). (d) Elastic modulus of GI tissue-derived hydrogels (5 mg ml^{-1}) prepared from decellularized tissues (two-sided student's *t*-test; Protocol 1 versus Protocol 2, $^{*}p = 0.0313$ for Stomach, $^{*}p = 0.0462$ for Intestine; $N = 3$). The data in (a), (b), and (d) are presented as mean ± S.D.

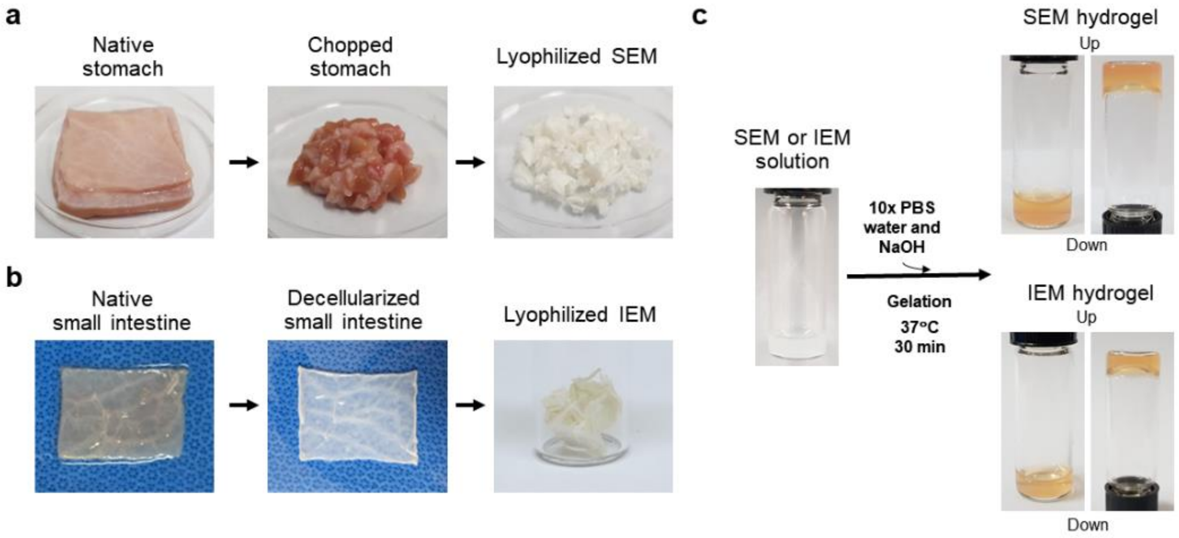

Supplementary Figure 2. Preparation of SEM and IEM hydrogels from decellularized GI tissues. (a, b) Photos showing the procedures to obtain lyophilized tissue from decellularized stomach and intestinal tissues. (c) Generation of SEM and IEM hydrogels with solubilized SEM and IEM acquired by incubation with hydrochloric acid (HCl) solution and pepsin. Pre-gel ECM solutions were prepared with 10× phosphate buffered saline (PBS) and sodium hydroxide (NaOH). Gelation to construct the ECM hydrogels was induced at 37 °C for 30 minutes.

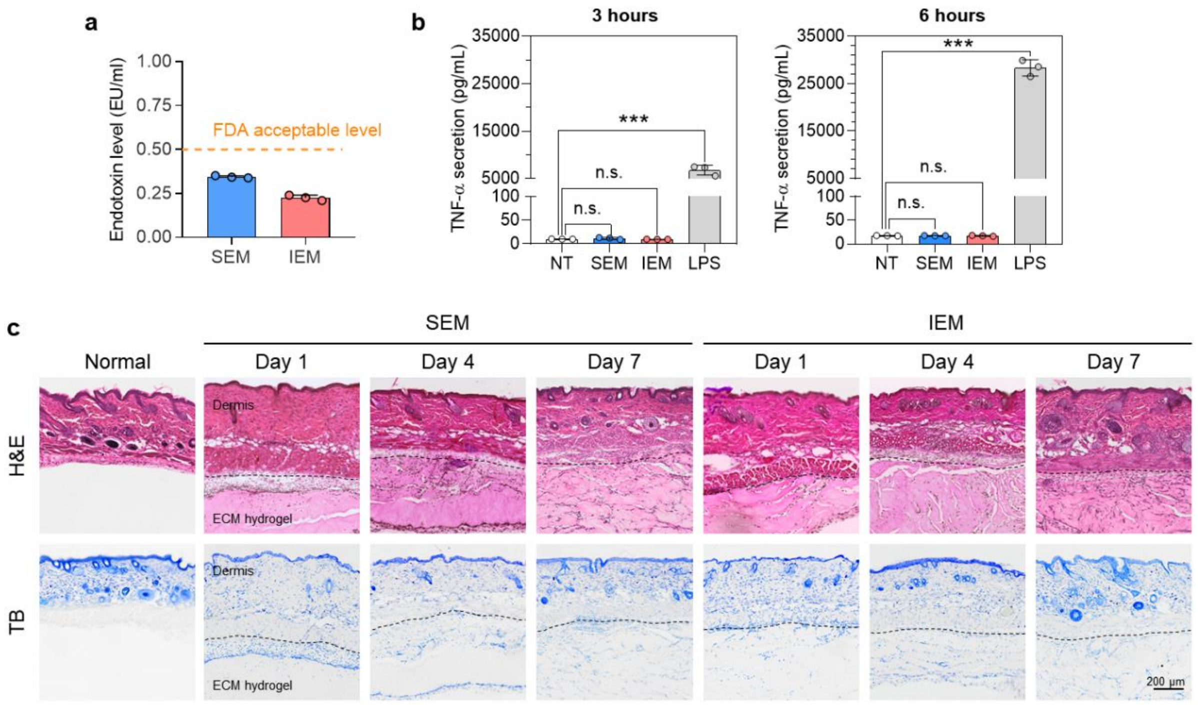

Supplementary Figure 3. ***In vitro*** **and** ***in vivo*** **biological safety evaluation of SEM and IEM hydrogels.** (a) Determination of endotoxin contamination of SEM (5 mg ml^{-1}) and IEM (2 mg ml^{-1}) hydrogels ($N = 3$). FDA acceptable level was indicated with dotted orange line (0.5 EU ml^{-1}). (b) Quantification of TNF-α secretion from RAW 264.7 macrophages cultured with SEM and IEM hydrogels for 3 and 6 hours (one-way ANOVA with Tukey's multiple comparisons test; No treatment (NT) versus LPS, ***$p < 0.0001$; $N = 3$, independent experiments = 2). Lipopolysaccharide (LPS) treatment was applied as a positivie control group. Non-significant statistical difference was indicated as n.s. ($p > 0.05$). (c) H&E and toluidin blue (TB) stained images of mouse skin tissues at day 1, 4, and 7 after subcutaneous injection of SEM and IEM hydrogels (scale bar = 200 μm). Dotted black lines indicate the boundary between dermis and injected ECM hydrogel. Representative images from two independent experiments are shown in (c). The data in (a) and (b) are presented as mean ± S.D.

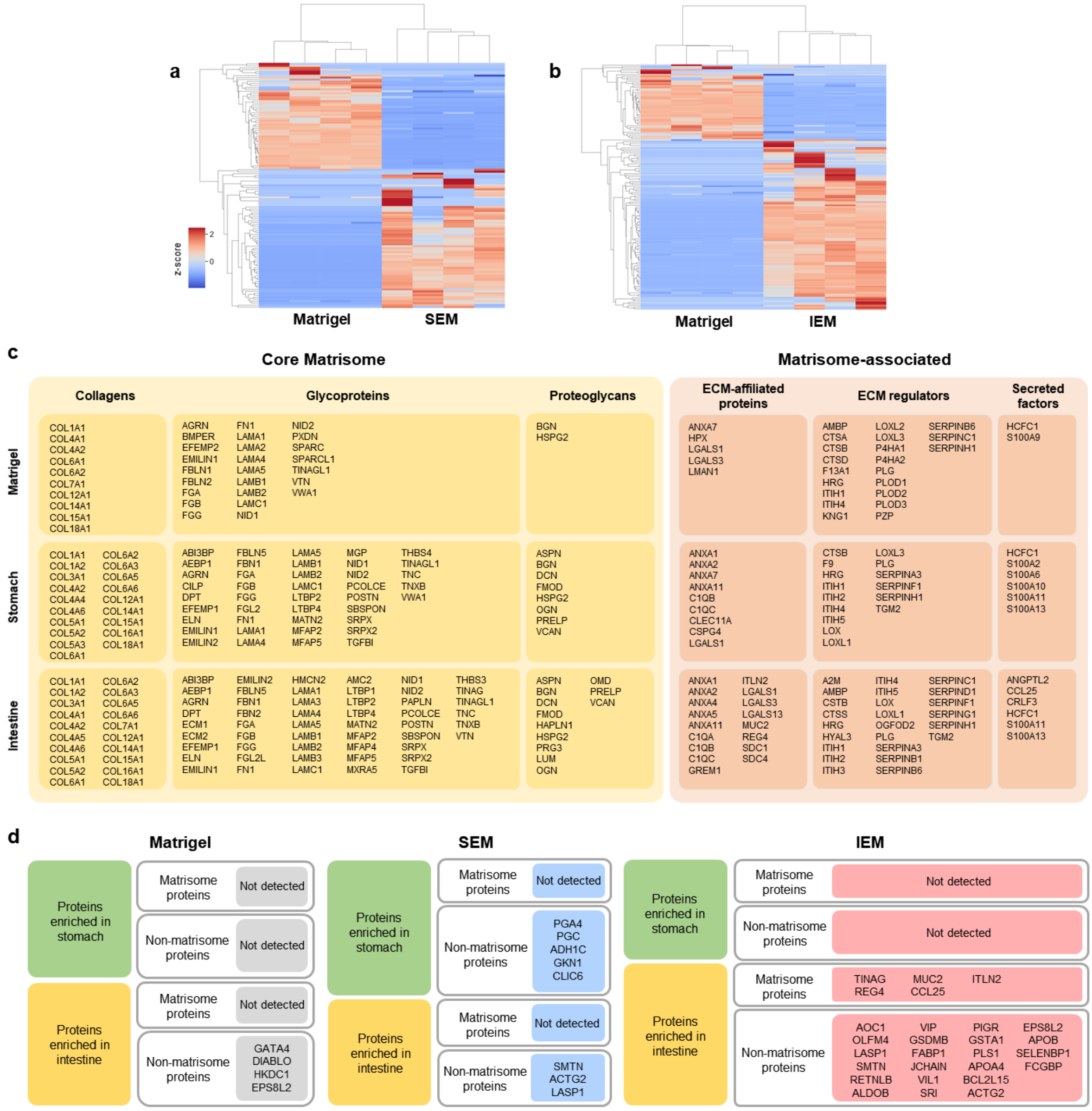

Supplementary Figure 4. Proteomic analysis of decellularized GI tissue-derived matrix and Matrigel. Hierarchical clustering analysis of matrisome proteins in (a) Matrigel versus SEM and (b) Matrigel versus IEM (N = 4). (c) Matrisome protein components contained in Matrigel, SEM, and IEM categorized according to ECM type (N = 4). (d) The matrisome proteins and non-matrisome proteins significantly enriched in native stomach or intestine tissue that were identified in each hydrogel. Averaged values from four biological replicates of each group were used in (d).

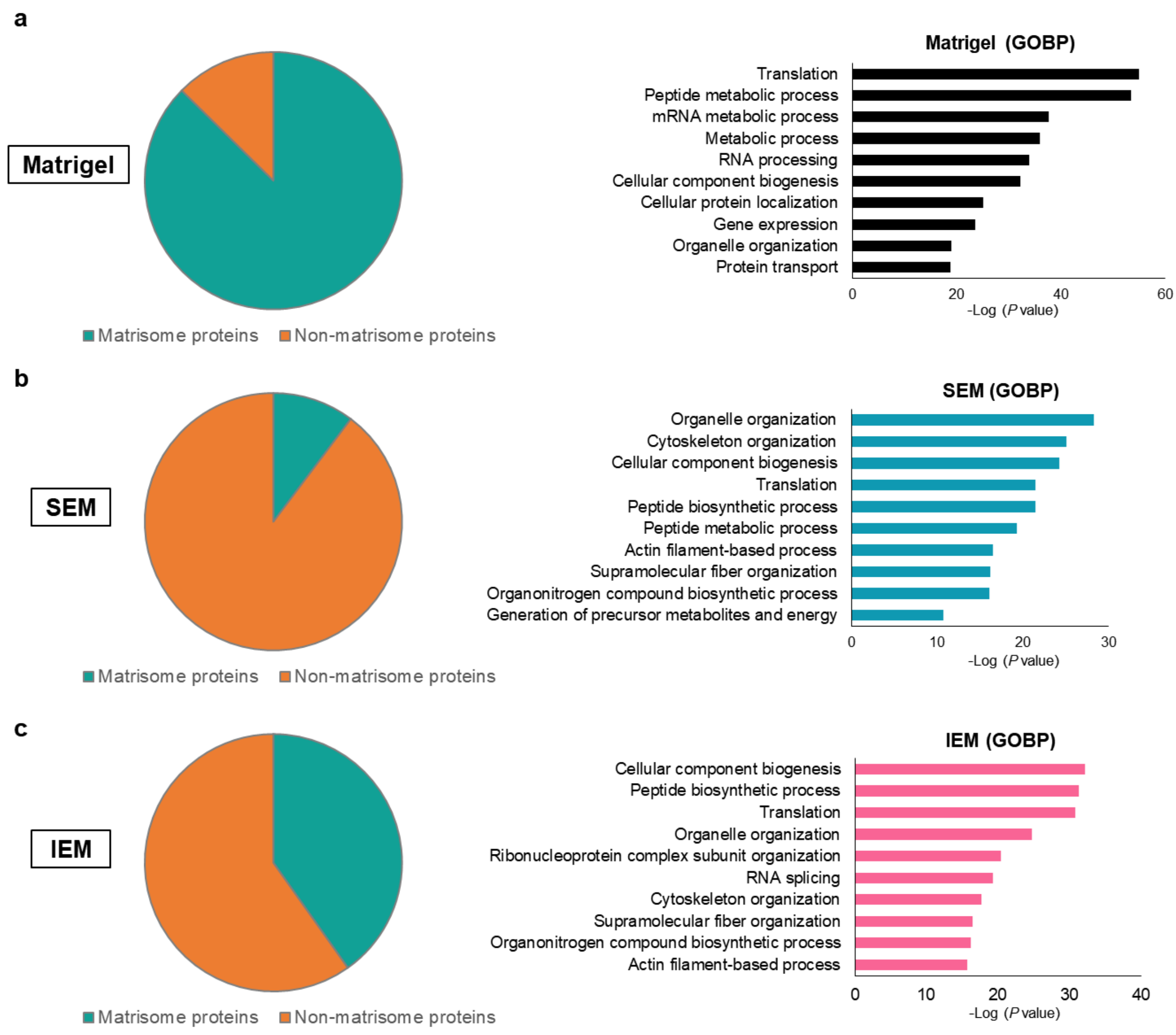

Supplementary Figure 5. Gene ontology biological process (GOBP) analysis of non-matrisome proteins in Matrigel, SEM, and IEM. (a–c) The ratio of matrisome proteins and non-matrisome proteins in Matrigel, SEM, and IEM (left). The top 10 ranked GOBP categories for proteins associated with the non-matrisome component of Matrigel, SEM, and IEM (right). Data in this figure were analyzed from averaged values from four biological replicates.

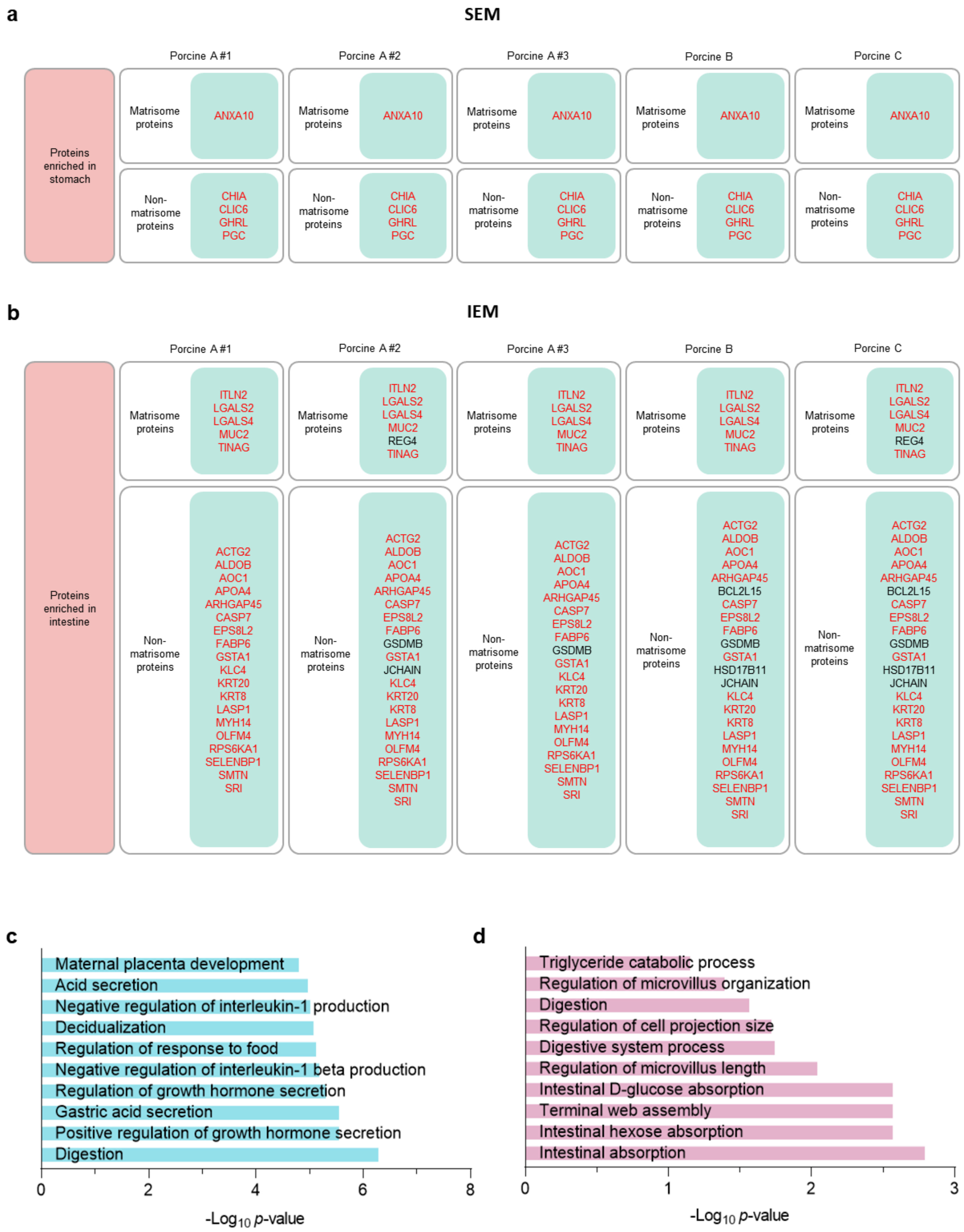

Supplementary Figure 6. List of native tissue-enriched proteins detected in SEM and IEM generated from different tissue batches of the same donor (porcine A1, A2, A3) and different donor batches (porcine A, B, C). Among enriched proteins in native stomach and intestine, the overlapped proteins in five batches of (a) SEM and (b) IEM (porcine A1, A2, A3, B, C) are marked in red. Gene ontology biological process (GOBP) analysis of (c) native stomach-enriched proteins detected in SEM and (d) native intestine-enriched proteins detected in IEM. Data in this figure were analyzed from averaged values from three biological replicates.

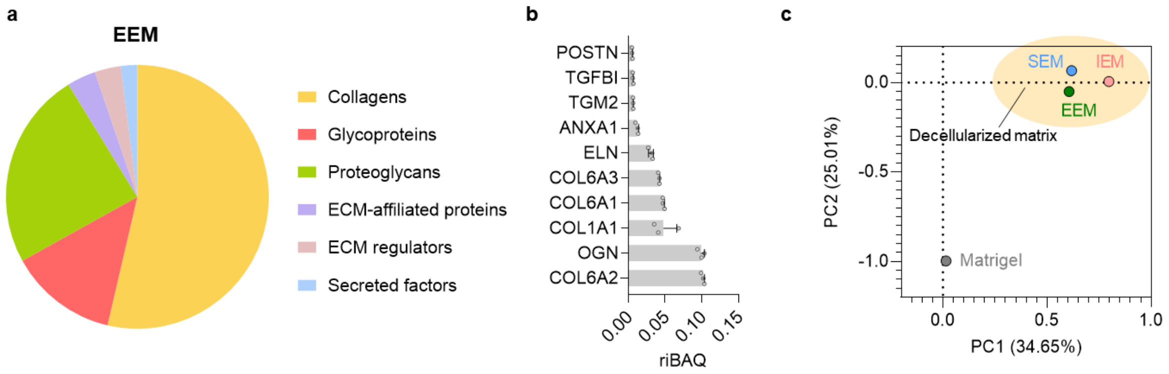

Supplementary Figure 7. Proteomic analysis of decellularized esophagus-derived ECM (EEM) for comparison with SEM, IEM, and Matrigel. (a) The composition of matrisome proteins in EEM and (b) the list of top 10 matrisome proteins detected in EEM. (c) Principal compotent analysis (PCA) of GI tissue-derived ECM (SEM, IEM, and EEM) for comparison with Matrigel. Data in this figure were analyzed from three technical replicates. The data in (b) are presented as mean ± S.D.

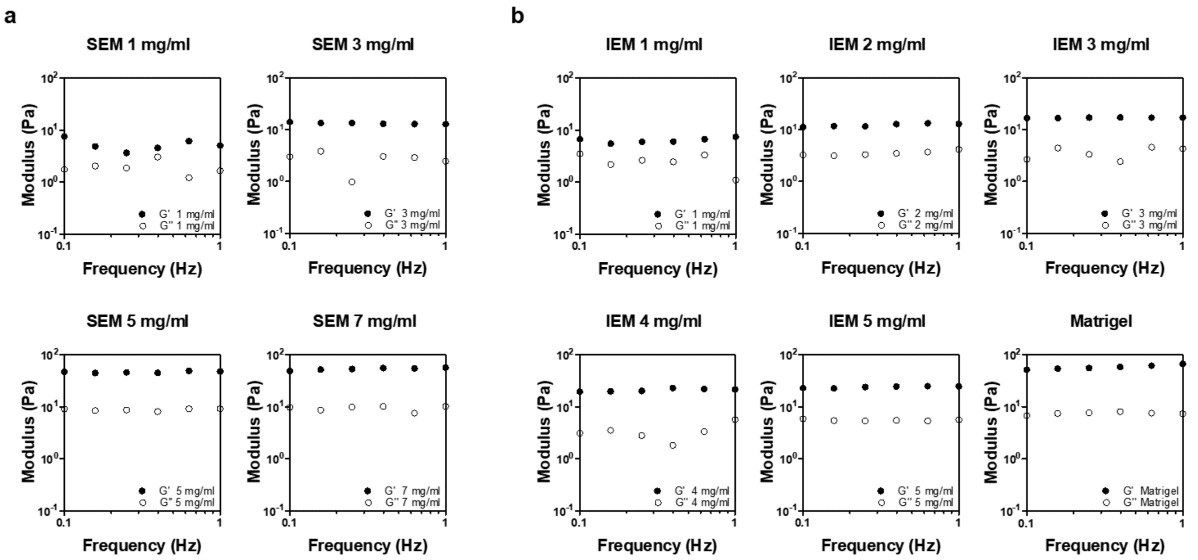

Supplementary Figure 8. Characterization of the mechanical properties of GI tissue-derived ECM hydrogels. Rheological analysis of the storage and loss moduli of (a) SEM and (b) IEM hydrogels produced at different concentrations.

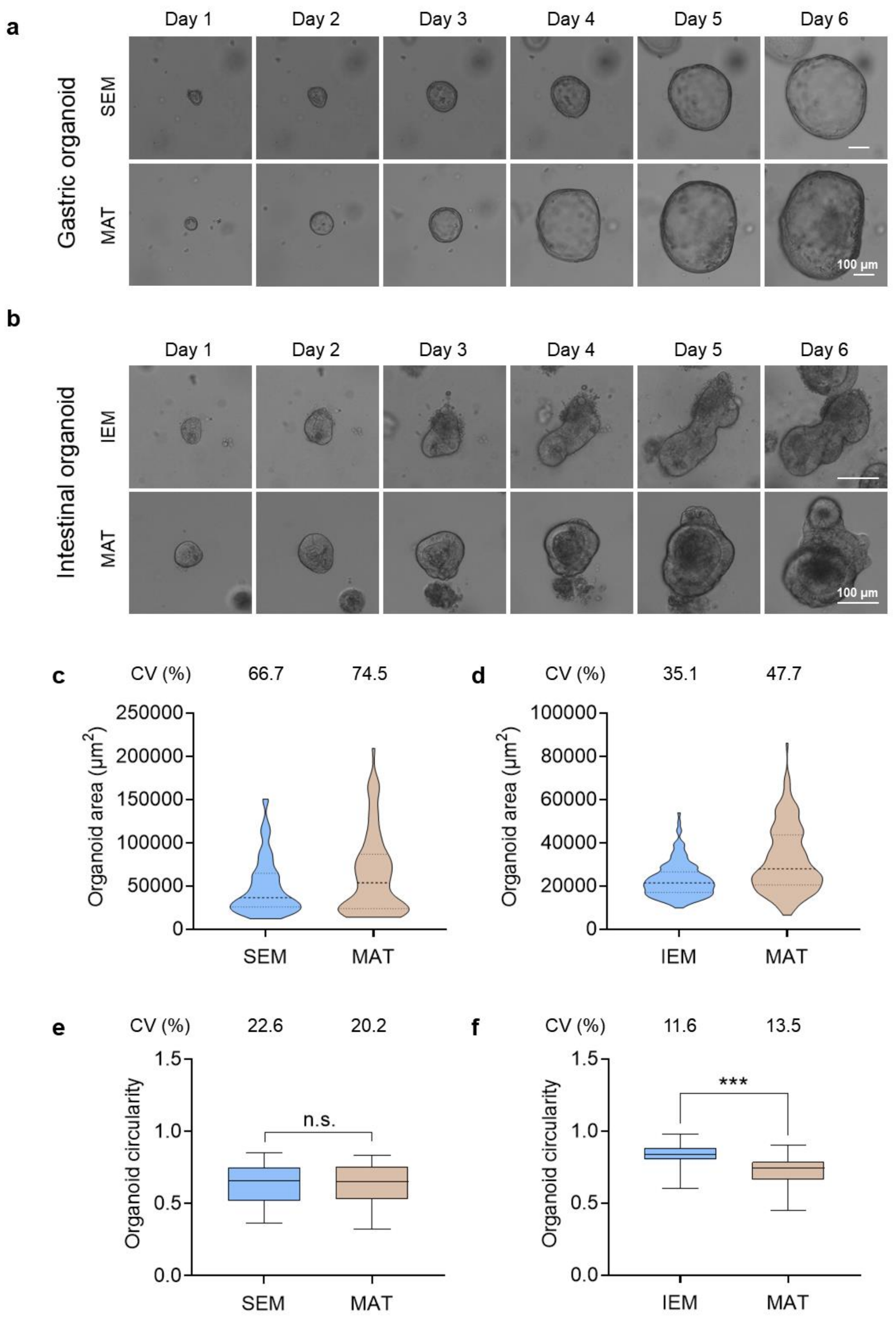

Supplementary Figure 9. GI organoid growth in ECM hydrogels. Brightfield images tracking the growth of (a) gastric and (b) intestinal organoids in GI tissue-derived ECM hydrogels and Matrigel for 6 days (scale bars = 100 μm). Quantification of the projected area of (c) gastric organoids grown in SEM hydrogel and Matrigel (*N* = 74) and (d) intestinal organoids grown in IEM hydrogel and Matrigel (*N* = 146 for IEM and *N* = 147 for MAT). Quantification of the circularity of (e) gastric organoids grown in SEM hydrogel and Matrigel (two-sided student's *t*-test, *N* = 40) and (f) intestinal organoids grown in IEM hydrogel and

Matrigel (two-sided student's t-test; IEM versus MAT, ***$p < 0.0001$; $N = 50$). Non-significant statistical difference was indicated as n.s. ($p > 0.05$). The coefficient of variation (CV) is indicated above each graph in (c)–(f). The centerline of box represents the median and the box represents first (25%) and third (75%) quartiles. The upper bound was determined as 1.5 times the interquartile range (IQR) above the third quaritles, and the lower bound was determined as 1.5 times IQR below the first quartiles. Whiskers show the minimum and maximum of the data between upper and lower bound.

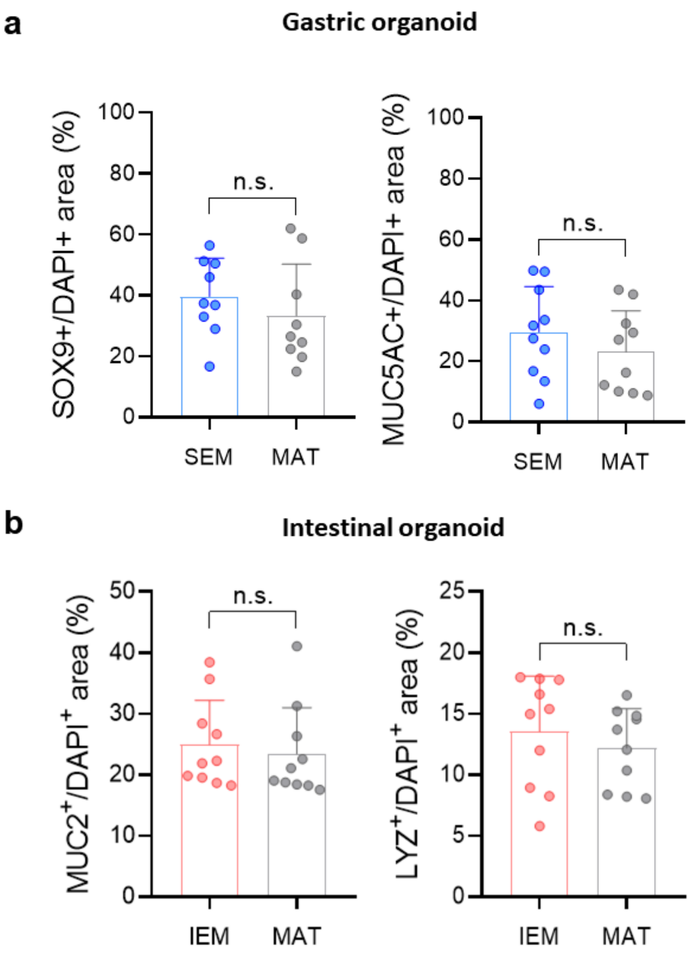

Supplementary Figure 10. Quantification of GI marker-positive areas in GI organoids grown in GI tissue-derived ECM hydrogels and Matrigel. (a) $SOX9^+/DAPI^+$ area (left, $N = 9$) and $MUC5AC^+/DAPI^+$ area (right, $N = 10$) in gastric organoids and (b) $MUC2^+/DAPI^+$ area (left, $N = 10$) and $LYZ^+/DAPI^+$ area (right, $N = 10$) in intestinal organoids grown in each hydrogel. The areas positive for each GI marker in the organoids were quantified from immunofluorescence staining for GI markers. Gastric organoids and intestinal organoids were cultured for 5 days and 6 days, respectively. Statistical significance was determined by two-sided student's *t*-test. Non-significant statistical difference was indicated as n.s. ($p > 0.05$). The data are presented as mean ± S.D.

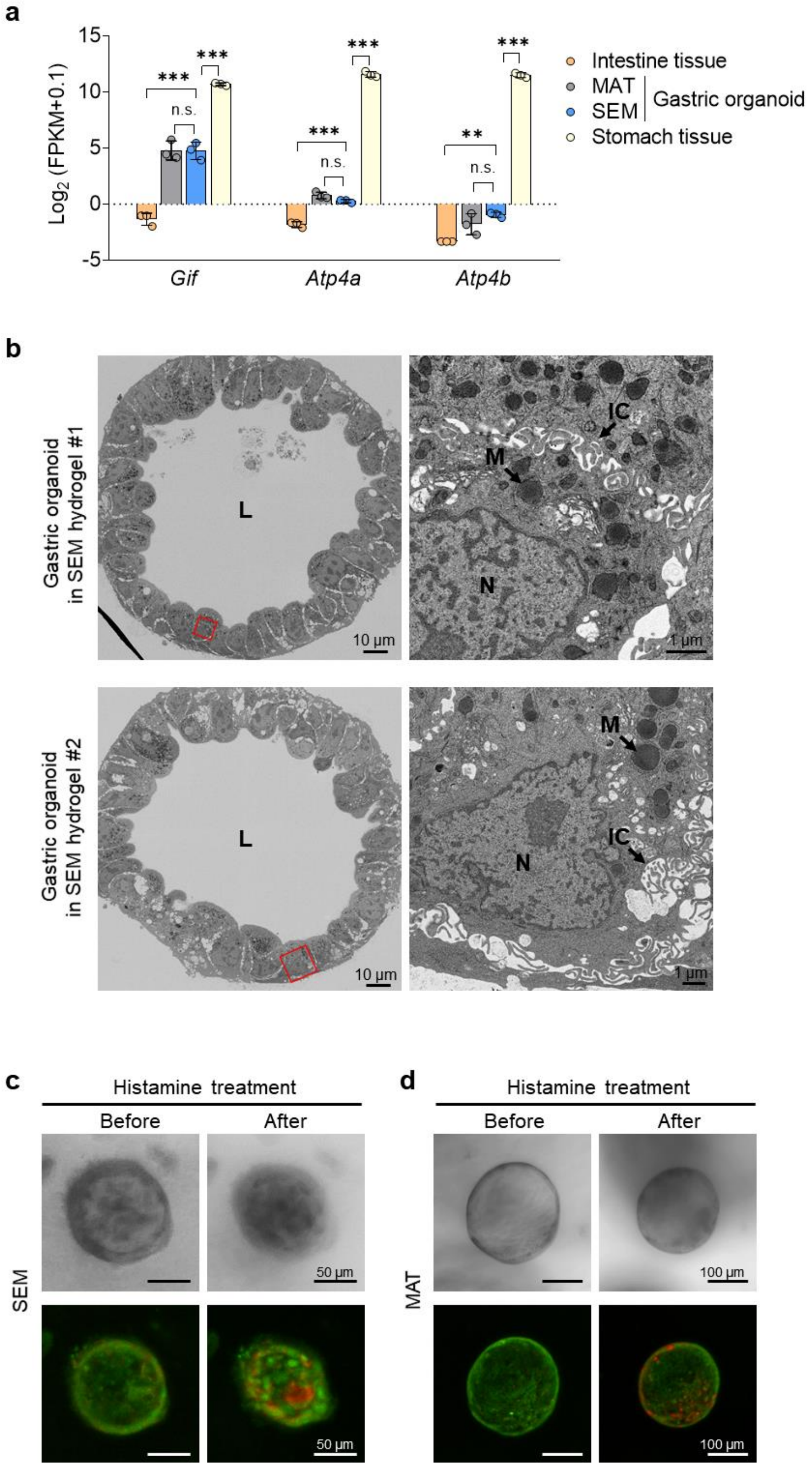

Supplementary Figure 11. Characterization of parietal cells in gastric organoids grown in SEM hydrogel. (a) Comparison of parietal cell-sepcific gene expression (*Gif, Atp4a*, and *Atp4b*) between native GI tissues and gastric organoids grown in SEM hydrogel and Matrigel

(one-way ANOVA with Tukey's multiple comparisons test; SEM versus Intestine tissue, $***p < 0.0001$ for *Gif*, $***p < 0.0001$ for *Atp4a*, $**p = 0.0018$ for *Atp4b*; SEM versus Stomach tissue $***p < 0.0001$ for *Gif*, $***p < 0.0001$ for *Atp4a*, $***p < 0.0001$ for *Atp4b*; $N = 3$). Non-significant statistical difference was indicated as n.s. ($p > 0.05$). (b) The ultrastructure of parietal cells in gastric organoids grown in SEM hydrogel (L, lumen; N, nucleus; IC, intracellular canaliculus; M, mitochondria) (scale bars = 10 μm in left panels and 1 μm in right panels, independent experiment = 1). (c), (d) Functional analysis of gastric organoids. Brightfield images and fluorescence images of gastric organoids in (c) SEM hydrogel and (d) Matrigel before and after treatment of 100 μM histamine for 60 minutes. Gastric organoids were stained with acridine orange to confirm acid secretion (scale bars = 50 μm in (c) and 100 μm in (d)). Representative images from two independent experiments are shown in this figure (c, d). The data in (a) are presented as mean ± S.D.

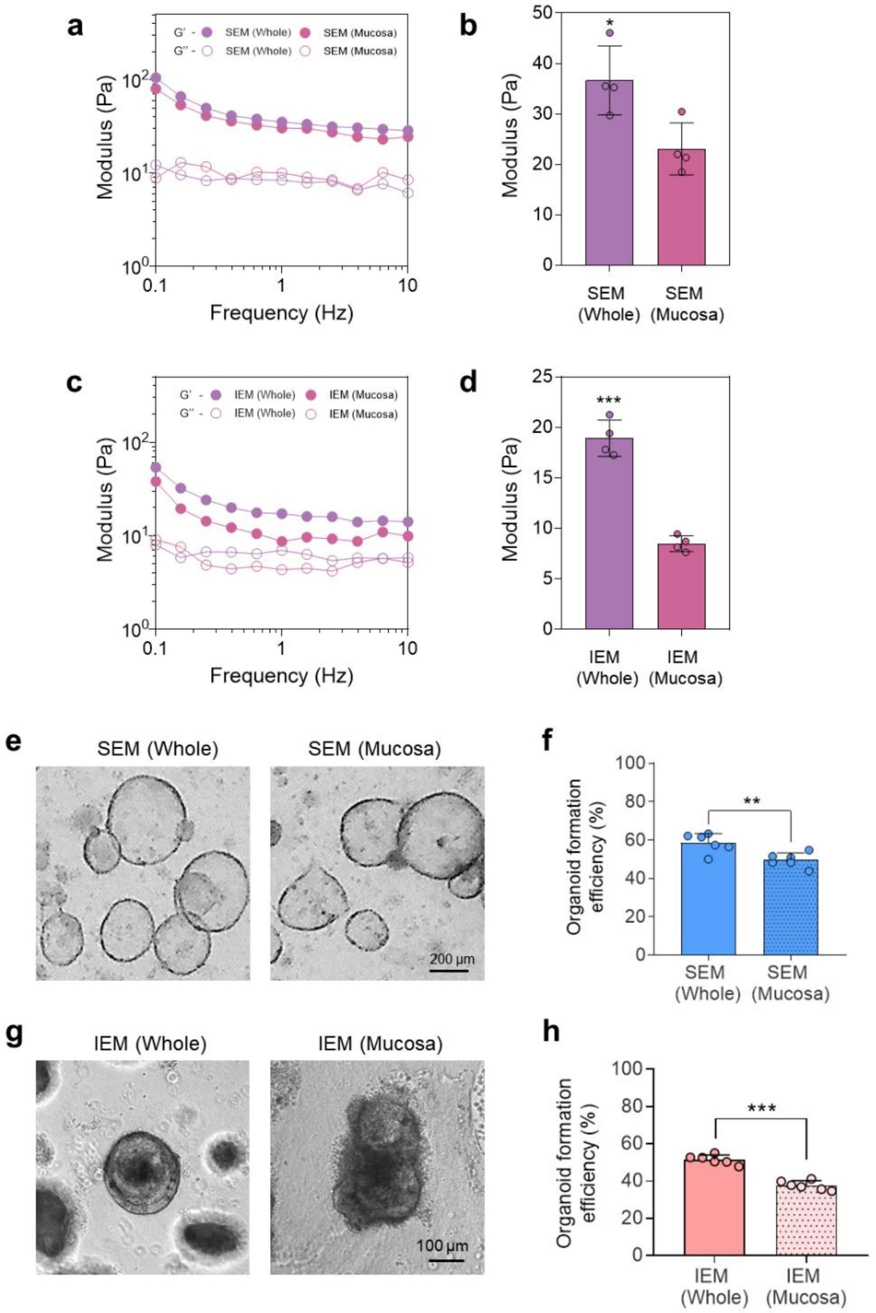

Supplementary Figure 12. Comparison of ECM hydrogels derived from whole GI tissues and mucosal tissues. (a) The storage and loss moduli and (b) the average elastic modulus of SEM hydrogels derived from whole tissue and mucosal tissue of stomach (two-sided student's *t*-test; SEM (Whole) versus SEM (Mucosa), $*p = 0.0193$; $N = 4$). (c) The storage and loss moduli and (d) the average elastic modulus of IEM hydrogels derived from whole tissue and mucosal tissue of small intestine (two-sided student's *t*-test; IEM (Whole) versus IEM (Mucosa), $***p < 0.0001$; $N = 4$). (e) Brightfield images of gastric organoids in each SEM hydrogel (scale bar = 200 μm) and (f) organoid formation efficiency in each SEM hydrogel

(two-sided student's *t*-test; SEM (Whole) versus SEM (Mucosa), $^{**}p = 0.0056$; $N = 6$). (g) Brightfield images of intestinal organoids in each IEM hydrogel (scale bar = 100 μm) and (h) organoid formation efficiency in each IEM hydrogel (two-sided student's *t*-test; IEM (Whole) versus IEM (Mucosa), $^{***}p < 0.0001$; $N = 6$). The data in (b), (d), (f), and (h) are presented as mean ± S.D.

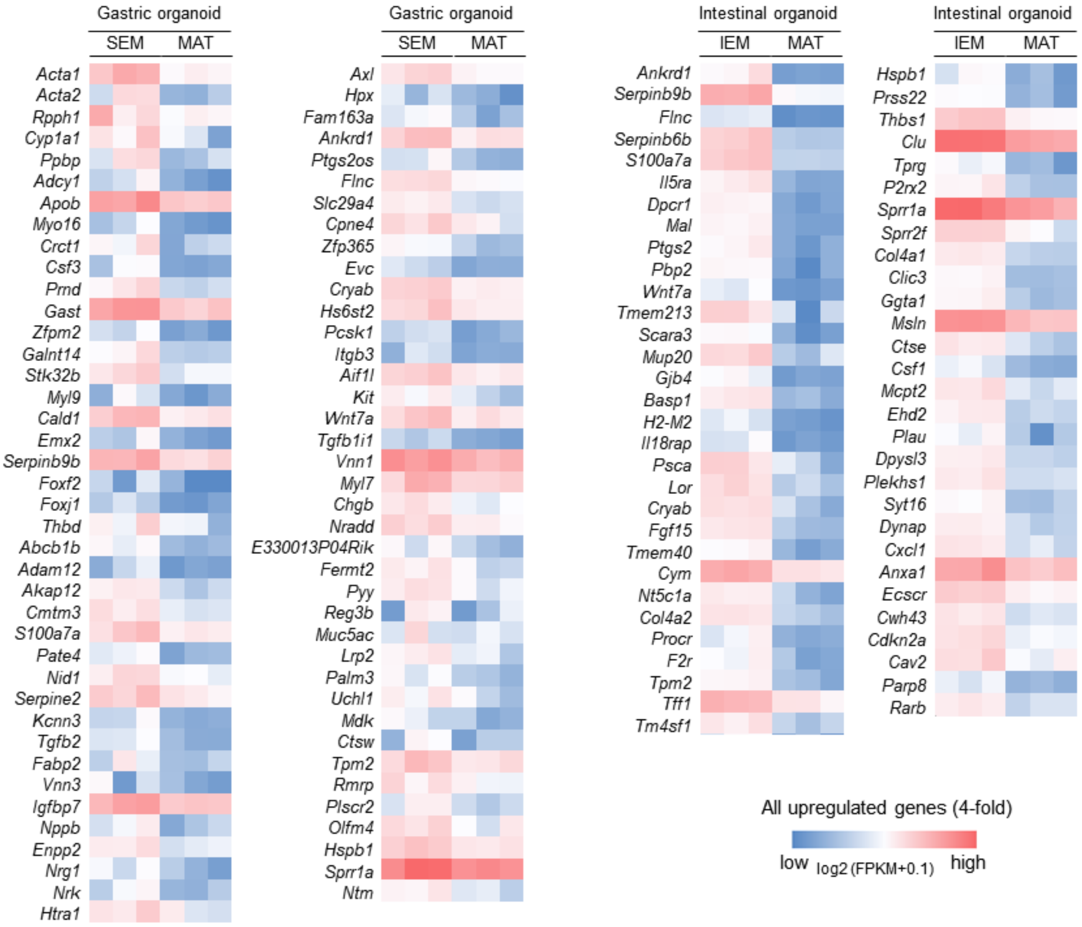

Supplementary Figure 13. RNA sequencing analysis to confirm the genes upregulated in GI organoids cultured in GI tissue-derived ECM hydrogels. Heatmap showing the individual expression levels of genes that were upregulated by more than 4-fold in (a) gastric and (b) intestinal organoids cultured in SEM and IEM hydrogels compared to those cultured in Matrigel (MAT) ($p < 0.05$, FDR < 0.1).

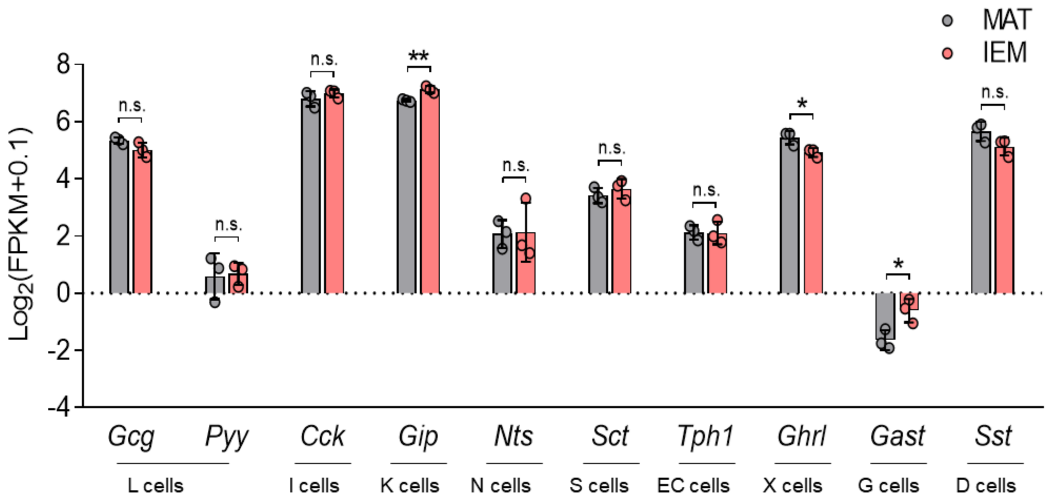

Supplementary Figure 14. RNA sequencing analysis to compare the gene expression of several subtypes of enteroendocrine cells between intestinal organoids grown in IEM hydrogel and Matrigel. The enteroendocrine cell subtypes corresponding to each gene are indicated at the bottom. (two-sided student's *t*-test; MAT versus IEM, ***p* = 0.0061 for *Gip*, **p* = 0.0329 for *Ghrl*, **p* = 0.0291 for *Gast*; *N* = 3). Non-significant statistical difference was indicated as n.s. ($p > 0.05$). The data are presented as mean ± S.D.

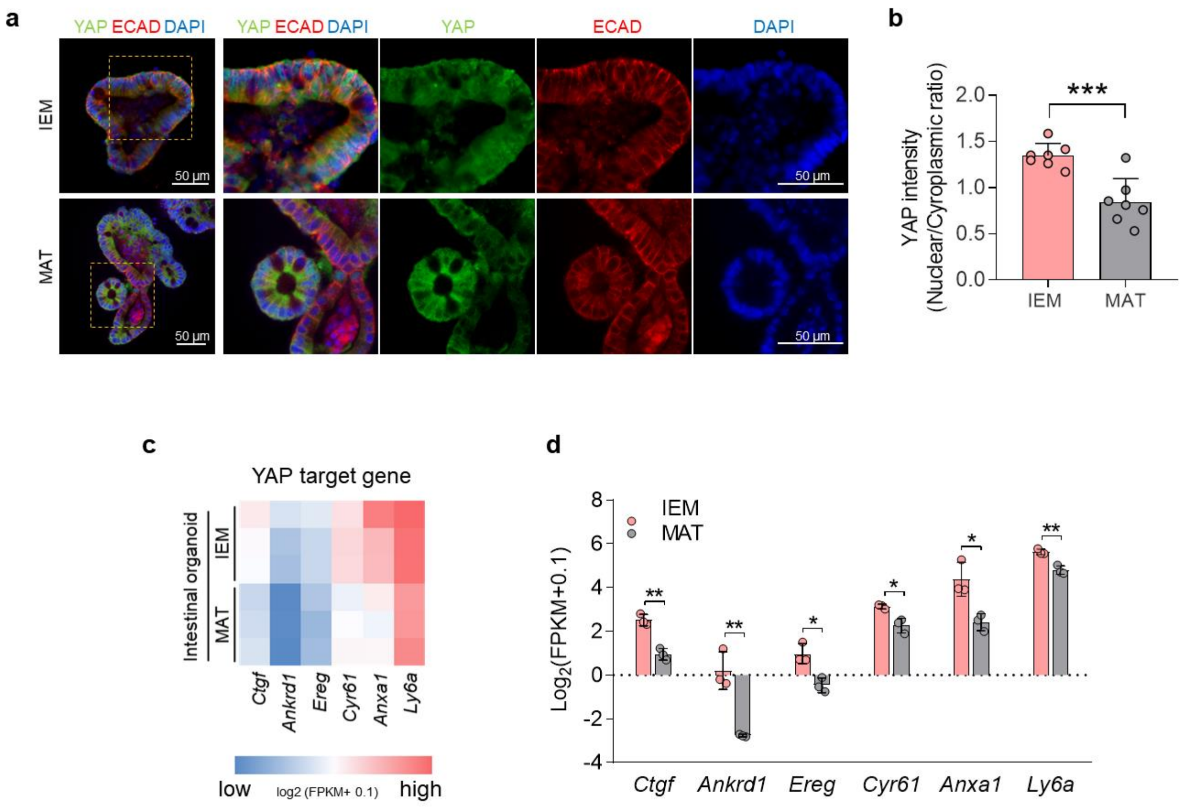

Supplementary Figure 15. YAP signaling activation in intestinal organoids grown in IEM hydrogel. (a) Immunofluorescence staining images of YAP and ECAD in intestinal organoids cultured for 6 days in IEM hydrogel and Matrigel (scale bars = 50 μm). DAPI was used for counterstaining nuclei. (b) Quantification of the nuclear/cytoplasmic ratio of YAP signal in intestinal organoids (two-sided student's *t*-test; IEM versus MAT, ****p* = 0.0006; *N* = 7). (c) Heatmap and (d) graph comparing the expression of several YAP target genes between intestinal organoids grown in IEM hydrogel and Matrigel (two-sided student's *t*-test; IEM versus MAT, ***p* = 0.0018 for *Ctgf*, ***p* = 0.0040 for *Ankrd1*, **p* = 0.0120 for *Ereg*, **p* = 0.0123 for *Cyr61*, **p* = 0.0175 for *Anxa1*, ***p* = 0.0035 for *Ly6a*; *N* = 3). The data in (b) and (d) are presented as mean ± S.D.

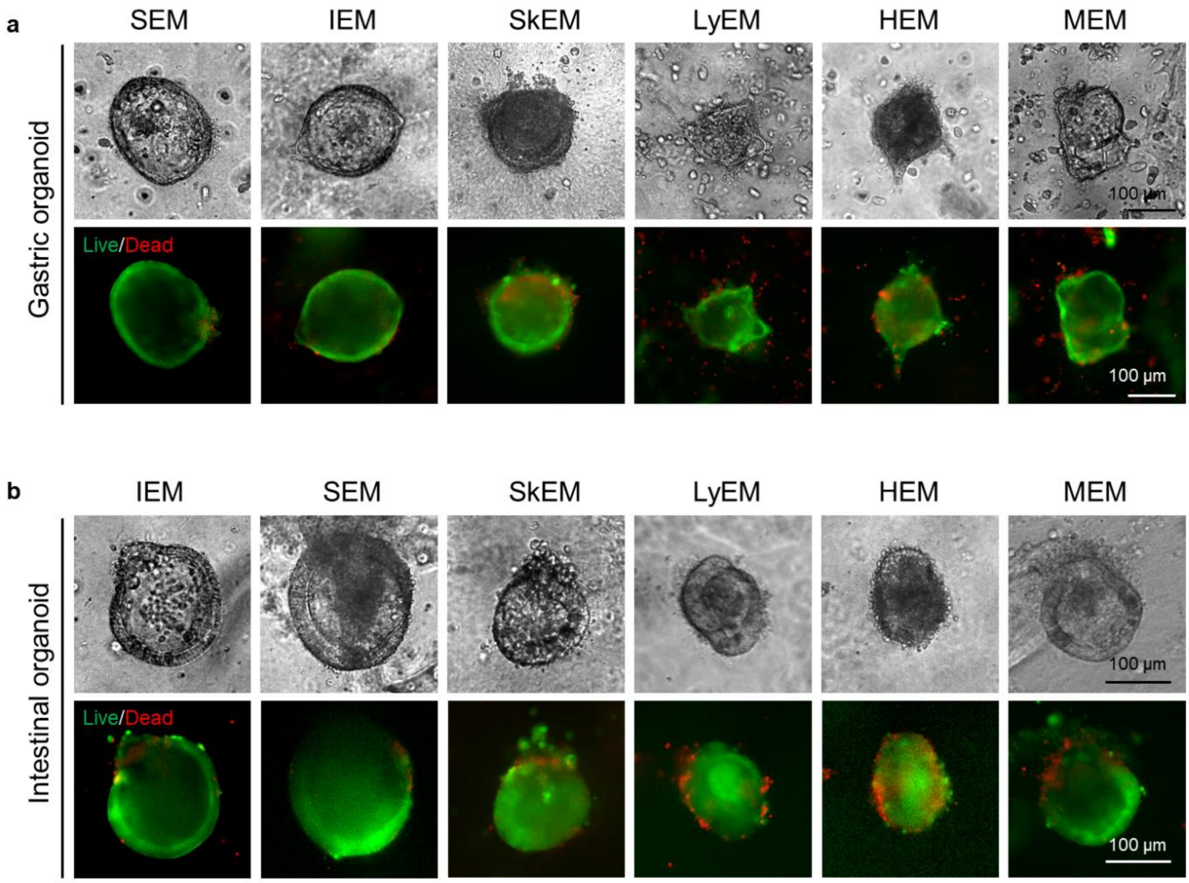

Supplementary Figure 16. Tissue-specific effects of ECM hydrogels on GI organoid culture. Brightfield images and Live/Dead-stained images of (a) gastric and (b) intestinal organoids cultured in various ECM hydrogels prepared with different decellularized tissues (stomach, SEM; intestine, IEM; skin, SkEM; lymph, LyEM; heart, HEM; and muscle, MEM) (scale bars = 100 μm). Gastric organoids and intestinal organoids were cultured for 5 days and 6 days, respectively. Representative images from two independent experiments are shown.

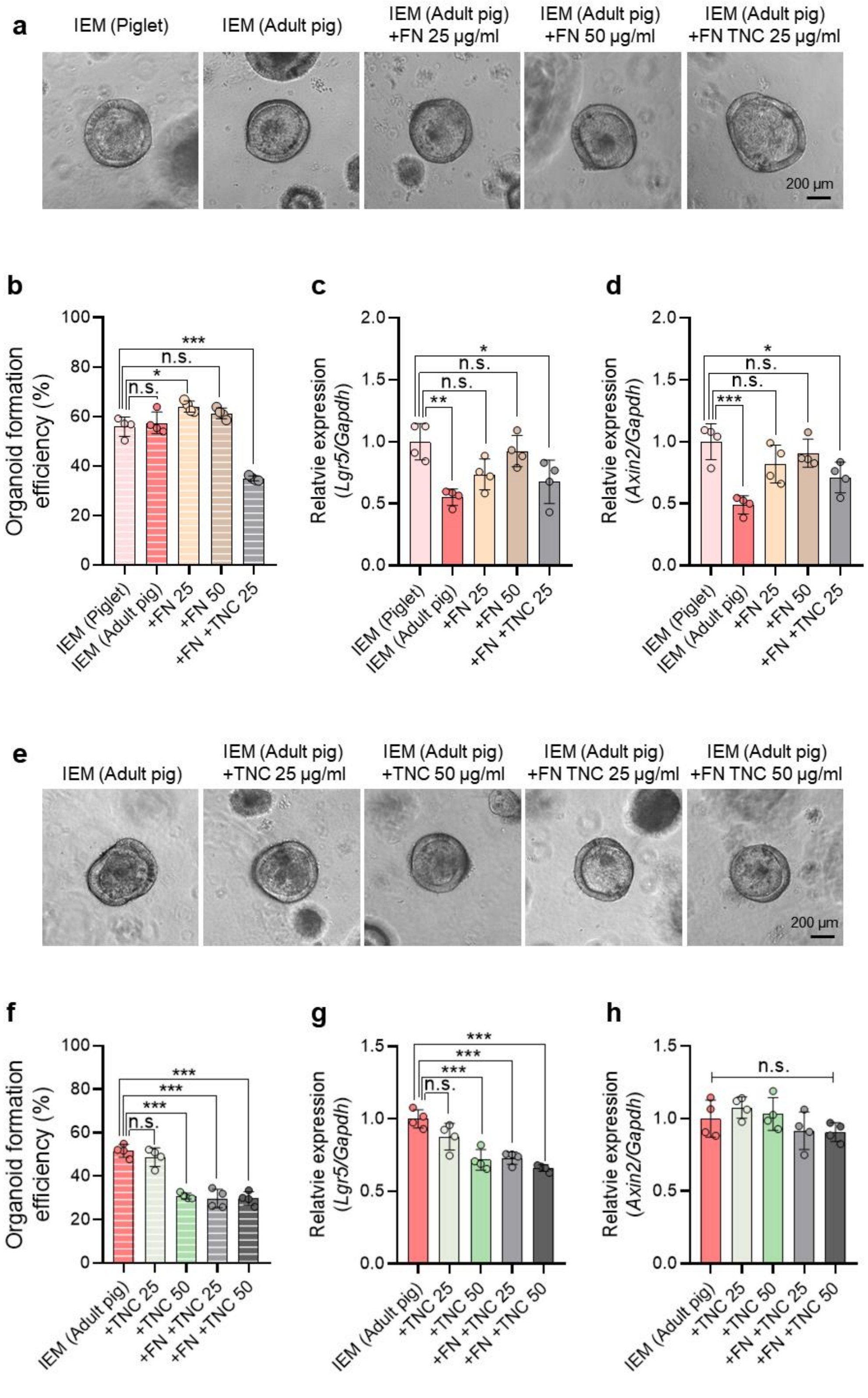

Supplementary Figure 17. The effects of IEM hydrogels supplemented with fibronectin (FN) and tenascin (TNC) in intestinal organoid culture. (a) Brightfield images of intestinal

organoids cultured in IEM (piglet), IEM (adult pig), and IEM (adult pig) hydrogels supplemented with different concentrations of FN (25 and 50 μg ml^{-1}) and/or TNC (25 μg ml^{-1}) (scale bar = 200 μm, independent experiment = 1). (b) Organoid formation efficiency and (c ,d) the expression level of *Lgr5* and *Axin2* in the intestinal organoids grown in each IEM hydrogel (one-way ANOVA with Tukey's multiple comparisons test; (b) IEM (Piglet) versus +FN 25, $*p = 0.0124$; IEM (Piglet) versus +FN +TNC 25, $***p < 0.0001$; (c) IEM (Piglet) versus IEM (Adult pig), $**p = 0.0019$; IEM (Piglet) versus +FN +TNC 25, $*p = 0.0258$; (d) IEM (Piglet) versus IEM (Adult pig), $***p = 0.0003$; IEM (Piglet) versus +FN +TNC 25, $*p = 0.0359$; $N = 4$). (e) Brightfield images of intestinal organoids cultured in IEM (adult pig) and IEM (adult pig) supplemented with different concentrations of TNC (25 and 50 μg ml^{-1}) and FN (25 and 50 μg ml^{-1}) (scale bar = 200 μm, independent experiment = 1). (f) Organoid formation efficiency and (g, h) the expression level of *Lgr5* and *Axin2* in the intestinal organoids grown in each IEM hydrogel (one-way ANOVA with Tukey's multiple comparisons test; (f) IEM (Adult pig) versus +TNC 50, $***p < 0.0001$; IEM (Adult pig) versus +FN +TNC 25, $***p < 0.0001$; IEM (Adult pig) versus +FN +TNC 50, $***p < 0.0001$; (g) IEM (Adult pig) versus +TNC 50, $***p = 0.0001$; IEM (Adult pig) versus +FN +TNC 25, $***p = 0.0002$; IEM (Adult pig) versus +FN +TNC 50, $***p < 0.0001$; $N = 4$). Non-significant statistical difference was indicated as n.s. ($p > 0.05$). Intestinal organoids in these experiments were analyzed at day 6. The data in (b–d) and (f–h) are presented as mean ± S.D.

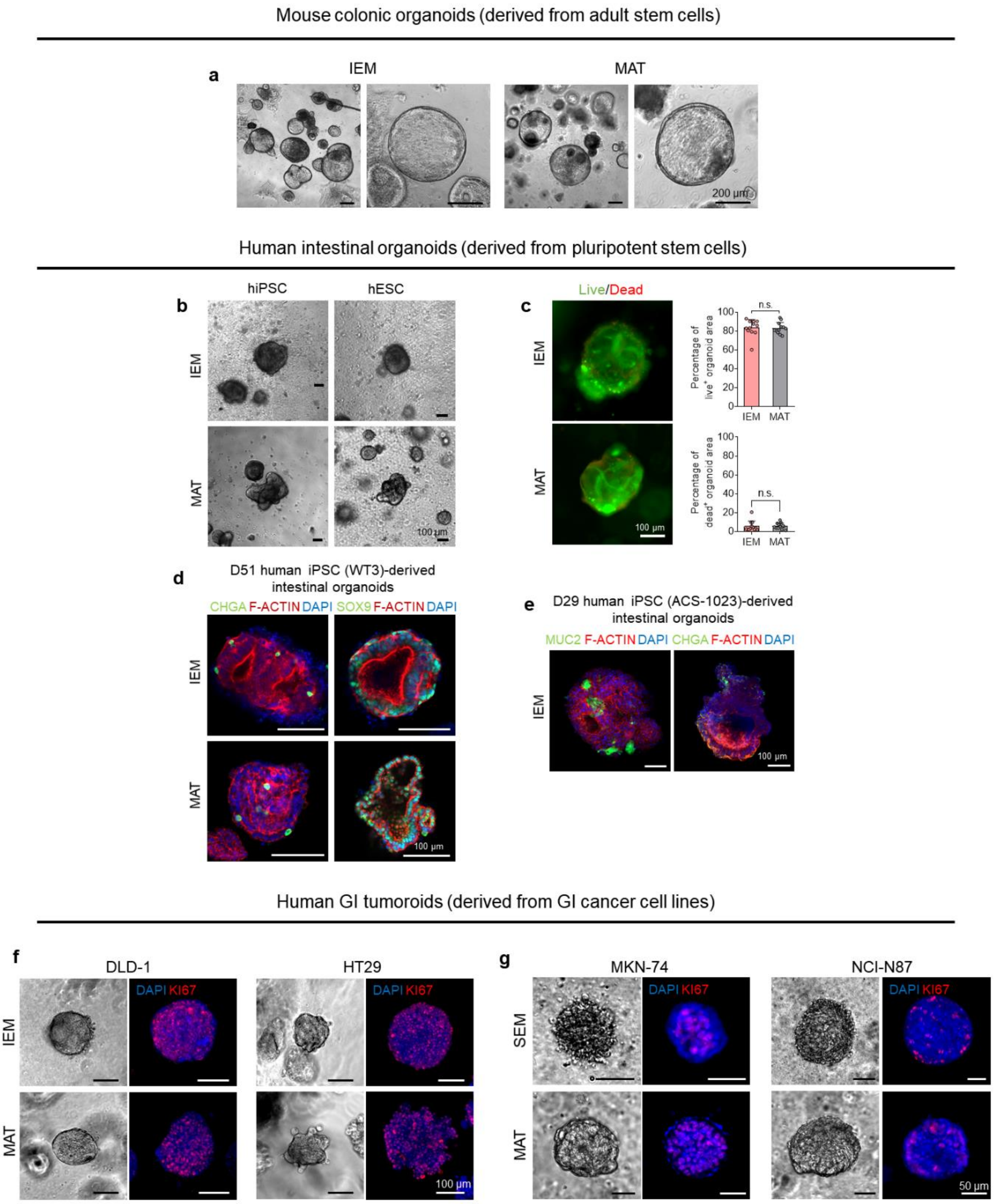

Supplementary Figure 18. Generation of mouse colonic organoids, human pluripotent stem cell-derived intestinal organoids, and GI tumoroids in GI tissue-derived ECM hydrogels. (a) Brightfield images of mouse colonic organoids grown in 2 mg ml^{-1} IEM hydrogel and Matrigel at day 6 in culture (scale bars = 200 µm). Representative images from two independent experiments are shown. (b) Brightfield images of human intestinal organoids

generated from human induced pluripotent stem cells (hiPSCs) and human embryonic stem cells (hESCs) cultured in 2 mg ml^{-1} IEM hydrogel or Matrigel at day 7 (scale bars = 100 μm). Representative images from two independent experiments are shown. (c) Live/Dead staining of human intestinal organoids derived from hESCs in IEM hydrogels or Matrigel at day 6 in culture (scale bar = 100 μm) and quantification of cell viability in human intestinal organoids (two-sided student's *t*-test, $N = 12$). Non-significant statistical difference was indicated as n.s. ($p > 0.05$). Immunofluorescence images of (d) CHGA, SOX9, and F-actin in hiPSC (WT3)-derived intestinal organoids grown in 2 mg ml^{-1} IEM hydrogel and Matrigel at day 51 in culture (scale bars = 100 μm, independent experiment = 1) and (e) MUC2, CHGA, and F-actin in hiPSC (ACS-1023)-derived intestinal organoids grown in 2 mg ml^{-1} IEM hydrogel at day 29 in culture (scale bars = 100 μm, independent experiment = 1). (f) Brightfield images and KI67 immunofluorescence images of intestinal tumoroids generated by culturing intestinal cancer cells (DLD-1, HT29) in 2 mg ml^{-1} IEM hydrogel or Matrigel for 8 days (scale bars = 100 μm). (g) Brightfield images and KI67 immunofluorescence images of gastric tumoroids generated by culturing stomach cancer cells (MKN-74, NCI-N87) in 5 mg ml^{-1} SEM hydrogel or Matrigel for 10 days (scale bars = 50 μm). DAPI was used for nucleus staining in (d)–(g). Representative images from two independent experiments are shown in (f) and (g). The data in (c) are presented as mean ± S.D.

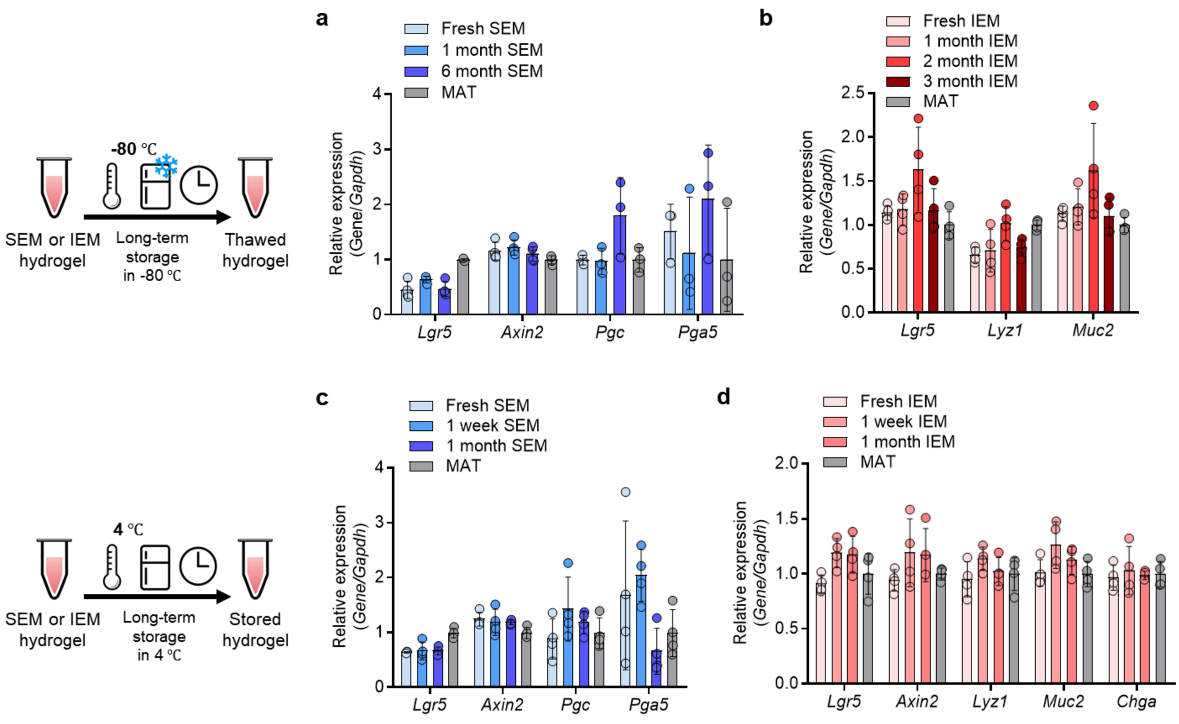

Supplementary Figure 19. qPCR analysis of GI organoids cultured in ECM hydrogels after long-term storage. (a, b) Gene expression of stemness and differentiation markers in GI organoids cultured in fresh Matrigel or ECM hydrogels thawed after long-term storage for 1–6 months at –80°C (N = 3 for (a) and N = 4 for (b)). (c, d) Gene expression of stemness and differentiation markers in GI organoids cultured in fresh Matrigel or ECM hydrogels after long-term storage for 1–4 weeks at 4°C (N = 4). mRNA samples were prepared from gastric and intestinal organoids at day 5 and day 6 in the culture, respectively. The data are presented as mean ± S.D.

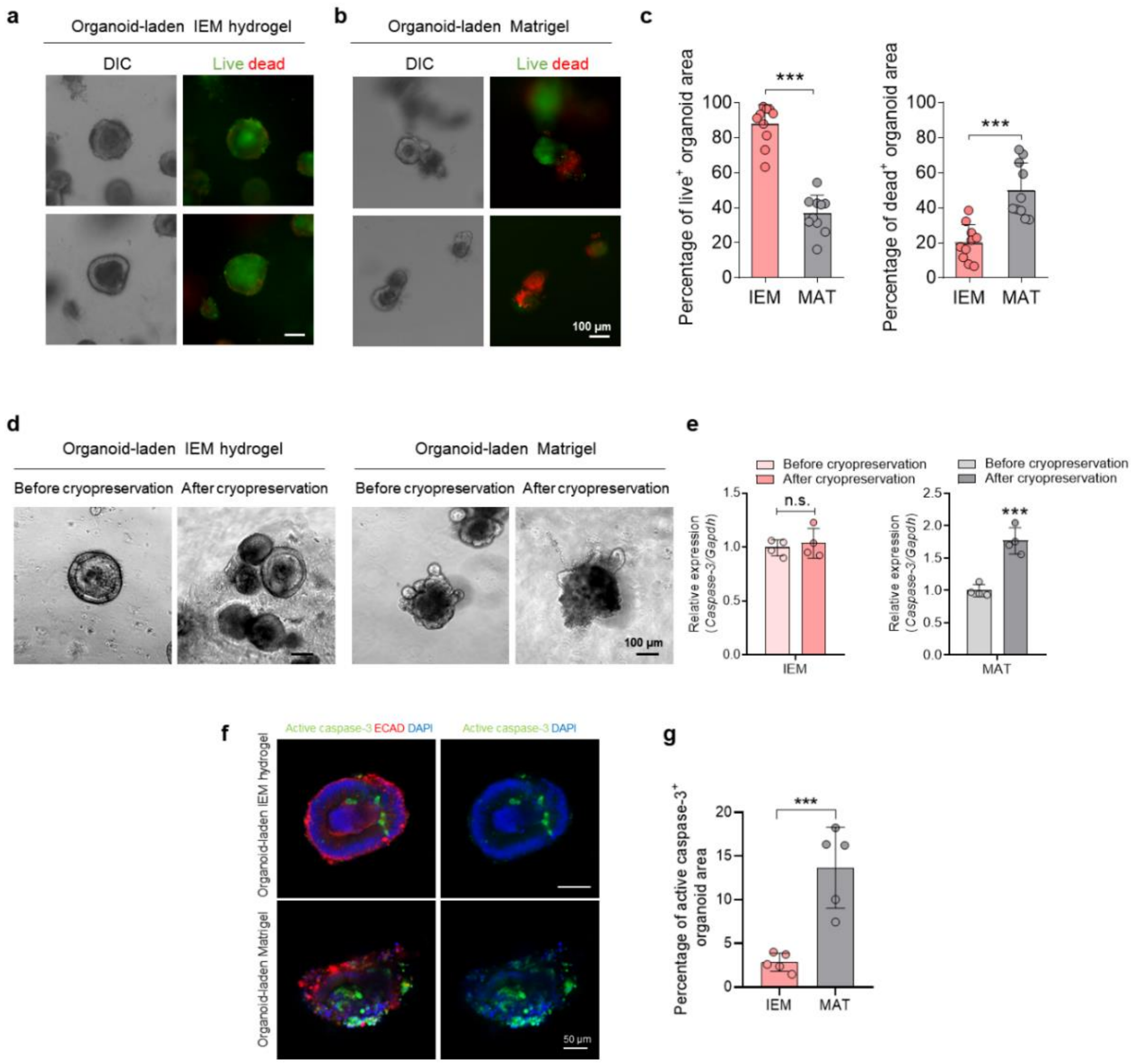

Supplementary Figure 20. Evaluation of organoid-laden ECM hydrogels after cryopreservation. Brightfield images and Live/Dead-stained images of (a) intestinal organoid-laden IEM hydrogels and (b) intestinal organoid-laden Matrigel 5 weeks after cryopreservation (scale bar = 100 μm). (c) Quantification of Live$^+$ and Dead$^+$ areas in organoids 1 day after thawing (two-sided student's *t*-test; IEM versus MAT, ***$p < 0.0001$ for live$^+$ organoid area, ***$p < 0.0001$ for dead$^+$ organoid area; $N = 10$). (d) Brightfield images of intestinal organoid-laden IEM hydrogel or Matrigel before and after 5-week cryopreservation (scale bars = 100 μm). (e) qPCR analysis of *Caspase-3* expression in intestinal organoids before and after 5-week cryopreservation of intestinal organoid-laden hydrogels (two-sided student's *t*-test; Before versus After, ***$p = 0.0005$; $N = 4$). Non-significant statistical difference was indicated as n.s. ($p > 0.05$). (f) Immunofluorescence images of active caspase-3 and ECAD and (g) the

percentage of active caspase-3^+ organoid area in intestinal organoid-laden IEM hydrogel and Matrigel after cryopreservation (two-sided student's *t*-test; IEM versus MAT, ***p = 0.0010; N = 5). DAPI was used for nuclear staining. Representative images from two independent experiments are shown in (a), (b), (d), and (f). The data in (c), (e), and (g) are presented as mean ± S.D.

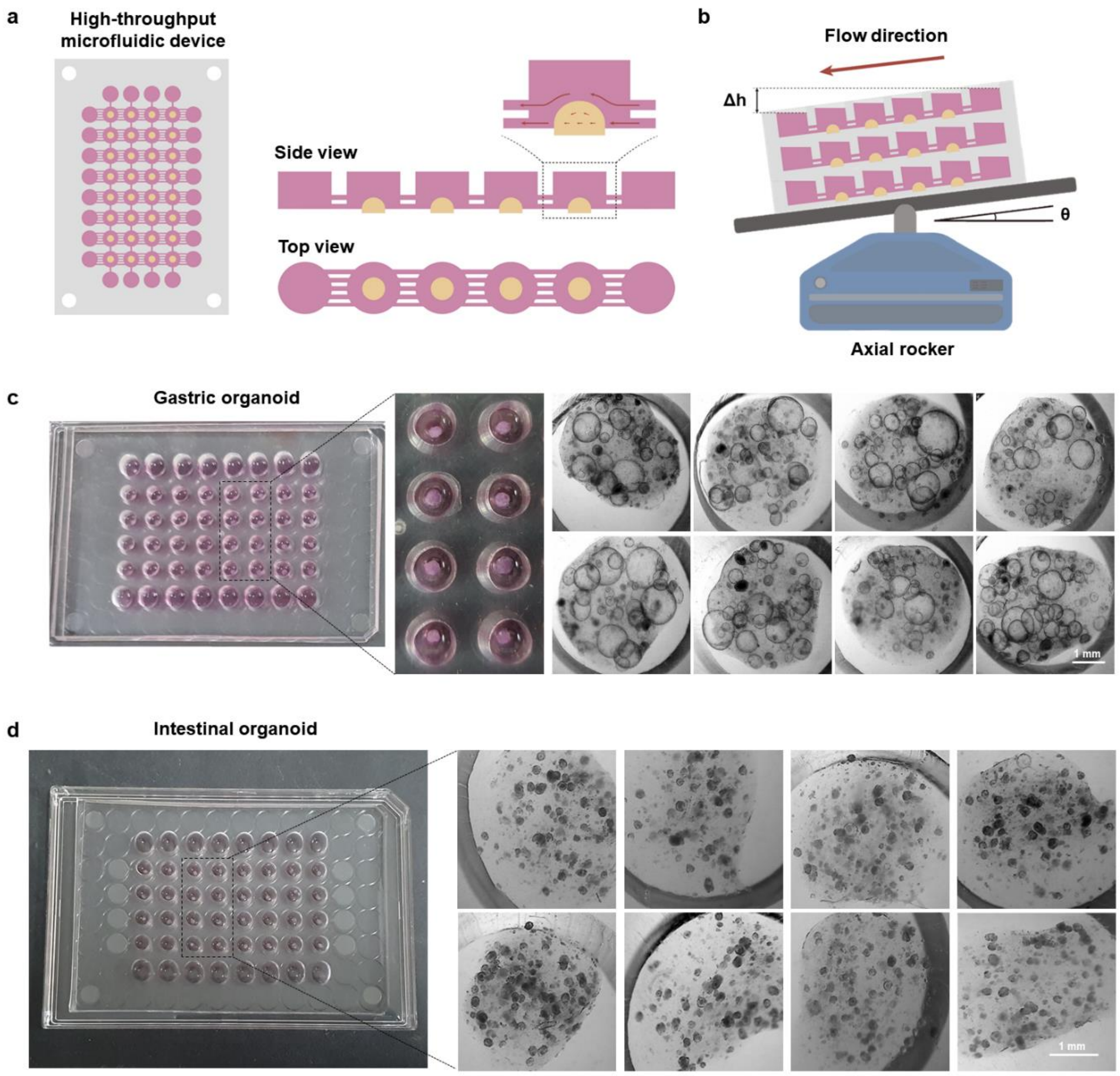

Supplementary Figure 21. Microfluidic system for dynamic culture and mass production of GI organoids in ECM hydrogels. Schematic illustration of (a) chip design and (b) the working principle of the microfluidic system for dynamic culture using a rocker. Mass production of (c) gastric and (d) intestinal organoids in GI tissue-derived ECM hydrogels using the microfluidic system. Gastric and intestinal organoids were observed at day 5 and day 6 in the culture, respectively (scale bars = 1 mm). Representative images from three independent experiments are shown in (c) and (d).

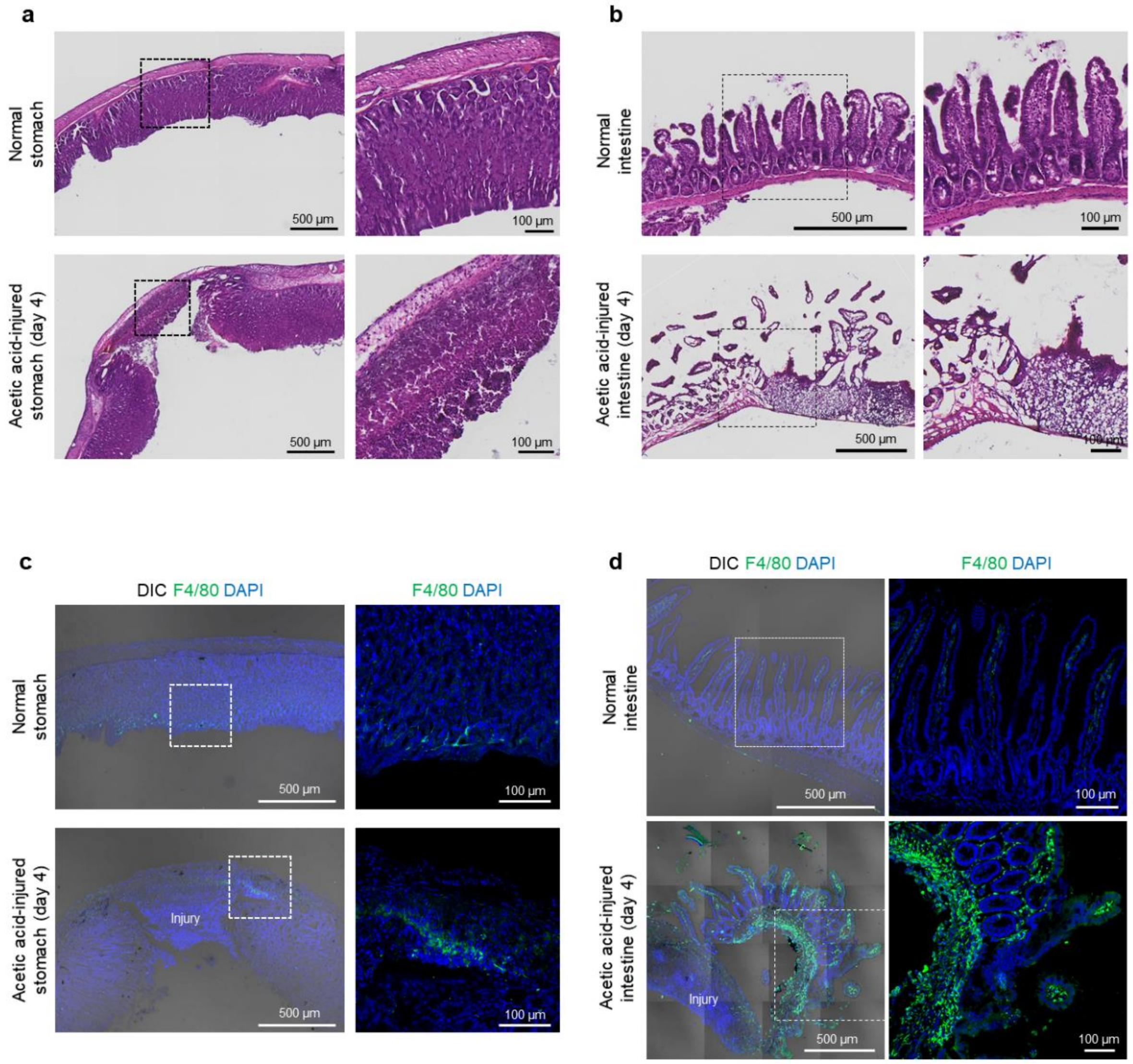

Supplementary Figure 22. Tissue morphology and inflammatory responses in acetic acid-induced GI injury models. H&E-stained images of (a) stomach and (b) intestinal tissues 4 days after injury with acetic acid. Representative images from two independent experiments are shown in (a) and (b). Immunofluorescence staining images for a macrophage marker F4/80 in (c) stomach and (d) intestinal tissues 4 days after injury with acetic acid (independent experiment = 1). Normal stomach and intestine tissues served as control groups to compare tissue morphlogy and inflammation in (a)–(d) (scale bars = 500 µm in left panels and 100 µm in right panels).

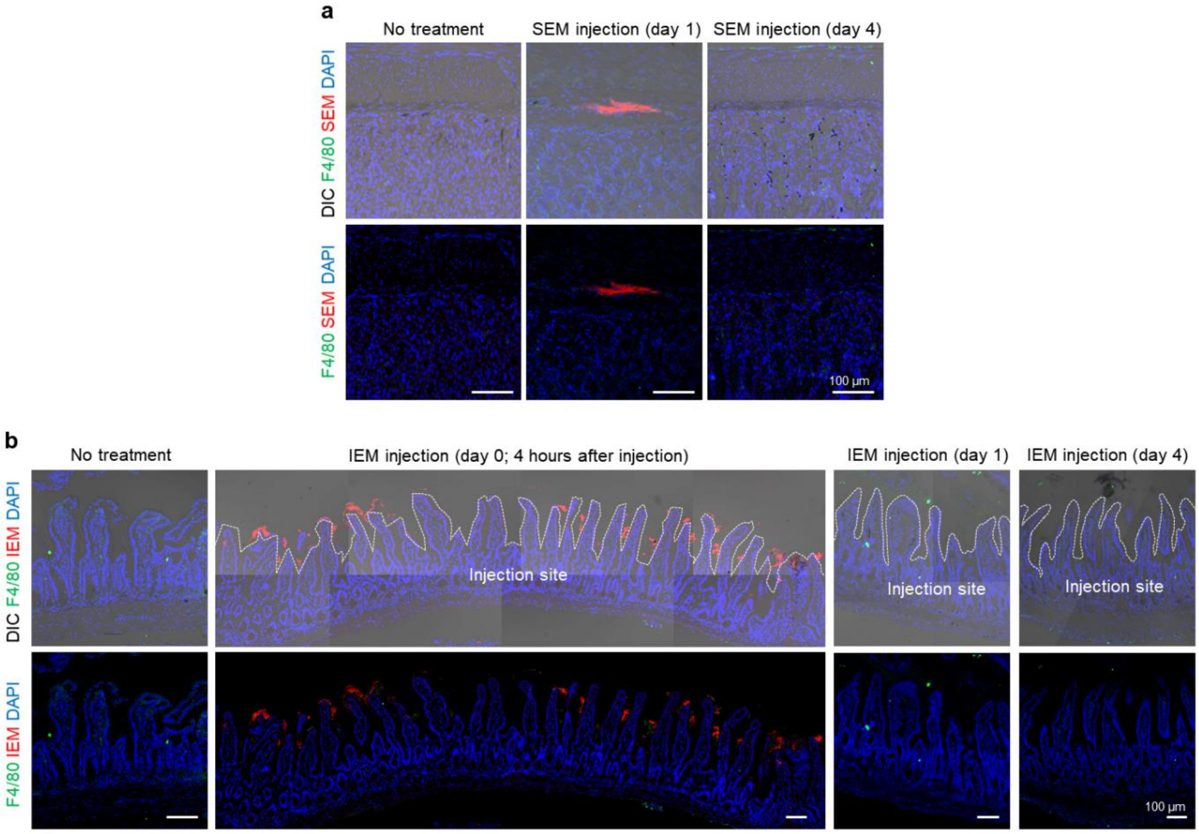

Supplementary Figure 23. Transplantation of TAMRA-SE-labeled ECM hydrogels alone in GI tissues. Fluorescence images of a macrophage marker F4/80 (green) in (a) stomach tissues 1 day and 4 days after injection of TAMRA-SE labeled SEM hydrogel (red) and (b) intestinal tissues 4 hours, 1 day, and 4 days after injection of TAMRA-SE labeled IEM hydrogel (red) (independent experiment = 1). The no treatment group denotes the tissues without hydrogel injection (scale bars = 100 μm). White dotted lines indicate the sites of IEM hydrogel injection.

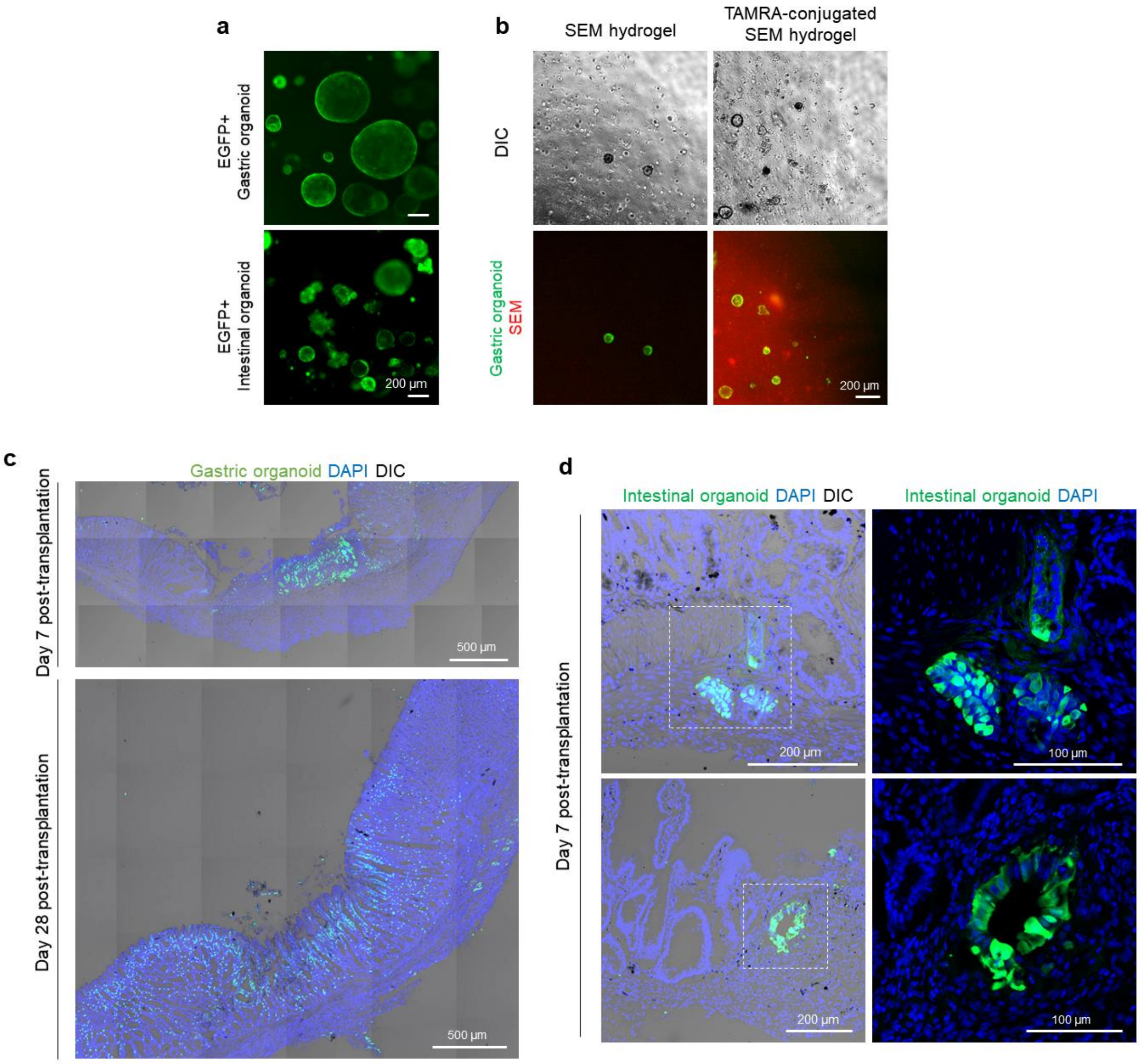

Supplementary Figure 24. Transplantation of EGFP$^+$ mouse GI organoids and TAMRA-SE-labeled ECM hydrogels. (a) Fluorescence images of EGFP$^+$ mouse gastric and intestinal organoids (scale bars = 200 µm, independent experiment = 1). (b) Brightfield images and fluorescence images of EGFP$^+$ gastric organoids encapsulated in SEM and TAMRA-SE-labeled SEM hydrogels (scale bar = 200 µm, independent experiment = 1). (c) Fluorescence images indicating the engraftment of EGFP$^+$ gastric organoids in the injured tissues 7 and 28 days after transplantation into mice with epithelial injury (scale bars = 500 µm). (d) Fluorescence images showing EGFP$^+$ intestinal organoids integrated into injured tissues 7 days after transplantation into mice with epithelial injury (scale bars = 200 µm in left panels and 100 µm in right panels). Representative images obtained with the samples from two independent experiments (two mice per group) are shown in (c) and (d).

Supplementary Table 1. Estimation of the amount and cost of SEM and IEM hydrogels generated from stomach and intestine tissues of one pig.

	SEM	IEM	MAT
Amount of tissue from 1 pig	0.68 kg*	1.43 kg**	-
Tissue weight after decellularization and lyophilization	18 – 20 g	32 – 42 g	-
Optimized concentration of ECM hydrogel for organoid culture	5 mg ml^{-1}	2 mg ml^{-1}	-
Total amount of hydrogel that can be produced from tissue (or total amount of hydrogel in 1 bottle of MAT)	3.6 – 4.0 L	16 – 21 L	10 mL
Amount of hydrogel per well of 48-well plate	30 μL	30 μL	30 μL
Total number of 48-well plates for organoid culture using ECM hydrogel	2,500 – 2,750 plates	11,100 – 14,500 plates	7 plates
Tissue cost (or price of MAT per bottle)	4.40 USD	8.81 USD	317.35 USD
The number of 48-well plates that can be covered with 1$ value of hydrogel	570 plates	1260 plates	Only 1 well of 48-well plate

*Ref. Guise *et al.*, Br Vet J. 1995;151(6):659-70.
**Ref. Loeffel *et al.*, Res. bull. - Univ. Nebr. (Linc. campus), Agric. Exp. Stn. 1970;235.
