# Supplementary material for: Tissue extracellular matrix hydrogels as alternatives to Matrigel for culturing gastrointestinal organoids
Source: Nat Commun. 2022 Mar 30;13:1692. doi: 10.1038/s41467-022-29279-4 (PMC8967832; doi:10.1038/s41467-022-29279-4)
Supplement: Supplementary file 2 — Description of Additional Supplementary Files [file 41467_2022_29279_MOESM2_ESM.docx]

Description of Additional Supplementary Files

Title: Supplementary Data 1

Description: List of total proteins identified from proteomic analysis conducted in this study.
