# Supplementary material for: Tissue extracellular matrix hydrogels as alternatives to Matrigel for culturing gastrointestinal organoids
Source: Nat Commun. 2022 Mar 30;13:1692. doi: 10.1038/s41467-022-29279-4 (PMC8967832; doi:10.1038/s41467-022-29279-4)
Supplement: Supplementary file 4 — Reporting Summary [file 41467_2022_29279_MOESM4_ESM.pdf]

nature portfolio

Corresponding author(s): Seung-Woo Cho

Last updated by author(s): Feb 4, 2022

Reporting Summary

Nature Portfolio wishes to improve the reproducibility of the work that we publish. This form provides structure for consistency and transparency in reporting. For further information on Nature Portfolio policies, see our Editorial Policies and the Editorial Policy Checklist.

Statistics

For all statistical analyses, confirm that the following items are present in the figure legend, table legend, main text, or Methods section.

n/a	Confirmed	
☐	☒	The exact sample size (n) for each experimental group/condition, given as a discrete number and unit of measurement
☐	☒	A statement on whether measurements were taken from distinct samples or whether the same sample was measured repeatedly
☐	☒	The statistical test(s) used AND whether they are one- or two-sided<br>*Only common tests should be described solely by name; describe more complex techniques in the Methods section.*
☒	☐	A description of all covariates tested
☐	☒	A description of any assumptions or corrections, such as tests of normality and adjustment for multiple comparisons
☐	☒	A full description of the statistical parameters including central tendency (e.g. means) or other basic estimates (e.g. regression coefficient) AND variation (e.g. standard deviation) or associated estimates of uncertainty (e.g. confidence intervals)
☐	☒	For null hypothesis testing, the test statistic (e.g. F, t, r) with confidence intervals, effect sizes, degrees of freedom and P value noted<br>*Give P values as exact values whenever suitable.*
☒	☐	For Bayesian analysis, information on the choice of priors and Markov chain Monte Carlo settings
☐	☒	For hierarchical and complex designs, identification of the appropriate level for tests and full reporting of outcomes
☐	☒	Estimates of effect sizes (e.g. Cohen's d, Pearson's r), indicating how they were calculated

Our web collection on statistics for biologists contains articles on many of the points above.

Software and code

Policy information about availability of computer code

Data collection	Microscopy: Ocular 2.0<br>Confocal microscopy: Zeiss Zen 3.0<br>Microplate reader: i-control.2.0<br>qPCR: StepOne™ Software 2.3
Data analysis	Quantification data and statistics: Microsoft Excel (Office 365, 16.0.), GraphPad Prism 8 and 9.1.1<br>Imaging: Microsoft PowerPoint (Office 365, 16.0.), Zeiss Zen 3.0, ImageJ 1.51j8<br>Proteomics: MaxQuant (1.6.10.43), Perseus software 1.6.10.43, g:Profiler (version e104_eg51_p15_3922dba)<br>RNA sequencing: bcl2fastq2 (Version 2.20), Cluster 3.0, Java TreeView 1.2.0

For manuscripts utilizing custom algorithms or software that are central to the research but not yet described in published literature, software must be made available to editors and reviewers. We strongly encourage code deposition in a community repository (e.g. GitHub). See the Nature Portfolio guidelines for submitting code & software for further information.

Data

Policy information about availability of data

All manuscripts must include a data availability statement. This statement should provide the following information, where applicable:

- Accession codes, unique identifiers, or web links for publicly available datasets
- A description of any restrictions on data availability
- For clinical datasets or third party data, please ensure that the statement adheres to our policy

Proteomics data are available at the ProteomeXchange Consortium via the PRIDE partner repository with the dataset identifier PXD023694

[proteomecentral.proteomexchange.org/cgi/GetDataset?ID=PXD023694] and PXD023705 [proteomecentral.proteomexchange.org/cgi/GetDataset?ID=PXD023705]. RNA-sequencing data have been deposited to the Gene Expression Omnibus (GEO) public repository under accession codes GSE165309 [www.ncbi.nlm.nih.gov/geo/query/acc.cgi?acc=GSE165309]. The protein samples were identified by MS/MS data of peptides against the Mouse UniProt database (2020.03 release) for Matrigel and Sus scrofa (pig) UniProt database (2020.03 release) for porcine intestine and stomach tissue. Proteins identified in Matrigel and tissue extracellular matrix hydrogels were compared with the datasets in the Human Protein Atlas [www.proteinatlas.org] and Matrisome Project [matrisomeproject.mit.edu/proteins/]. Raw data for all figures are provided as source data and the lists of total proteins detected by proteomic analysis are provided as supplementary data. All microscopic images and other data generated for this study are available from the corresponding author on reasonable request.

Field-specific reporting

Please select the one below that is the best fit for your research. If you are not sure, read the appropriate sections before making your selection.

- [x] Life sciences
- [ ] Behavioural & social sciences
- [ ] Ecological, evolutionary & environmental sciences

For a reference copy of the document with all sections, see nature.com/documents/nr-reporting-summary-flat.pdf

Life sciences study design

All studies must disclose on these points even when the disclosure is negative.

Sample size	No statistical method was used to predetermine the sample size. Throughout the study, sample size was determined based on our preliminary studies and on the criteria in the field. At least 3 biological samples were included for one experiment and 1 to 3 independent experiments were performed to ensure sufficient reproducibility of the results. Biological replicates (N) and the numbers of independent experiment were indicated within the figure legends.
Data exclusions	No data were excluded from the analysis.
Replication	The number of experiments performed is noted in each figure legend. Most experiments were independently repeated three times and some experiments which are indicated in the manuscript were independently repeated once or twice. Similar results were observed in all repeated trials for each experiment.
Randomization	For in vitro experiments, organoid samples were seeded at the same density and then randomly assigned for analysis. In animal experiments, all mice were housed in same condition and randomly assigned for each group. The injured mice were randomly selected for transplantation of the organoids. For transplantation, total organoids were collected in one tube and divided randomly into same number and volume.
Blinding	The investigators who performed proteomics and RNA-sequencing conducted each analysis without information about the groups. Organoid formation efficiency analysis, quantification of DNA and GAG content, TNF-α secretion test, rheological analysis, qPCR, quantification of the immunostaining images, and quantification of organoid area and circularity were performed in the blind.

Reporting for specific materials, systems and methods

We require information from authors about some types of materials, experimental systems and methods used in many studies. Here, indicate whether each material, system or method listed is relevant to your study. If you are not sure if a list item applies to your research, read the appropriate section before selecting a response.

Materials & experimental systems

n/a	Involved in the study	
[ ]	[x]	Antibodies
[ ]	[x]	Eukaryotic cell lines
[x]	[ ]	Palaeontology and archaeology
[ ]	[x]	Animals and other organisms
[x]	[ ]	Human research participants
[x]	[ ]	Clinical data
[x]	[ ]	Dual use research of concern

Methods

n/a	Involved in the study	
[x]	[ ]	ChIP-seq
[x]	[ ]	Flow cytometry
[x]	[ ]	MRI-based neuroimaging

Antibodies

Antibodies used	Rabbit anti-KI67 (1:1000, ab15580, Abcam)<br>Rabbit anti-SOX9 (1:500, AB5535, Millipore)<br>Mouse anti-MUC5AC (1:200, ab3649, Abcam)<br>Rabbit anti-Chromogranin A (1:200, ab15160, Abcam)<br>Mouse anti-H+/K+-ATPase (1:200, D032-3, MBL International Corporation)<br>Mouse anti-MUC2 (1:200, sc-15334, Santa Cruz Biotechnology)<br>Rabbit anti-Lysozyme (1:250, LYZ; ab108508, Abcam)<br>Mouse anti-VILLIN (1:200, sc-58897, Santa Cruz Biotechnology)

Mouse anti-ECAD (1:200, 14472S, Cell Signaling Technology)
Rabbit anti-ECAD (1:200, 3195S, Cell Signaling Technology)
Rabbit anti-ZO1 (1:50, 61-7300, Thermo Fisher Scientific)
Mouse anti-YAP1 (1:200, sc-101199, Santa Cruz Biotechnology)
Rabbit anti-Cleaved Caspase-3 (1:400, 9661S, Cell Signaling Technology)
Rat anti-F4/80 (1:100, ab6640, Abcam)
Rabbit anti-GFP (1:500, 598, MBL International Corporation)
Anti-mouse Alexa Fluor 488 (1:200, A11001, Thermo Fisher Scientific)
Anti-mouse Alexa Fluor 594 (1:200, A11005, Thermo Fisher Scientific)
Anti-rabbit Alexa Fluor 488 (1:200, A11008, Thermo Fisher Scientific)
Anti-rabbit Alexa Fluor 594 (1:200, A11012, Thermo Fisher Scientific)
Anti-rat Alexa Fluor 488 (1:200, A11006, Thermo Fisher Scientific)

Validation

All antibodies listed above are commercially available and have been verified by many references provided on the website of the companies that sell antibodies.

Rabbit anti-KI67 (ab15580, Abcam) reacts with mouse and human, and has been published and validated for use in immunocytochemistry, as stated on the Abcam website.
Rabbit anti-SOX9 (AB5535, Millipore) reacts with mouse, human, rat, and chicken, and has been published and validated for use in immunocytochemistry, as stated on the Millipore website.
Mouse anti-MUC5AC (ab3649, Abcam) reacts with mouse, human, and rat, and has been published and validated for use in immunocytochemistry, as stated on the Abcam website.
Rabbit anti-Chromogranin A (ab15160, Abcam) reacts with human, and predicted to work with mouse and monkey, as stated on the Abcam website. Several published papers have used this antibody for immunostaining with mouse samples [e.g., Liang et al., FAM3D is essential for colon homeostasis and host defense against inflammation associated carcinogenesis. Nat Commun 11:5912 (2020); Chang et al., Hormonal Suppression of Stem Cells Inhibits Symmetric Cell Division and Gastric Tumorigenesis. Cell Stem Cell 26:739-754.e8 (2020); Roulis et al. Paracrine orchestration of intestinal tumorigenesis by a mesenchymal niche. Nature 580:524-529 (2020)].
Mouse anti-H+/K+-ATPase (D032-3, MBL International Corporation) reacts with mouse, human, rat, and bovine, and has been published and validated for use in immunohistochemistry, as stated on the MBL website.
Mouse anti-MUC2 (sc-15334, Santa Cruz Biotechnology) reacts with mouse, human, and rat, and has been published and validated for use in immunofluorescence, as stated on the Santa Cruz Biotechnology website.
Rabbit anti-Lysozyme (LYZ; ab108508, Abcam) reacts with mouse and human, and has been published and validated for use in immunocytochemistry, as stated on the Abcam website.
Mouse anti-VILLIN (sc-58897, Santa Cruz Biotechnology) reacts with mouse, human, rat, and avian, and has been published and validated for use in immunofluorescence, as stated on the Santa Cruz Biotechnology website.
Mouse anti-ECAD (14472S, Cell Signaling Technology) reacts with mouse, human, and rat, and has been published and validated for use in immunofluorescence, as stated on the Cell Signaling Technology website.
Rabbit anti-ECAD (3195S, Cell Signaling Technology) reacts with mouse and human, and has been published and validated for use in immunofluorescence, as stated on the Cell Signaling Technology website.
Rabbit anti-ZO1 (61-7300, Thermo Fisher Scientific) reacts with mouse, human, rat, dog, and guinea pig, and has been published and validated for use in immunocytochemistry, as stated on the Thermo Fisher Scientific website.
Mouse anti-YAP1 (sc-101199, Santa Cruz Biotechnology) reacts with mouse, human, and rat, and has been published and validated for use in immunofluorescence, as stated on the Santa Cruz Biotechnology website.
Rabbit anti-Cleaved Caspase-3 (9661S, Cell Signaling Technology) reacts with mouse, human, rat, and monkey, and has been published and validated for use in immunofluorescence, as stated on the Cell Signaling Technology website.
Rat anti-F4/80 (ab6640, Abcam) reacts with mouse, and has been published and validated for use in immunocytochemistry, as stated on the Abcam website.
Rabbit anti-GFP (598, MBL International Corporation) reacts with GFP, and has been published and validated for use in immunohistochemistry, as stated on the MBL website.

Eukaryotic cell lines

Policy information about cell lines

Cell line source(s)

Mouse gastric and intestinal organoids was generated from 6–8-week-old C57BL/6 mice (Nara Biotech). L-Wnt-3A cell line (CRL-2647) was purchased from American Type Culture Collection (ATCC) and Rspo1-Fc cells was obtained from Calvin Kuo's Laboratory at Stanford University. Human colon cancer cell lines (DLD-1, HT29) and human gastric cancer cell lines (MKN-74, NCI-N87) were provided from Kang-Yell Choi's Laboratory at Yonsei University. Original source for DLD-1 and HT29 was ATCC and original source for MKN-74 and NCI-N87 was Korean Cell Line Bank. Human embryonic stem cells (hESCs, line WA09) were obtained from the WiCell Research Institute. Human induced pluripotent stem cells (hiPSC, line WT3) were obtained from the Yonsei University College of Medicine and human induced pluripotent stem cells (hiPSC, line KYOU-DXR0109B (ACS-1023)) were obtained from ATCC. Studies involving these cell lines were approved by the Institutional Review Board of Yonsei University (Permit Number: 7001988-202006-ES-904-01E and 7001988-202106-BR-1230-01E).

Authentication

Mouse gastric and intestinal organoids were authenticated with immunostaining to check the expression of stomach cell markers (MUC5AC, CHGA, HK) and intestine cell markers (MUC2, LYZ, CHGA, VILLIN), respectively. Human embryonic stem cells (hESCs, line WA09) and human induced pluripotent stem cells (hiPSC, line WT3 and line KYOU-DXR0109B (ACS-1023)) were authenticated with immunostaining of pluripotency markers (OCT4, TRA-1-60, SOX2) and alkaline phosphatase staining. All cancer cell lines (DLD-1, HT29, MKN-74, NCI-N87) were not authenticated.

Mycoplasma contamination

In each experiment, the results of pathogen monitoring of the animals for primary organoid culture were provided from the

	company. It was confirmed that all animals were negative for mycoplasma infection, indicating that the organoids were free of mycoplasma contamination. Human iPSCs (WT3, ACS-1023), human ESC (WA09), L-Wnt-3A cell line (CRL-2647), Rspo1-Fc cells, and other cancer cell lines (DLD-1, HT29, MKN-74. NCI-N87) were tested and negative for mycoplasma contamination.
Commonly misidentified lines (See ICLAC register)	No misidentified cell lines were used in this study.

Animals and other organisms

Policy information about studies involving animals; ARRIVE guidelines recommended for reporting animal research

Laboratory animals	C57BL/6 mice (6–8-week-old, male) were used for gastric and intestinal organoid culture. C57BL/6-tg (CAG-EGFP, 6-week-old, male) mice were used for generating enhanced green fluorescent protein (EGFP)+ gastric and intestinal organoids. BALB/c-nude mice (CAnN.Cg-Foxn1nu/CrljOri) at 6 weeks of age (male) were used as recipients for organoid transplantation.
Wild animals	Porcine stomach and small intestine tissues were freshly obtained from a local market.
Field-collected samples	No field-collected samples were used in this study.
Ethics oversight	Animal experiments were approved by the Institutional Animal Care and Use Committee (IACUC) of the Yonsei Laboratory Animal Research Center (YLARC) (permit number: IACUC-A-201612-540-04, IACUC-201807-767-03, and IACUC-A-201904-889-02).

Note that full information on the approval of the study protocol must also be provided in the manuscript.
